# Identification, characterization and molecular adaptation of class I redox systems for the production of hydroxylated diterpenoids

**DOI:** 10.1186/s12934-016-0487-6

**Published:** 2016-05-23

**Authors:** Christian Görner, Patrick Schrepfer, Veronika Redai, Frank Wallrapp, Bernhard Loll, Wolfgang Eisenreich, Martin Haslbeck, Thomas Brück

**Affiliations:** Division of Industrial Biocatalysis, Technical University of Munich, Lichtenberg Str. 4, 85748 Garching, Germany; Chair of Bioinformatics, Technical University of Munich, Boltzmannstraße 3, 85748 Garching, Germany; Division of Structural Biology, Institute for Chemistry and Biochemistry, Freie Universität Berlin, Takustr. 6, 14195 Berlin, Germany; Chair of Biochemistry, Technical University of Munich, Lichtenberg Str. 4, 85748 Garching, Germany; Chair of Biotechnology, Technical University of Munich, Lichtenbergstr. 4, 85748 Garching, Germany

**Keywords:** Diterpene biosynthesis, Sinularcasbane D, Cyclooctatin, Redox system, *Streptomycesafghaniensis*

## Abstract

**Background:**

De novo production of multi-hydroxylated diterpenoids is challenging due to the lack of efficient redox systems.

**Results:**

In this study a new reductase/ferredoxin system from *Streptomyces afghaniensis* (AfR·Afx) was identified, which allowed the *Escherichia coli*-based production of the trihydroxylated diterpene cyclooctatin, a potent inhibitor of human lysophospholipase. This production system provides a 43-fold increase in cyclooctatin yield (15 mg/L) compared to the native producer. AfR·Afx is superior in activating the cylcooctatin-specific class I P450s CotB3/CotB4 compared to the conventional *Pseudomonas putida* derived PdR·Pdx model. To enhance the activity of the PdR·Pdx system, the molecular basis for these activity differences, was examined by molecular engineering.

**Conclusion:**

We demonstrate that redox system engineering can boost and harmonize the catalytic efficiency of class I hydroxylase enzyme cascades. Enhancing CotB3/CotB4 activities also provided for identification of CotB3 substrate promiscuity and sinularcasbane D production, a functionalized diterpenoid originally isolated from the soft coral *Sinularia* sp.

**Electronic supplementary material:**

The online version of this article (doi:10.1186/s12934-016-0487-6) contains supplementary material, which is available to authorized users.

## Background

Production of functionalized terpenoids in heterologous microbial hosts allows sustainable and scalable production of bioactive compounds, such as the plant-derived tumor therapeutic taxol or artemisinin [1, 2]. Efficient generation of structurally complex, olefinic terpene macrocycles in heterologous microbial production systems is feasible due to recent advances in synthetic biotechnology. By contrast, the identification and catalytic reconstitution of highly substrate specific P450s, that enable primary regio- and stereoselective hydroxylation of structurally complex olefinic macrocycles, a prerequisite to confer bioactivity on a molecular level, remains challenging. Consequently, only few examples of single hydroxylation reactions have been achieved [3–9].

Native P450s display a high degree of chemo-, regio- and stereoselectivity [4]. In contrast to bacterial class I and III hydroxylases, eukaryotic class II P450s are membrane associated that are characterized by a low solubility when functional reconstitution is carried out in conventional bacterial hosts such as *Escherichia coli* [10]. Moreover, plant-derived class II P450s often yield complex product mixtures [11].

More generally, catalytic efficiency of P450s is highly dependent on electrons delivered by suitable redox partner proteins. In class III P450s the redox partner is fused to the hydroxylase activity. Application of the non-native class III flavocytochrome (P450BM3) system has allowed hydroxylation of various different mono- and sesquiterpenoids [12]. However, larger terpene olefin macrocycles such as diterpenes with an extended degree of chemical complexity, are difficult to be further functionalized by engineered class III P450s [13].

In contrast to the situation with class III P450s, the independent redox partner proteins for most native class I or class II P450s have not been identified, which complicate their functional reconstitution in heterologous microbial hosts. While eukaryotic class II P450s require the presence of a cognate diflavin reductase that incorporates FAD and FMN cofactors (CPR), prokaryotic class I P450s depend on both a reductase and its cognate ferredoxin protein, that together orchestrate the electron transfer (ET) processes. Particularly in class I P450s, the ET process is coordinated through a transient interaction of the respective redox partner proteins, which complicates its comprehensive investigation [14]. Hence, the molecular recognition and structural interaction between redox partners that govern the electron transfer process and class I P450 reactivity are only poorly understood. Notably, functional reconstitution of various class I P450s have been achieved using the well characterized *Pseudomonas putida* reductase·ferredoxin model system (PdR·Pdx), whose cognate hydroxylase is the monoterpene specific P450cam which catalyze the hydroxylation of camphor [15]. Application of the PdR·Pdx system subsequently allowed single step oxidation of substrates [15–17]. However, the interplay of the PdR·Pdx system with non-native P450s is often insufficient, thereby limiting product formation [18]. At present, the inefficient functional reconstitution of P450s has hampered a consolidated, heterologous bio-manufacturing approach for production of bioactive, multi-functionalized diterpenoid macrocycles.

**Fig. 1:**
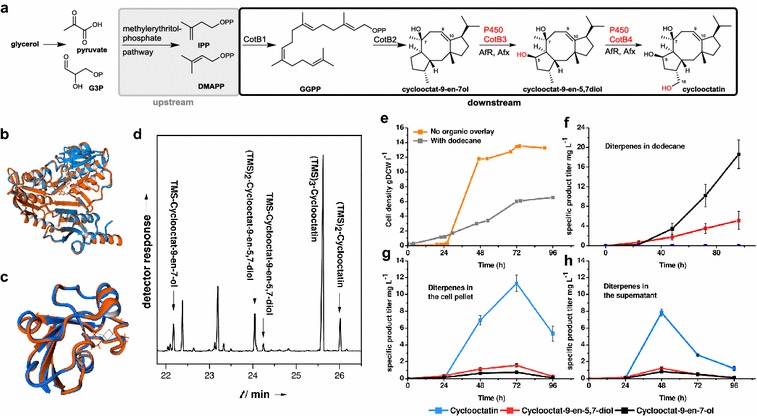
Native and heterologous reconstituted cycloocatin biosynthesis with non-native redox partners. **a** The cyclooctatin biosynthetic pathway encodes a GGDP synthase (CotB1), a diterpene cyclase (CotB2) and two P450 type hydroxylases (CotB3, CotB4). The structure alignments of AfR (*orange*) with PdR (*blue*) (**b)** and Afx (*orange*) with Pdx (*blue*) (**c**) are shown. Despite the low primary sequence similarities structure alignments of PdR with AfR as well as Pdx with Afx have both a root mean square deviation (RMSD) of 0.1 Å over 404 and 106 aligned amino acid residues, respectively. **d** GC–MS chromatogram of a silylated cellular extract from the cyclooctatin producing *E.* *coli* strain harvested after 48 h of growth. **e** Monitored cell density during fermentation is displayed (±dodecane). **f** Production of cyclooctatin and its biosynthetic intermediates in the dodecane organic phase is shown. **g**, **h** Monitored product distribution during fermentation in absence of dodecane

Recently, the endogenous pathway for cyclooctatin (cyclooctat-9-en-5,7-diol) biosynthesis has been reported from *Streptomyces melanosporofaciens* (Fig. 1a). Cyclooctatin is a potential anti-inflammatory agent that targets human lysophospholipase. The sequential cyclooctatin biosynthesis cascade, encodes a geranylgeranyl diphosphate (GGDP) synthase (CotB1), the diterpene synthase CotB2 and the class I P450s CotB3 and CotB4 respectively (Fig. 1a). While the genes for Cotb3 and CotB4 have been reported, their cognate redox systems remain elusive [19].

In this study, we describe the efficient heterologous reconstitution of the cyclooctatin biosynthesis cluster by introduction of a novel, non-native *Streptomyces*-derived redox system. This is the first account for a complete, heterologous biosynthesis cascade of a multi-functionalized diterpenoid in *E. coli*. Our microbial production system allows for a 43-fold increased cyclooctatin yield compared to the native producer *S. melanosporofaciens*. We report on the molecular mechanisms that confer superior activity to our *Streptomyces*-derived redox system, compared to the PdR·Pdx model system. Enhanced hydroxylation efficiency can be achieved by either engineering the P450 activity or respective redox partner proteins [20, 21]. For the first time we engineered the Pdx reactivity on the basis of a significantly sequence divergent template (Afx), which translated into an activity enhancement of the CotB3 activity in excess of 100 %. Enhancement of CotB3/CotB4 reactivity allowed subsequent examination of their substrate promiscuity by introduction of alternative terpene synthases into our bacterial production system. Surprisingly, we could demonstrate that CotB3 can be employed to functionalize other diterpene olefins, such as (−)-casbene to yield sinularcasbane D, which has previously been described from the coral *Sinularia* sp.

## Results

### Identification of suitable redox partners for cyclooctatin biosynthesis

We adopted a multistep computational strategy to identify CotB3/B4 specific redox partners. The *P. putida* derived model class I redox system comprising the reductase (PdR) and ferredoxin (Pdx) proteins was utilized as a template for a homology-based search covering all available *Streptomyces* genomes [22]. Due to the lack of genomic information the native cyclooctatin producer *S. melanosporofaciens* had to be excluded from our bioinformatics screen. One putative PdR and several hypothetical Pdx homologs with up to 45 % similarity to the respective templates clustered specifically within the recently shotgun-sequenced genome of *Streptomyces afghaniensis*. The identified amino acid sequences were subjected to secondary structure analysis using the HHpred server tool [23]. Homology modelling by the HHpred server tool was achieved using the best hits upon HMM (Hidden Markov Model)-HMM comparison including the redox system of *P. putida*, the mitochondrial adrenodoxin redox system [20] and the terpredoxin system from *Pseudomonas* sp. [24]. Subsequent structural alignments of the *S. afghaniensis*-derived reductase and ferredoxin counterparts led to the identification of a NADH-dependent ferredoxin reductase (afghanoredoxin reductase, AfR) and its corresponding 2Fe-2S ferredoxin (afghanoredoxin, Afx) (Fig. 1b, c). While the primary amino sequences of new AfR (45 %) and Afx (38 %) proteins differed significantly from the *P. putida* PdR·Pdx model, their structures could be fitted to their *P. putida* counterparts. To verify the activity of the new redox system, we engineered the entire cyclooctatin biosynthesis cluster into a diterpene producing *E. coli* system.

### In vivo reconstitution of cyclooctatin biosynthesis in *E. coli*

Five day batch, shake flasks (400 mL) cultures did provide several milligrams of cyclooctatin and its biosynthetic precursors for GC–MS, 1D-/2D-NMR, CD spectroscopic and HR-LC/MS spectrometric analyses, which were in full accordance with the published stereochemistry of the compounds (Additional file 1: Figures S3a–i, S9–11) [19, 25]. Interestingly, no product diastereomers were detected, which implies that the CotB3 and CotB4 hydroxylase activities impose absolute product stereoselectivity. As our production system provided sufficient material for detailed structural analysis, we applied Mosher`s NMR methodology to cyclooctatin and confirmed the absolute configuration of the hydroxyl group at C-5 as R (Additional file 1: Figures S3j–t, S4) [26]. Scaling up the cyclooctatin production to 1 L shake flask cultures did not lead to a significant increase in the trihydroxylated product and indicated that the product yield is dependent on controlled oxygen supply. Subsequent cultivation in a 5 L bioreactor system with controlled oxygen supply, allowed generation of 13.7 g/L dry cell weight biomass. Cyclooctatin was the major product, correlated with a maximal cyclooctatin yield of 15 mg/L after 48 h cultivation in LB medium supplemented with 40 g/L glycerol (Fig. 1e–h). By contrast, the cyclooctatin production in native *S. melanosporofaciens* was 0.35 mg/L [19]. Interestingly, the 72–96 h cultivation period was marked by a steadily decreasing cyclooctatin titer, probably caused by oxidative product degradation or by air stripping from the fermentation medium. To further increase cyclooctatin titers we applied organic overlay fermentation, which has been reported to increase yields of other oxygenated terpenoids [6, 10, 20]. The methodology was evaluated in 1 L fermentations supplemented with a 20 % (v/w) dodecane overlay. Analysis of the organic phase indicated that the hydroxylated cyclooctatin precursors cycloocat-9-en-7-ol (23 mg/L) and cyclooctat-9-en-5,7-diol (4.8 mg/L) were the major products. By contrast, only trace amounts of cyclooctatin were detected in the organic layer and the cell pellet, suggesting that the metabolic precursors rapidly transfer from the cell into the organic layer, rendering the hydroxylation cascade ineffective.

### Redox partner promiscuity of CotB3 and CotB4

While the genus *Streptomyces* is known to produce several diterpenoids, *S. afghaniensis* has been reported to produce the antibiotic taitomycin, which is a sulfur containing polypeptide with unknown structure [27, 28]. Recently, a variety of volatile hydroxylated as well as unmodified sesquiterpenoids have been discovered in *S. afghaniensis* [29]. Therefore, AfR and Afx proteins may naturally be involved in sesquiterpene functionalization. Therefore, the functional interaction with both CotB3 and CotB4, which are thought to be specific to cyclooctatin biosynthesis is surprising. Nonetheless, examples of non-cognate eukaryotic or prokaryotic redox partner proteins that are capable to activate *Streptomyces*-derived class I P450s have been reported [30–32]. On a molecular level redox partner promiscuity of class I P450s is currently thought to be dependent on the amino acid decoration of the respective ferredoxin binding site. However, our current understanding of class I redox cascades is incomplete as only three class I P450s in complex with their respective innate ferredoxins have been structurally characterized [14, 33–35].

Therefore, a general mechanistic understanding with respect to partner recognition, ET and possible additional ferredoxin-mediated effector roles remains to be examined. Additionally, the majority of studies examining the ET process on a molecular level have been conducted with the model P450cam·Pdx system [14]. Specifically, Pdx exhibits a unique effector role that induces structural changes in P450cam upon binding [36]. While none of the reported ferredoxin proteins were able to establish P450cam activity [36], it is possible that an endogenous yet unreported ferredoxin homolog was able to establish the function of the P450cam hydroxylase. By contrast, for P450cin, which is responsible for hydroxylation of the monoterpene cineole, a redox partner promiscuity was reported [36]. In this instance, the combination of Pdx and Adr (mitochondrial adrenodoxin) allowed functional activation of P450cin, albeit in slow rates [36]. The recent crystal structure of the P450cin complex with its innate redox partner, a highly unusual FMN-containing flavodoxin (Cdx), demonstrated that P450cin does not depend on structural changes mediated by its redox partner protein [35]. Hence P450cin demonstrates a relaxed redox partner preference compared to P450cam.

**Fig. 2:**
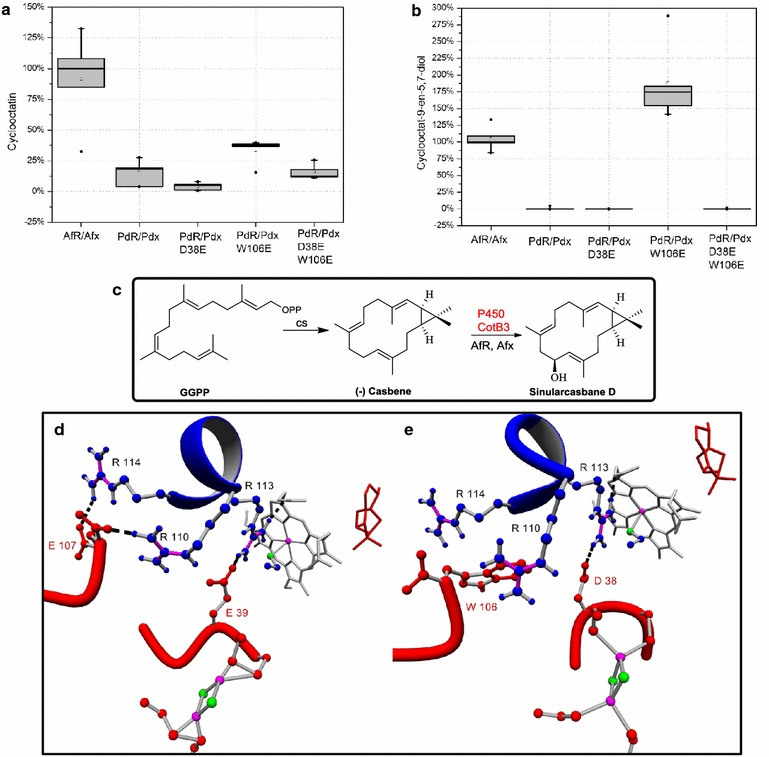
Redox partner induced CotB3/CotB4 substrate promiscuity in a designed *E.coli* production system. **a**, **b** Whole cell hydroxylation experiments using *E. coli* harboring either **a** CotB3 or **b** CotB4 combined with different wild type and mutant redox partners. **a** Cycloocat-9-en-7-ol or **b** cyclooctat-9-en-5,7-diol have been added in vitro. **c** Biocatalytic pathway from GGPP to (−)−casbene by the casbene synthase for *Jatropha curcas* (CS) and subsequent hydroxylation to sinularcasbane D by CotB3. **d** Enlargement of the Cotb3·Afx complex depicting essential molecular interactions. Afx (*red*) contains inorganic Fe2/S2-cluster (*green/magenta*). CotB3 (*blue*) contains prosthetic heme group (*gray*; Fe, *magenta*) and cyclooct-9-en-7-ol (*red*). Hydrogen bonds essential for the successful binding and electron transfer between Afx and CotB3 are shown in *black*. **e** Enlargement of the CotB3·Pdx complex depicting essential molecular interactions. Pdx (*red*) contains inorganic Fe2/S2-cluster (*green/magenta*). CotB3 (*blue*) contains prosthetic heme group (*gray*; Fe, *magenta*) and cyclooct-9-en-7-ol (*red*). Hydrogen bonds essential for the successful binding and electron transfer between Afx and CotB3 are shown in *black*

To elucidate the redox partner promiscuity of CotB3 and CotB4, we tested their activation with the redox system from *P. putida*, as this redox system seems to be structurally very similar to the AfR·Afx system. To compare the efficiency of the monooxygenases CotB3 and CotB4 with the redox systems from *P. putida* and *S. afghaniensis*, whole cell catalysis experiments were carried out. Therefore, *E. coli* strains harboring the pACYC duet vector comprising either CotB3 or CotB4 combined with either AfR-Afx or PdR-Pdx gene cassettes were introduced. Production of cyclooctat-9-en-5,7-diol or cyclooctatin was monitored 48 h after in vitro addition of the corresponding metabolic precursors. In contrast to the AfR·Afx system the PdR·Pdx redox system was not able to generate detectable amounts of cyclooctat-9-en-5,7-diol with CotB3 (Fig. 2a). Additionally, only minor amounts of cyclooctatin with CotB4 could be measured (Fig. 2b). Comparative SDS-PAGE analysis of whole cell extracts and subsequent peptide mass fingerprinting analysis of CotB3/4 with associated redox complexes showed significant differences in expression levels of the two redox systems as well as the P450s (Additional file 1: Figure S1c, d). Specifically, the application of the PdR·Pdx redox system resulted either in a significant overrepresentation of CotB3 or CotB4 and Pdx. By contrast, when the AfR·Afx redox system was applied very low expression levels of CotB3 as well as Afx were detected. Notably, CotB4 expression levels remained the constant when the AfR·Afx redox system was utilized. This cumulative data suggests that there are substantial differences between the AfR·Afx and PdR·Pdx systems on both transcriptional as well as translational levels, which also affect the respective P450 partner proteins. Consequently, we applied an in silico-guided mutagenesis approach to examine the molecular interaction of the AfR·Afx and PdR·Pdx systems with CotB3 and CotB4, respectively.

### Molecular basis of redox partner promiscuity

Recently, key amino acids that coordinate the hydroxylation reaction in the P450cam·Pdx complex have been reported [14, 15, 37]. In this context, the residues Pdx-D38 and Pdx-W106 regulate binding and the two ET processes between Pdx and P450cam. To examine the potential differences of interaction sites in the complexes and resulting ET routes of the AfR·Afx·CotB3/4 versus the PdR·Pdx·CotB3/4 cascades, we applied a comparative structural modelling approach using data of the reported P450cam·Pdx complex as reference.

To evaluate the sequence similarity between the P450 type hydroxylases examined in this study we have conducted a joined structural and sequence alignment. The primary structural alignment of CotB3 with P450cam (PDB-ID: 4JX1) resulted in an RMSD of 0.142 Å over 313 aligned amino acid residues, having 19 % sequence identity. The subsequent alignment of CotB4 and P450cam provided an RMSD of 0.144 Å over 339 aligned amino acid residues with 18 % sequence identity. This data shows that despite low amino acid identity, the overall structural features between the different hydroxylase systems was rather similar. Selected second sphere loops of CotB3/4 that have no match in the P450cam counterparts were not located within the vicinity of the hydroxylase: ferredoxin link and therefore have not been considered in our respective structural alignments. The hypothetical interaction regions of CotB3/4 with Afx/Pdx are part of the matched helical structures (Additional file 1: Figure S2b, d).

The resulting models showed excellent stereochemistry in comparison to the reference structures determined by X-ray crystallography (Additional file 1: Figure S2m–o). Interestingly, our model suggests that the amino acids involved in the interaction of either CotB3 or CotB4 with Afx differ significantly from the P450cam·Pdx Ref. [14, 38]. Specifically, the models indicate the formation of essential hydrogen bonds between CotB3/4-R110/-R111·Afx-E107, CotB3/4-R113/-R114·Afx-E39 and CotB3/4-R114/-R115·Afx-E107 (Fig. 2d; Additional file 1: Figure S2i). Structural superposition of CotB3/4·Afx with P450cam·Pdx established the structural analogy of Afx-E39 with Pdx-D38 as well as Afx-E107 with Pdx-W106. CotB3/4-R110/-R111·Afx-E107 and CotB3/4-R113/-R114·Afx-E39 interactions are apparently analogous to P450cam-R109·Pdx-W106 and P450cam-R112·Pdx-D38, respectively [37]. However, the hydrogen-bond based interaction between CotB3/4-R114/-R115 and Afx-E107 detected in silico, significantly differs from the hydrophobic interaction between P450cam-A113 and Pdx-W106. These amino acids are essential for binding of redox partners in P450cam·Pdx and the two ET steps [14]. Accordingly, models of CotB3/4·Pdx do not show an interaction between R114/R115 and Pdx-W106 (Fig. 2e). These findings argued for a polar interaction between CotB3/4-R114/-R115 and Afx-E107, presumably participating in CotB3/4·Afx binding and the ET processes analogous to its *P. putida* equivalent.

Guided by the Afx residues, we modified either Pdx-D38 and/or -W106 to verify our modelling approach, resulting in a series of Pdx mutants (Pdx-D38E, Pdx-W106E and Pdx-D38E/W106E). As Afx expression levels were generally very low, in vitro reconstitution of the P450·Afx systems was not possible. Therefore, we decided to evaluate our in silico-guided, optimization of CotB3/4 activation by the mutant Pdx ferredoxins utilizing whole cell biocatalysis approach. In our engineered diterpene production system we replaced native Pdx by respective variant forms and then evaluated the resulting alteration in terpene product formation by whole cell catalysis. Surprisingly, the Pdx-W106E mutant significantly increased cyclooctat-9-en-5,7-diol formation compared to the Afx control (Fig. 2a). The Pdx-W106E mutant also led to increased cyclooctatin formation, indicating a successful interaction with both CotB3 and CotB4 (Fig. 2b). Pdx-D38E and Pdx-D38E/W106E, however, did not allow the production of cyclooctat-9-en-5,7-diol and did not increase cyclooctatin titers compared to the Pdx reference control. Whole cell SDS-PAGE analysis of Pdx-D38E and Pdx-D38E/W106E combined with peptide mass fingerprinting of P450-associated protein complexes corroborates these results (Additional file 1: Figure S1c, d). Interestingly, the expression levels of Pdx-W106E and CotB3 decreases to the level of the CotB3·Afx complex, which was confirmed by multiple repeats and resequencing of the constructs. In contrast, expression levels of CotB3·Pdx-D38E and CotB3·Pdx-D38E/W106E complexes were similar in respect to the expression of the wild type CotB3·Pdx complex. For the CotB4·Pdx-mutant complexes almost no alteration in expression levels could be detected compared to wild type CotB4·Pdx complex.

### Substrate promiscuity of class I CotB3/4 hydroxylase activities

Efficient reactivity of class I P450 hydroxylases strictly depends on the use of the suitable redox partner protein. By contrast, their substrate promiscuity is dependent on the amino acid decoration of hydroxylase active site. As CotB3 and CotB4 have not yet been characterized with respect to their substrate promiscuity we examined the application of CotB3 and CotB4 with respect to hydroxylation of alternative diterpene macrocycles. Therefore, we exchanged the diterpene synthases CotB2 with alternative diterpene synthases in vitro as well as in our heterologous terpene production system. Consequently, R-cembrene A [39], (−)−casbene [40], (1R,3E,7E,11S,12S)-3,7,18-Dolabellatriene [39] and taxa-4,5-11,12-diene [41] were evaluated as alternative hydroxylation substrates. Surprisingly, CotB3 was able to hydroxylate 20 % of (−)−casbene yielding 1.4 mg/L of a single mono-hydroxylated product (Fig. 2c). GC–MS and 1D-/2D-NMR analysis, allowed identification of the compound as sinularcasbane D (Additional file 1: Figures S6, S7, S8a–g, S10) [42] This is the first report of a recombinant, stereoselective formation of sinularcasbane D, which has initially been isolated from the soft coral *Sinularia* sp. [42]. The biological activity of hydroxylated casbene diterpenoids has not been extensively explored. However some members of this group display moderate cytotoxicity and antimicrobial activity [42]. Molecular docking of (−)−casbene into the CotB3 active site, established the optimal positioning for hydroxylation at C-10 (Additional file 1: Figure S2k).

## Discussion

Identification of the novel AfR·Afx redox system from *S. afghaniensis*, allowed efficient reconstitution of the CotB3 and CotB4 hydroxylation activities, which enabled the first de novo biosynthesis of the trihydroxylated diterpene cyclooctatin in *E. coli*. The focus of the current study to establish efficient hydroxylation steps of cyclooctatin precursors to enhance the final product titer. To our knowledge this is the first example of a consecutive P450-mediated hydroxylation cascade described for a diterpene macrocycle in *E. coli*. What is more, our rather simple production system provided for a 43-fold increase in cyclooctatin yield compared to the native producer *S. melanosporofaciens*. The novel AfR·Afx redox system was superior in activating CotB3/CotB4 hydroxylation activities compared to the *P. putida*-derived PdR·Pdx model. Since AfR and Afx proteins show significant primary sequence divergence compared to their PdR·Pdx counterparts, the former may be particularly suitable to activate various *Streptomyces*-derived monooxygenase systems.

The molecular basis for this stimulating effect was examined by in silico-guided mutagenesis studies. This methodology allowed experimental identification of Afx residues E39 and E107, which interface with partner amino acids of the respective P450s. In silico-guided mutagenesis at the equivalent CotB3/4 binding site of Pdx did assume enhanced protein·protein interaction and ET capabilities. Therefore, the W to E substitution in Pdx-106 presumably translated to enhanced CotB3 activities in excess of 100 %. As Afx expression levels are generally very low, a detailed in vitro characterization was not possible. Therefore, we at least applied whole cell biocataylsis experiments together with comparative SDS-PAGE analysis to test our in silico-derived hypothesis. Our results clearly indicate that the Pdx-W106E substitution is superior in activation of CotB3 compared to the Afx and Pdx wild type ferredoxins. An unexpected result, however, was the decrease in the expression level of CotB3 and Pdx-W106E to the expression of Afx and CotB3 in the comparative SDS-PAGE analysis. We are not able to explain this fact, but despite the lower expression level of Pdx-W106E and CotB3 in this specific experiment, the system shows a 100 % increase in cyclooctat-9-en-5,7-diol production. Therefore, this the first experimental indication of non-cognate redox partner engineering based on the induced fit model reported for the Pdx·P450cam interaction [14].

Nonetheless, caution is required in mutating essential amino acid residues at the P450 binding site of ferredoxins as these residues also play a role in determination of the correct binding mode with and participate in ET from the NADH-dependent reductase. The surprising decrease in productivity of the Pdx-D38E and Pdx-D38E/W106E constructs might be explained by a reduced interaction with PdR. As binding and ET between PdR and Pdx rely on a sensitive interaction between PdR-R310 and Pdx-D38, the Pdx-D38E substitution apparently leads to a significant attenuation of these processes, which is in line with previous reports [37]. This, in turn, argues for differences in binding and ET processes in the AfR·Afx compared to PdR·Pdx complex. A structural superposition of PdR·Pdx with the modelled AfR·Afx complex highlights these differences (Additional file 1: Figure S2j). In addition to the identified key residues in Afx, E39 and E107 that are presumably important for partner recognition with and ET to CotB3/4, other residues comprising the binding surface between the P450 and Afx have to be taken into account to further optimize this system. A recent report on P450cam·Pdx interaction demonstrated that in addition to essential amino acid residues involved in redox partner recognition and ET, other interactions contribute to the initial binding of both proteins [43]. Therefore, we conclude that the reason for the slightly lower increase of product titers in the CotB4·Afx and CotB4·Pdx-W106E system compared to the CotB3·Afx and CotB3·Pdx-W106E system may be due to differing binding modes of CotB3 and CotB4 with Afx, inaccessible by in silico complex modelling.

Our data indicate that the exchange of Pdx-W106 to an amino acid that is capable of binding with CotB3/4 and facilitating ET is the molecular basis for the increased product formation. Accordingly, adjustments of the amino acid D38 in Pdx might also lead to more efficient binding to CotB3/4 and higher catalytic yields. However, as in such a scenario the interaction of Pdx and PdR is disturbed, higher catalytic yields were not observed. An alternative scenario to enhance the binding and ET efficiency is to alter the ferredoxin binding site of the respective P450 [20]. Nonetheless, other native and non-native class I P450 systems have to be evaluated to delineate a generally applicable approach for the improvement of biotechnological processes that are dependent on class I redox-couples. However, our current methodology could assist in identifying a general solution for the adaptation of class I redox systems to any non-native P450.

## Conclusions

Identification of the AfR·Afx system was essential to reconstitute the cyclooctatin-specific P450s CotB3/CotB4. Elucidation of the molecular basis for the AfR·Afx superior activity allowed adaptation of the PdR·Pdx model, which in turn resulted in further enhanced CotB3 activity. Conventionally, enzymatic catalytic turnover of non-native substrates is less efficient compared to the native counterpart [43, 44]. Hence, the transformation of alternative substrates is commonly associated with low yields, which negatively impact respective product detection. In this respect efficient ET from the ferredoxin partner to the P450 active site can facilitate enhanced turnover kinetics and space–time product yields. In turn, enhanced formation of alternative products improve their analytical detection. We evaluated CotB3/CotB4 substrate promiscuity by exchanging diterpene synthase constructs in our heterologous production system. Surprisingly, CotB3 was able to hydroxylate (−)−casbene, which is structurally unrelated to cycloocat-9-en-7-ol, its native substrate. CotB3 substrate promiscuity allows for an efficient and stereoselective production of sinularcasbane D, a functionalized diterpenoid from the soft coral *Sinularia* sp. This data indicates that the P450s CotB3 and CotB4 may be valuable protein engineering targets to enable single functionalization of other diterpenes. Hence CotB3/CotB4 engineering would extend the current repertoire of recombinant hydroxylase activities available to functionalize terpene olefin macrocycles. Moreover, optimizing ETs for other class I hydroxylase systems by directed redox partner engineering may allow a more comprehensive study towards their substrate promiscuity. Further, the entire CotB3·CotB4·AfR·Afx cascade may be a valuable system for multi-functionalization of alternative olefinic macrocycles.

## Methods

### General experimental procedure

Chemicals used in this study were obtained from standard sources at the highest purity grade available. NMR spectra were recorded in CDCl_3_ or CD_3_OD with an Avance-III 500 MHz (Bruker) at 300 K. ^1^H NMR chemical shifts are given in ppm relative to CHCl_3_ (*δ* = 7.26 ppm) or CD_2_HOD (*δ* = 3.31 ppm) and CD_3_OH (*δ* = 4.87) (^1^H NMR). ^13^C NMR chemical shifts are given in ppm relative to CDCl_3_ at *δ* = 77.16 ppm or CD_3_OH at *δ* = 49.00 ppm. The 2D experiments (HSQC, COSY, NOESY) were performed using standard Bruker pulse sequences and parameters.

GC–MS analysis of diterpene products from ethyl acetate extractions was done by a Trace GC Ultra with DSQII (Thermo Scientific). Probes were silylated (1 h at 70 °C) prior to measurement by 37.5 % (v/v) N,O-bis(trimethylsilyl)trifluoroacetamide (BSTFA) and 25 % (v/v) chlorotrimethylsilan (TMS-Cl) in pyridine. For the GC–MS analysis of dodecane probes, 50 μL of the dodecane probe was mixed with 45 μL pyridine, 45 μL N,O-bis(trimethylsilyl)trifluoroacetamide (BSTFA) and 10 μL chlorotrimethylsilan (TMS-Cl). Silylation was done for 4 h at 70 °C. One microlitre sample was applied by TriPlus AS onto a SGE BPX5 column (30 m, I.D 0.25 mm, Film 0.25 μm). The initial column temperature started at 50 °C and was maintained for 2.5 min. Next, a temperature gradient was applied from 50 to 320 °C (10 °C/min), followed by 3 min maintenance at 320 °C. MS data were recorded at 70 eV (EI), *m/z* (rel. intensity in %) as TIC, total ion current. The recorded *m/z* range covered 50–650. Quantification was done by using flame ionization detector (FID) using 1 mg/mL α-humulene (Sigma-Aldrich, Germany) as an internal standard.

To apply the FID signal of α-humulene to the silylated diterpene products, correlation factors were calculated according to the literature [45]. From the determined mass concentrations of the silylated diterpene products the mass concentrations of the non-derivate products were calculated.

High-resolution mass spectra of diterpenes were determined with Thermo Fisher Acela HPLC system linked to a Thermo Fisher Scientific LTQ Orbitrap XL mass. Electrospray ionization was done in positive ion mode.

MALDI-MS analysis using an Ultraflex I (Bruker Daltronics) was applied to identify the recombinant expressed proteins in the observed bands on SDS-PAGE. Bands were excised and digested with trypsin as described previously [46]. Prior to MS-analysis, peptides were concentrated and purified using C18 ZipTips (Merck Millipore) following the manufacturer’s protocol. Data analysis was performed using the MASCOT software program (Matrix Science, London, UK) along with the National Center for Biotechnology Information database.

Circular dichroism (CD) spectroscopy was performed using a Chirascan plus spectropolarimeter (Applied Photophysics). Samples were dissolved in acetonitrile and spectra were recorded in quartz cuvettes with 0.1 cm path length at 20 °C.

### Bacterial strains, genes and vectors

The *E. coli* strain XL-1 Blue was used for cloning and Bl21 (DE3) was used for the diterpene production. All strains and plasmids were obtained from Novagen/Merk Millipore. Genes were synthesized by Life technologies GmbH containing the appropriate restriction sites and adjusting codon usage for *E. coli*.

### Plasmids for the cyclooctatin production

The 1-deoxy-d-xylulose 5-phosphate (DXP) pathway was housed by the plasmids pCola-Duet-1 and pCDFDuet-1, while the cyclooctatin biosynthesis genes were carried by the plasmids pET-Duet-1 and pACYC-Duet-1. To overexpress the DXP pathway in *E. coli* BL21(DE3), genes from *E. coli* of the 1-deoxy-d-xylulose 5-phosphate synthase (*dxs*) (GenBank: YP_001461602.1), 1-deoxy-d-xylulose 5-phosphate reductoisomerase (*dxr*) (GenBank: NP_414715.1), 2-C-methyl-d-erythriol 4-phosphate cytidyltransferase synthase (*ispD*) (GenBank: NP_417227.1), 2-C-methyl-d-erythritol 2,4-cyclodiphosphate synthase (*ispF*) (GenBank: NP_289295.1) and Isopentenyl-diphosphate delta isomerase (*idi*) (GenBank: NP_417365.1) were synthesized. *IspD/ispF* was created as a bi-cistronic operon. The synthetic genes were introduced into the appropriate plasmids according to Table 1 by standard cloning techniques.

**Table 1:** Plasmids used to construct the overexpressed DXP pathway in *E. coli* Bl21 (DE3)

Gene(s)	Vector	Multiple cloning site	Restriction sites
*dxr*	pCola-Duet-1	I	NcoI, EcoRI
*dxs*	pCola-Duet-1	II	NdeI, XhoI
*ispD/ispF operon*	pCDF-Duet-1	I	NcoI, EcoRI
*idi*	pCDF-Duet-1	II	NdeI, XhoI

To achieve biosynthesis of cyclooctatin the native geranylgeranyl diphosphate synthase (*crte*) (GenBank: M90698.1) sequence was amplified from *Pantoea agglomerans* (ATCC 27155) using standard protocols. Primers used were 5′-AAA CCA TGG CAA TGG CAA CGG TCT GCG CA-3′ and 5′-AAA GAA TTC TTA ACT GAC GGC AGC GAG TTT-3′. Genes of *cotB2* (GenBank: BAI44338.1), *cotB3* (GenBank: BAI44339.1), *cotB4* (GenBank: BAI44340.1) from *Streptomyces melanosporofaciens* MI614-43F2 as well as the genes to express the reductase (*afR*) (GenBank: WP_020277402) and ferredoxin (*adx*) (GenBank: WP_020276845) from *Streptomycesafghaniensis* were synthesized. *CotB3*/*B4*, *afR*/*afx* and *pdR*/*pdx* from *Pseudomonas putida* were expressed from bi-cistronic operons, introduced, respectively, into the appropriate plasmids according to Table 2 by standard cloning techniques.

**Table 2:** Plasmids used to construct the cyclooctatin biosynthesis in *E. coli* Bl21(DE3)

Gene(s)	Vector	Multiple cloning site	Restriction sites
*crte*	pET-Duet-1	I	NcoI, EcoRI
*cotB2 or txs or cs*	pET-Duet-1	II	NdeI, XhoI
*afR/afx operon or pdR/pdx operon*	pACYC-Duet-1	I	NcoI, NotI
*cotB3/cotB4 operon*	pACYC-Duet-1	II	NdeI, XhoI

To evaluate if the P450 hydroxylases CotB3/4 are capable to functionalize other diterpene skeletons, the diterpene synthase gene *cotB2* in the vector pET-Duet-1 (Table 2) was exchanged by a series of other genes shown on Table 3.

**Table 3:** Plasmids used to evaluate hydroxylation of diterpenes by CotB3/4

Gene(s)	Description	Diterpene produced
*cs*	Casbene synthase	(−)−Casbene
*txs*	Taxadiene synthase	Taxa-4,11-diene
*cotB2*_*W288G*_ [13]	Cyclooctatin synthase	(1R,3E,7E,11S,12S)-3,7,18-Dolabellatriene
*cotB2*_*F107A*_ [13]	Cyclooctatin synthase	(R)- Cembrene A

### Plasmids to evaluate different redox system variants

The catalytic activities of single CotB3 and CotB4 proteins were evaluated using different redox system variants. The redox system from *S. afghaniensis* (*afR*/*afx*), *P. putida* (*pdR*/*pdx*) and mutant variants of *P. putida* (*pdR*/*pdx*) were tested. Consequently, the vector pACYC-Duet-1 was either carrying CotB3 or CotB4 expressing genes in multiple cloning site II as well as the bi-cistronic operon of reductase/ferredoxin in multiple cloning site I (Table 2). Different variants of the pACYC-Duet-1 vector are shown on Table 4.

**Table 4:** Plasmids used to compare the activity of CotB3 and CotB4 hydroxylases with different redox system variants

P450 proteins	Redox system	Mutations
CotB3	AfR/Afx	–
CotB3	PdR/Pdx	–
CotB3	PdR/Pdx	D38E
CotB3	PdR/Pdx	W106E
CotB3	PdR/Pdx	D38E, W106E
CotB4	AfR/Afx	–
CotB4	PdR/Pdx	–
CotB4	PdR/Pdx	D38E
CotB4	PdR/Pdx	W106E
CotB4	PdR/Pdx	D38E, W106E

The CotB3 expressing gene was amplified from the bi-cistronic operon (*cotB3*/*cotB4*) using the primers: 5′-ATT ACA TAT GCG TGA ACG TGG T-3′ and 5′-ATA TCT CGA GTT AAC GCG GTT CAC AAA CCA T-3′. *CotB4* was amplified from the bi-cistronic operon (*cotB3/cotB4*) using the primers: 5′-ATT ACA TAT GAA AGA TTT TTT TCG TAT GCG CAC-3′ and 5′-ATA TCT CGA GTT AAC GAG GTT CCG-3′. The bi-cistronic operons expressing (AfR/Afx)/(PdR/Pdx) and CotB3/B4 were introduced into the pACYC-Duet-1 according by standard cloning techniques. Point mutations in Pdx protein from the redox system were introduced by the QuikChange site-directed mutagenesis protocol. Primers for *pdx*_*D38E*_ were: 5′-GAT ATT GTT GGT GAA TGT GGT GGT AGC G-3′ and 5′-CGC TAC CAC CAC ATT CAC CAA CAA TAT C-3′. Primers *pdx*_*W106E*_ were: 5′-GAT GTT CCG GAT CGT CAG GAA TAA GCG GCC GCA TAA TG-3′ and 5′-CAT TAT GCG GCC GCT TAT TCC TGA CGA TCC GGA ACA TC-3′. The different pACYC-Duet-1 (*afR*/*afx* or *pdR*/*pdx*, *cotB3* or *cotB4*) vectors were transformed into Bl21 (DE3) cells.

### Production of cyclooctatin

The vectors pCola-Duet-1 (*dxr*, *dxs*), pCDF-Duet-1 (*ispD*/*ispF*, *idi*), pET-Duet-1 (*crte*, *cotB2*) and the vector pACYC-Duet-1 (*afR*/a*fx*, *cotB3/cotB4*) were introduced into *E. coli* Bl21(DE3) by standard transformation procedures. Cultivation conditions were based on Boghigian et al. [47].

### Shake-flask production culture of cyclooctatin

For cultivation in shake flasks, cells were grown in 100 or 400 mL medium containing LB medium (10 g/L tryptone, 5 g/L yeast extract, and 10 g/L NaCl) supplemented with 100 mM HEPES (pH 7.6), 40 g/L glycerol, 30 µg/mL kanamycin, 50 µg/mL streptomycin, 50 µg/mL carbenicillin, 34 µg/mL chloramphenicol, 1 mM δ-aminolevulinic acid (ALA), 1 mM FeSO_4_ × 7H_2_O, 1 mM isopropyl β-d-1-thiogalactopyranoside (IPTG) and 40 μL 30 % Antifoam (A) in 5 L baffled glass flasks. The culture was inoculated at OD_600_ 0.1 from an overnight culture (8 h cultivation 37 °C supplemented with 30 μg/mL kanamycin 50 μg/mL streptomycin, 50 μg/mL carbenicillin, 34 μg/mL chloramphenicol) and cultivated at 25 °C for 4 days.

### Batch bioprocess of cyclooctatin

A 5 L fermentation was performed in a 10 L bioreactor (Biostat C, Braun Melsungen, Germany) using LB medium (10 g/L tryptone, 5 g/L yeast extract, and 10 g/L NaCl) supplemented with 40 g/L glycerol, 30 μg/mL kanamycin, 50 μg/mL streptomycin, 50 μg/mL carbenicillin, 34 μg/mL chloramphenicol, 1 mM δ-aminolevulinic acid (ALA), 1 mM FeSO_4_ × 7H_2_O and 1 mM isopropyl β-d-1-thiogalactopyranoside (IPTG). The pH was controlled at 7.6 with 4 M NH_4_OH and 5 M H_3_PO_4_. The bioprocess was started by inoculation from an overnight culture (8 h cultivation 37 °C supplemented with 50 μg/mL kanamycin, 50 μg/mL streptomycin, 50 μg/mL carbenicillin, 34 μg/mL chloramphenicol) at OD_600_ = 0.1 and run for 4 days at 25 °C. Oxygen saturation was constantly adjusted to 80 %. To monitor the diterpene production 200 mL aliquots were taken every 24 h in triplicates. To determine the dry cell mass 10 mL aliquots were taken, centrifuged and dried. The biomass was determined gravimetrically.

### Batch bioprocess of cyclooctatin with organic overlay

A 1 L fermentation was performed in three parallel bioreactors (Labfors 5 Lux Stirred Tank, Infors HT, Switzerland) using LB medium (10 g/L tryptone, 5 g/L yeast extract, and 10 g/L NaCl) supplemented with 40 g/L glycerol, 30 μg/mL kanamycin, 50 μg/mL streptomycin, 50 μg/mL carbenicillin, 34 μg/mL chloramphenicol, 1 mM δ-aminolevulinic acid (ALA), 1 mM FeSO_4_ × 7H_2_O and 1 mM isopropyl β-d-1-thiogalactopyranoside (IPTG). The media were supplemented with 20 % (v/v) sterile dodecane. The pH was controlled at 7.6 with 4 M NH_4_OH and 5 M H_3_PO4. The bioprocess was started by inoculation from an overnight culture (8 h cultivation 37 °C supplemented with 50 μg/mL kanamycin, 50 μg/mL streptomycin, 50 μg/mL carbenicillin, 34 μg/mL chloramphenicol) at OD600 = 0.1 and run for 4 days at 25 °C. Oxygen was supplied at 0.9 (vvm) and oxygen saturation was constantly adjusted to 80 %. To monitor the diterpene production 1 mL aliquots were taken every 24 h. To determine the dry cell mass 2 mL aliquots were taken, centrifuged and dried. The biomass was determined gravimetrically.

### Production of sinularcasbane D

The vectors pCola-Duet-1 (*dxr*, *dxs*), pCDF-Duet-1 (*ispD*/*ispF*, *idi*), pET-Duet-1 (*crte*, *cs*) and the vector pACYC-Duet-1 (*afR*/*afx*, *cotB3*) were introduced into *E. coli* Bl21(DE3) by standard transformation procedures. Cultivation conditions were based on Boghigian et al. [47].

### Batch bioprocess of sinularcasbane D

For cultivation in shake flasks, cells were grown in 400 mL medium containing LB medium (10 g/L tryptone, 5 g/L yeast extract, and 10 g/L NaCl), 40 g/L glycerol, 30 μg/mL kanamycin, 50 μg/mL streptomycin, 50 μg/mL carbenicillin, 34 μg/mL chloramphenicol, 1 mM δ-aminolevulinic acid (ALA), 1 mM FeSO_4_ × 7H_2_O, 1 mM isopropyl β-d-1-thiogalactopyranoside (IPTG) and 40 μL 30 % Antifoam (A) in 5 L baffled glass flasks. The culture was inoculated at OD_600_ 0.1 from an overnight culture (8 h cultivation 37 °C supplemented with 30 μg/mL kanamycin 50 μg/mL streptomycin, 50 μg/mL carbenicillin, 34 μg/mL chloramphenicol) and cultivated at 25 °C for 3 days.

### Extraction and isolation of cyclooctatin and its biosynthetic precursors

For the diterpene isolation the cells were pelleted (15 min, 17,500 g, 4 °C). Cyclooctatin and its biosynthetic precursors were extracted from the *E. coli* cell pellet and growth medium supernatant in separate approaches. The cell pellet was washed with water and resuspended in 5 mL water. Next, cells were lysed by sonification using a Sonoplus HD2070 (Bandelin Electronic) performing five repeats on ice (5 min on and 3 min off at 80 % power) followed by a three times extraction with 25 mL ethyl acetate. The supernatant containing the growth medium was additionally extracted three times by 200 mL ethyl acetate. All organic phases were combined. After a drying step with MgSO_4_ the solvent was removed under vacuum. The crude extract was solved in 1 mL ethyl acetate and analysed by GC–MS and -FID. Purification of cyclooctat-9-en-7-ol was carried out by flash chromatography. An isocratic 70/30 hexane/ethyl acetate silica step (Silica gel 40, Sigma-Aldrich,) was followed by an isocratic 30/70 water/acetonitrile reversed phase chromatography step on a Polygoprep 60-50, C_18_ column (Macherey–Nagel). For purification of cyclooctat-9-en-5,7-diol by flash chromatography, the hexane/ethyl acetate solvent was changed to 50/50 and the water/acetonitrile solvent to 10/90. Cyclooctatin was purified by flash chromatography using hexane/ethyl acetate 30/70 as solvent followed by a 10/90 water acetonitrile reversed phase.

### Extraction and isolation of (−)−casbene and sinularcasbane D

For the (−)−casbene and sinularcasbane D isolation, cells were pelleted (15 min, 17,500 g, 4 °C). sinularcasbane D and its biosynthetic precursor (−)−casbene were extracted from *E. coli* cells and supernatant, separately. Extraction of the diterpenes was analogy to the isolation of cyclooctatin and its metabolic precursors. The crude extract was solved in 1 mL ethyl acetate and analysed by GC–MS and -FID. Purification of (−)−casbene and sinularcasbane D was carried out by flash chromatography. An isocratic 90/10 hexane/ethyl acetate chromatography on silica (Silica gel 40, Sigma-Aldrich) was applied.

### Evaluating CotB3/4 for activity on different diterpene skeletons

The vectors pCola-Duet-1 (*dxr*, *dxs*), pCDF-Duet-1 (*ispD*/*ispF*, *idi*), pET-Duet-1 (*crte*, *cs*)/pET-Duet-1 (*crte*, *txs*)/pET-Duet-1 (*crte*, *cotB2*_*W288G*_)/pET-Duet-1 (crte, *cotB2*_*F107A*_) and the vector pACYC-Duet-1 (afR/afx, cotB3)/pACYC-Duet-1 (*afR/afx*, *cotB4*) were introduced into *E. coli* Bl21 (DE3) by standard transformation procedures.

Cultivation was done in 400 mL volumes in shake flask culture. Cultivation, isolation and identification of diterpene products were analog as described in the batch bioprocess of cyclooctatin.

### Preparation and purification of MTPA derivatives

Five milligram purified cyclooctat-9-en-5,7-diol were esterified with the R- and S- corresponding Mosher acid according to literature [26]. The reaction mixtures were purified by flash chromatography using an isocratic 50/50 hexane/ethyl acetate silica phase column (Silica gel 40, Fluka Analytical). Treatment of cyclooctat-9-en-5,7-diol with (R)- and (S)-MTPA chlorides provides the (S)- and (R)-MTPA products, derived from esterification of the secondary hydroxyl group at C-5, while leaving the ternary hydroxyl group at C-20 unaffected.

### In vivo assay for comparison of redox-systems

The electron transfer of different redox system variants was evaluated to promote catalytic efficiency of CotB3 and CotB4. Therefore Bl21(DE3) cells harboring different pACYC-Duet-1 (*afR*/*afx* or *pdR*/*pdx*, *cotB3* or *cotB4*) vectors were used in an in vivo assay. In this assay the P450 and a corresponding redox system were expressed in the absence of the DXP pathway. The metabolic precursor cyclooctat-9-en-7-ol (for CotB3) or cyclooct-9-en-5,7-diol (for CotB4) was added to the medium and the amount of catalytic products cyclooct-9-en-5,7-diol (for CotB3) or cyclooctatin (for CotB4) was measured.

From an overnight culture (LB medium, 34 μg/mL chloramphenicol) Bl21(DE3) cells harboring different pACYC-Duet-1 encoding (*afR*/*afx* or *pdR*/*pdx*, *cotB3* or *cotB4*) vectors were grown in 100 mL baffled shake flask cultures at 37 °C using 50 mL LB medium supplemented with 34 μg/mL chloramphenicol. Bl21 (DE3) harboring the empty pACYC-Duet-1 vector was used as negative control. All strains were cultivated five times. After the cultures reached the OD_600_ 0.8, 1 mM δ-aminolevulinic acid (ALA), 1 mM FeSO_4_ × 7H_2_O and 1 mM isopropyl β-d-1-thiogalactopyranoside (IPTG) was added and the cultures were incubated at 30 °C for 24 h. For the in vivo assay, the cell density of the cultures was adjusted to OD_600_ 6 by centrifugation (3000*g* for 5 min) and re-suspended in the appropriate volume. 10 mL of each culture (OD_600_ = 6) were transferred to 50 mL baffled shake flasks and supplemented with 100 μM of cyclooctat-9-en-7-ol (CotB3) or cyclooct-9-en-5,7-diol (CotB4). Terpenes were added in acetonitrile to a final concentration of 0.1 % (*v/v*). The cultures were sealed by membranes (Greiner bio-one breath seal 676051) to avoid evaporation and incubated at 30 °C for 2 days. To analyze the whole cell proteins 100 μL samples were taken every 6 h. The culture samples were centrifuged (10,000*g* for 5 min), the supernatant was discarded and the cell pellets were stored at −20 °C. The samples were analyzed by a 12 % SDS-Page gel according to Laemmli. After cultivation the cultures were directly lysed by sonification on a Sonoplus HD2070 (Bandelin Electronic, Germany) performing five repeats on ice (5 min on and 3 min off at 80 % power) and subsequently extracted three times with 25 mL ethyl acetate. All organic phases were combined. After a drying step with MgSO_4_ the solvent was removed under vacuum. The crude extract was solved in 1 mL ethyl acetate and analyzed by GC–MS. The GC–MS spectra were normalized to the total icon count of α-humulene (Sigma-Aldrich, Germany), which was used as an internal standard. Quantification was done by determining the total ion count of cyclooctatin and cyclooct-9-en-5,7-diol, respectively.

### Bioinformatics

#### Modelling of CotB3 and CotB4

Homology models were built with Schrödinger’s Prime [Prime, version 3.4 (Schrödinger, LLC, New York)] from alignments derived from PROMALS3D [48]. For CotB3, we applied a chimera model using the crystal structure of *Streptomyces coelicolor* cytochrome P450 (PDB 3EL3, 39 % sequence identity) as main template and crystal structure of human cytochrome P450 (PDB 3LD6, 23 % sequence identity) as template for missing loops at positions 194–209 and 250–258. We applied the same approach for CotB4, with sequence identities of 39 and 20 % for templates 3EL3 and 3LD6, respectively. Resulting models were subject to protonation state assignment at pH 7.0 using PROPKA and protein relaxation using restrained minimization at a convergence factor of RMSD = 0.3 Å for the heavy atoms. The prosthetic heme group was kept frozen during this process and the formal charge of its iron atom set to +3 (high-spin sexted Fe(III)), as we simulated the enzyme-substrate complex right after the resting state where the substrate has replaced the distal water.

#### Docking of CotB3, CotB4 with its substrates

The ligand structures of cyclooct-9-en-7-ol and cyclooct-9-en-5,7-diol were built with Schrödinger’s Maestro [Maestro version 0.6 (Schrödinger, LLC, New York)]. Their conformations and partial charges were taken from QM minimization with a dielectric constant of 4.8, simulating protein environment. Ligand docking was done with Schrödinger’s induced fit docking protocol. First, the receptor residues within 5.0 Å of the ligand are trimmed, then the ligand flexibly docked into the cavity and finally the side chains of residues within 5.0 Å vicinity are sampled and minimized together with the ligand. The prothetic heme group was kept frozen throughout this process and resulting docking poses were sorted by the IFDScore.

#### Modelling of complexes

Secondary structure analysis and modeling of the tertiary structures of putative reductases and ferredoxins from *S. afghaniensis* whole shotgun-sequences was done using the HHpred server tool [23]. Structural alignments have been performed using the MUSTANG approach of YASARA structure [49]. The model of the AfR·Afx complex has been derived by subsequent alignments of both AfR and Afx against the PdR·Pdx (PDB-ID·3LB8) complex. Models of CotB3·/CotB4·Afx complexes have been derived by the same approach using the P450cam·Pdx complex (PDB-ID·4JX1) as template.

Assigned complexes have subsequently been subjected to 10,000 steps of energy minimization in a TIP3P water box at pH 7.0 using the YAMBER03 force field of YASARA structure. The force field parameters of cyclooct-9-en-7-ol and cyclooct-9-en-5,7-diol, the prosthetic heme group and the Fe_2_/S_2_-cluster was obtained using the AutoSmiles force field parameter assignment implemented in YASARA structure [50–52]. During energy minimization all residues, heavy atoms, prosthetic groups and both substrates were unconstrained, non-bonded cutoff were set to 7.5 Å and PME method (Particle-Mesh-Ewald) was used for long-range electrostatic forces.

#### Molecular docking of (−)−casbene and sinularcasbane D in the cotB3 active site

Molecular Docking was performed using the AutoDock Vina program environment of YASARA structure. Sinularcasbane D was docked into a simulation cell (Size: X-size = 16 Å, Y-size = 16 Å, Z-size = 16 Å, angles: alpha = 90°, beta = 90°, gamma = 90°) around the following four residues Cys87, Arg185, Ala282 and Leu361. 999 docking runs were performed while all atoms of (−)−casbene and sinularcasbane D were set as rigid. Cluster analysis were performed in the AutoDock Vina program environment and characterized by binding energy [kcal/mol], dissociation constant [pM] and contacting receptor residues.

#### Quality assessments

For quality assessments the following tools were used.

Stereochemical quality assessments: PROCHECK software package (Ramachandran plot, g-factor) [53, 54].

Assessment of absolute structural model qualities by QMEAN Z-scores [55–58].

## Additional file

10.1186/s12934-016-0487-6 Additional data corresponding to the in vivo assay, bioinformatics, NMR spectra, CD, MS analytics, genes can be found in the supporting information.

## References

[1] Chang MC, Keasling JD (2006). Production of isoprenoid pharmaceuticals by engineered microbes. Nat Chem Biol.

[2] Keasling JD (2012). Synthetic biology and the development of tools for metabolic engineering. Metab Eng.

[3] Brück T, Kourist R, Loll B (2014). Production of macrocyclic sesqui-and diterpenes in heterologous microbial hosts: a systems approach to harness nature’s molecular diversity. ChemCatChem.

[4] Urlacher VB, Girhard M (2012). Cytochrome P450 monooxygenases: an update on perspectives for synthetic application. Trends Biotechnol.

[5] Le-Huu P, Heidt T, Claasen B, Laschat S, Urlacher VB (2015). Chemo-, regio-, and stereoselective oxidation of the monocyclic diterpenoid β-cembrenediol by P450 BM3. ACS Catal.

[6] Ajikumar PK, Xiao W-H, Tyo KE, Wang Y, Simeon F, Leonard E, Mucha O, Phon TH, Pfeifer B, Stephanopoulos G (2010). Isoprenoid pathway optimization for Taxol precursor overproduction in *Escherichia coli*. Science.

[7] Paddon C, Westfall P, Pitera D, Benjamin K, Fisher K, McPhee D, Leavell M, Tai A, Main A, Eng D, Polichuk DR, Teoh KH, Reed DW, Treynor T, Lenihan J, Fleck M, Bajad S, Dang G, Dengrove D, Diola D, Dorin G, Ellens KW, Fickes S, Galazzo J, Gaucher SP, Geistlinger T, Henry R, Hepp M, Horning T, Iqbal T, Jiang H, Kizer L, Lieu B, Melis D, Moss N, Regentin R, Secrest S, Tsuruta H, Vazquez R, Westblade LF, Xu L, Yu M, Zhang Y, Zhao L, Lievense J, Covello PS, Keasling JD, Reiling KK, Renninger NS, Newman JD (2013). High-level semi-synthetic production of the potent antimalarial artemisinin. Nature.

[8] Bleif S, Hannemann F, Lisurek M, von Kries JP, Zapp J, Dietzen M, Antes I, Bernhardt R (2011). Identification of CYP106A2 as a regioselective allylic bacterial diterpene hydroxylase. ChemBioChem.

[9] Torres Pazmiño DEW, Winkler M, Glieder A, Fraaije MW (2010). Monooxygenases as biocatalysts: classification, mechanistic aspects and biotechnological applications. J Biotechnol.

[10] Chang MC, Eachus RA, Trieu W, Ro D-K, Keasling JD (2007). Engineering *Escherichia coli* for production of functionalized terpenoids using plant P450s. Nat Chem Biol.

[11] Yadav VG (2014). Unraveling the multispecificity and catalytic promiscuity of taxadiene monooxygenase. J Mol Catal B Enzym.

[12] Jung ST, Lauchli R, Arnold FH (2011). Cytochrome P450: taming a wild type enzyme. Curr Opin Biotechnol.

[13] Görner C, Hirte M, Huber S, Schrepfer P, Brück T (2015). Stereoselective chemo-enzymatic oxidation routes for (1R, 3E, 7E, 11S, 12S)-3, 7, 18-dolabellatriene. Front Microbiol.

[14] Tripathi S, Li H, Poulos TL (2013). Structural basis for effector control and redox partner recognition in cytochrome P450. Science.

[15] Yang W, Bell SG, Wang H, Zhou W, Hoskins N, Dale A, Bartlam M, Wong L-L, Rao Z (2010). Molecular characterization of a class I P450 electron transfer system from *Novosphingobium aromaticivorans* DSM12444. J Biol Chem.

[16] Bhattarai S, Liou K, Oh T-J (2013). Hydroxylation of long chain fatty acids by CYP147F1, a new cytochrome P450 subfamily protein from *Streptomyces peucetius*. Arch Biochem Biophys.

[17] Girhard M, Klaus T, Khatri Y, Bernhardt R, Urlacher VB (2010). Characterization of the versatile monooxygenase CYP109B1 from *Bacillus subtilis*. Appl Microbiol Biotechnol.

[18] Pandey BP, Lee N, Choi K-Y, Jung E, Jeong D-h, Kim B-G (2011). Screening of bacterial cytochrome P450s responsible for regiospecific hydroxylation of (iso) flavonoids. Enzym Microb Technol.

[19] Kim S-Y, Zhao P, Igarashi M, Sawa R, Tomita T, Nishiyama M, Kuzuyama T (2009). Cloning and heterologous expression of the cyclooctatin biosynthetic gene cluster afford a diterpene cyclase and two p450 hydroxylases. Chem Biol.

[20] Yasutake Y, Nishioka T, Imoto N, Tamura T (2013). A single mutation at the ferredoxin binding site of P450 Vdh enables efficient biocatalytic production of 25-hydroxyvitamin D3. ChemBioChem.

[21] Bell SG, McMillan JH, Yorke JA, Kavanagh E, Johnson EO, Wong L-L (2012). Tailoring an alien ferredoxin to support native-like P450 monooxygenase activity. Chem Commun.

[22] Peterson J, Lorence M, Amarneh B (1990). Putidaredoxin reductase and putidaredoxin. Cloning, sequence determination, and heterologous expression of the proteins. J Biol Chem.

[23] Söding J, Biegert A, Lupas AN (2005). The HHpred interactive server for protein homology detection and structure prediction. Nucleic Acids Res.

[24] Peterson J, Lu J-Y, Geisselsoder J, Graham-Lorence S, Carmona C, Witney F, Lorence M (1992). Cytochrome P-450terp. Isolation and purification of the protein and cloning and sequencing of its operon. J Biol Chem.

[25] Meguro A, Motoyoshi Y, Teramoto K, Ueda S, Totsuka Y, Ando Y, Tomita T, Kim SY, Kimura T, Igarashi M (2015). An unusual terpene cyclization mechanism involving a carbon-carbon bond rearrangement. Angew Chem.

[26] Ohtani I, Kusumi T, Kashman Y, Kakisawa H (1991). High-field FT NMR application of Mosher’s method. The absolute configurations of marine terpenoids. J Am Chem Soc.

[27] Komatsu N, Nakazawa S, Hamada M, Shimo M, Tomosugi T (1959). Studies on taitomycin, a new antibiotic produced by *Streptomyces*, sp. No. 772 (*S. afghaniensis*). III. Biological studies on taitomycin. J Antibiot.

[28] Parenti F, Coronelli C (1979). Members of the genus actinoplanes and their antibiotics. Annu Rev Microbiol.

[29] Citron CA, Barra L, Wink J, Dickschat JS (2015). Volatiles from nineteen recently genome sequenced actinomycetes. Org Biomol Chem.

[30] Makino T, Katsuyama Y, Otomatsu T, Misawa N, Ohnishi Y (2014). Regio-and stereospecific hydroxylation of various steroids at the 16α position of the D ring by the *Streptomyces**griseus* cytochrome P450 CYP154C3. Appl Environ Microbiol.

[31] Hussain HA, Ward JM (2003). Enhanced heterologous expression of two *Streptomyces**griseolus* cytochrome P450s and *Streptomyces**coelicolor* ferredoxin reductase as potentially efficient hydroxylation catalysts. Appl Environ Microbiol.

[32] Pandey BP, Roh C, Choi KY, Lee N, Kim EJ, Ko S, Kim T, Yun H, Kim BG (2010). Regioselective hydroxylation of daidzein using P450 (CYP105D7) from *Streptomyces avermitilis* MA4680. Biotechnol Bioeng.

[33] Strushkevich N, MacKenzie F, Cherkesova T, Grabovec I, Usanov S, Park H-W (2011). Structural basis for pregnenolone biosynthesis by the mitochondrial monooxygenase system. Proc Natl Acad Sci USA.

[34] Hiruma Y, Hass MA, Kikui Y, Liu W-M, Ölmez B, Skinner SP, Blok A, Kloosterman A, Koteishi H, Löhr F (2013). The structure of the cytochrome P450cam–putidaredoxin complex determined by paramagnetic NMR spectroscopy and crystallography. J Mol Biol.

[35] Madrona Y, Hollingsworth SA, Tripathi S, Fields JB, Rwigema J-CN, Tobias DJ, Poulos TL (2014). Crystal structure of cindoxin, the P450cin redox partner. Biochemistry.

[36] Sevrioukova IF, Poulos TL (2011). Structural biology of redox partner interactions in P450cam monooxygenase: a fresh look at an old system. Arch Biochem Biophys.

[37] Kuznetsov VY, Poulos TL, Sevrioukova IF (2006). Putidaredoxin-to-cytochrome P450cam electron transfer: differences between the two reductive steps required for catalysis. Biochemistry.

[38] Wallrapp F, Masone D, Guallar V (2008). Electron transfer in the P450cam/PDX complex. The QM/MM e-pathway. J Phys Chem A.

[39] Janke R, Görner C, Hirte M, Brueck T, Loll B (2014). The first structure of a bacterial diterpene cyclase: CotB2. Acta Crystallogr D Biol Crystallogr.

[40] Nakano Y, Ohtani M, Polsri W, Usami T, Sambongi K, Demura T (2012). Characterization of the casbene synthase homolog from Jatropha (*Jatropha curcas* L.). Plant Biotechnol.

[41] Wildung MR, Croteau R (1996). A cDNA clone for taxadiene synthase, the diterpene cyclase that catalyzes the committed step of taxol biosynthesis. J Biol Chem.

[42] Yin J, Zhao M, Ma M, Xu Y, Xiang Z, Cai Y, Dong J, Lei X, Huang K, Yan P (2013). New casbane diterpenoids from a South China Sea soft coral *Sinularia* sp.. Mar Drugs.

[43] Franceschini S, van Beek HL, Pennetta A, Martinoli C, Fraaije MW, Mattevi A (2012). Exploring the structural basis of substrate preferences in Baeyer–Villiger monooxygenases insight from steroid monooxygenase. J Biol Chem.

[44] Dietrich J, Yoshikuni Y, Fisher K, Woolard F, Ockey D, McPhee D, Renninger N, Chang M, Baker D (2009). JD 10 Keasling. ACS Chem Biol.

[45] Costin CD, Hansen SL, Chambers DP (2009). Using theoretical correction factors for quantitative analysis of sterols and sterol concentrates. J Am Oil Chem Soc.

[46] Schäfer H, Nau K, Sickmann A, Erdmann R, Meyer HE (2001). Identification of peroxisomal membrane proteins of Saccharomyces cerevisiae by mass spectrometry. Electrophoresis.

[47] Boghigian BA, Myint M, Wu J, Pfeifer BA (2011). Simultaneous production and partitioning of heterologous polyketide and isoprenoid natural products in an *Escherichia coli* two-phase bioprocess. J Ind Microbiol Biotechnol.

[48] Pei J, Kim B-H, Grishin NV (2008). PROMALS3D: a tool for multiple protein sequence and structure alignments. Nucleic Acids Res.

[49] Konagurthu AS, Whisstock JC, Stuckey PJ, Lesk AM (2006). MUSTANG: a multiple structural alignment algorithm. Proteins.

[50] Jakalian A, Jack DB, Bayly CI (2002). Fast, efficient generation of high-quality atomic charges. AM1-BCC model: II. Parameterization and validation. J Comput Chem.

[51] Wang J, Wolf RM, Caldwell JW, Kollman PA, Case DA (2004). Development and testing of a general amber force field. J Comput Chem.

[52] Stewart JJ (1990). MOPAC: a semiempirical molecular orbital program. J Comput Aided Mol Des.

[53] Laskowski RA, MacArthur MW, Moss DS, Thornton JM (1993). PROCHECK: a program to check the stereochemical quality of protein structures. J Appl Crystallogr.

[54] Engh RA, Huber R (1991). Accurate bond and angle parameters for X-ray protein structure refinement. Acta Crystallogr A.

[55] Benkert P, Biasini M, Schwede T (2011). Toward the estimation of the absolute quality of individual protein structure models. Bioinformatics.

[56] Baker D, Sali A (2001). Protein structure prediction and structural genomics. Science.

[57] Benkert P, Tosatto SC, Schomburg D (2008). QMEAN: a comprehensive scoring function for model quality assessment. Proteins.

[58] Benkert P, Schwede T, Tosatto SC (2009). QMEANclust: estimation of protein model quality by combining a composite scoring function with structural density information. BMC Struct Biol.

