# Supplementary material for: Identification, characterization and molecular adaptation of class I redox systems for the production of hydroxylated diterpenoids
Source: Microb Cell Fact. 2016 May 23;15:86. doi: 10.1186/s12934-016-0487-6 (PMC4877809; doi:10.1186/s12934-016-0487-6)
Supplement: Supplementary file 1 — 10.1186/s12934-016-0487-6 Additional data corresponding to the in vivo assay, bioinformatics, NMR spectra, CD, MS analytics, genes can be found in the supporting information. [file 12934_2016_487_MOESM1_ESM.pdf]

Supporting Information

Identification, characterization and molecular adaptation of class I redox systems for the production of multi-functionalized diterpenoids

Christian Görner[a, ‡], Patrick Schrepfer[a, ‡], Veronika Redai[a], Frank Wallrapp[b], Bernhard Loll[c], Wolfgang Eisenreich[e], Martin Haslbeck[f] and Thomas Brück[a, *]

[a]Division of Industrial Biocatalysis, Technical University of Munich, Lichtenberg Str. 4, 85748 Garching, Germany

[b]Chair of Bioinformatics, Technical University of Munich, Boltzmannstraße 3, 85748 Garching, Germany

[c]Institute for Chemistry and Biochemistry, Division of Structural Biology, Freie Universität Berlin, Takustr. 6, 14195 Berlin, Germany

[d]Chair of Biochemistry, Technical University of Munich, Lichtenberg Str. 4, 85748 Garching

[e]Chair of Biotechnology, Technical University of Munich, Lichtenbergstr. 4, 85748 Garching, Germany

‡These authors contributed equally.

* To whom correspondence should be addressed:

Thomas Brück

Fachgebiet für Industrielle Biokatalyse

Zentrum für Weiße Biotechnologie

Department für Chemie

Technische Universität München

Lichtenberg Str. 4, 85748 Garching, Germany

E-mail: brueck@tum.de

Content

1. *In vivo* assay 7

Figure S1a. *In vivo* assay for the conversion of cyclooctat-9-en-7-ol by CotB3 to cyclooctat-9-en-5,7-diol using different redox system variants. The data is illustrated as boxplot diagram (25 % to 75 % with whiskers with maximum 1.5 IQR) from five whole cell hydroxylation experiments. 100 % yield correspond to the median of the CotB3/AfR/Afx control 7

Figure S1b. *In vivo* assay for the conversion of cyclooctat-9-en-5,7-diol by CotB4 to cyclooctatin using different redox system variants. The data is illustrated as boxplot diagram (25 % to 75 % with whiskers with maximum 1.5 IQR) from five whole cell hydroxylation experiments. 100 % yield correspond to the median of the CotB3/AfR/Afx control. 7

Figure S1c. SDS Page of the whole cell proteome from the *in vivo* assay (24 hours). The catalytic efficiency of CotB3 was evaluated using different redox system variants. 8

Table S1. Description of Figure S1c.. 8

Figure S1d. SDS Page of the whole cell proteome from the *in vivo* assay (24 hours). The catalytic efficiency of CotB4 was evaluated using different redox system variants. 9

Table S2. Description of Figure S1d 9

2. Bioinformatics 10

Figure S2a. Model of CotB3 harboring cyclooct-9-en-7-ol. Modelled CotB3 (gray), prosthetic heme group (magenta) and cyclooct-9-en-7-ol (blue).. 10

Figure S2b. Structural alignment of CotB3 (cyan) and P450cam (PDB-ID: 4JX1, red). The alignment has an RMSD of 0.142Å over 313 aligned residues with 19% sequence identity. Note that the RMSD value considers only atoms that are present in both structures. Not matching loops in CotB3 are not incorporated into the alignment. The interaction region of CotB3 with Afx/Pdx is part of the matched helical structures. 11

Figure S2c. Model of CotB4 harboring cyclooct-9-en-5,7-diol. Modelled CotB4 (gray), prosthetic heme group (magenta) and cyclooct-9-en-5,7-diol (blue). 12

Fig S2d. Structural alignment of CotB4 (cyan) and P450cam (PDB-ID: 4JX1, red). The alignment has an RMSD of 0.144Å over 339 aligned residues with 18% sequence identity. Note that the RMSD value considers only atoms that are present in both structures. Not matching loops in CotB4 are not incorporated into the alignment. The interaction region of CotB4 with Afx/Pdx is part of the matched helical structures. 13

Figure S2e. Modelled AfR•Afx complex with AfR harboring FAD (Flavin-Adenine-Dinucleotide) and Afx harboring inorganic Fe_2/S_2-cluster. AfR•Afx complex (dark blue/gray). AfR with bound prosthetic FAD (magenta) and Afx bound to inorganic Fe_2/S_2-cluster (light blue). W297 of AfR proposed to be acting in electron transfer is shown in red.. 14

Figure S2f. Modelled CotB3•Afx complex harboring cyclooct-9-en-7-ol. CotB3•Afx complex (dark blue/orange). CotB3 contains prosthetic heme group (magenta) and cyclooct-9-en-7-ol (light blue). Afx bound to inorganic Fe_2/S_2-cluster (green/magenta). 15

Figure S2g. Modelled CotB4•Afx complex harboring cyclooct-9-en-7-ol. CotB4•Afx complex (dark blue/orange). CotB4 contains prosthetic heme group (magenta) and cyclooct-9-en-5,7-diol (light blue). Afx contains inorganic Fe2/S2-cluster (green/magenta).................................. 16

Figure S2h. Inner view of the Cotb3•Pdx complex (dark blue•red). Pdx contains inorganic Fe2/S2-cluster (green/magenta). CotB3 contains prosthetic heme group (gray) and cyclooct-9-en-7-ol (red). Essential hydrogen bonds for the interaction of Pdx and CotB3 to facilitate binding and electron transport are highlighted in black. Hydrogen bond formation between $CotB3_{R110}$ and $CotB3_{R114}$ with Pdx_{W106} needed for efficient binding and electron transfer, is strongly impaired due to the chemical nature of participating amino acids. The Cotb4•Pdx complex exhibits the same structure and also the same interacting residues. 17

Figure S2i. Inner view of the Cotb4•Afx complex (dark blue•red). Afx contains inorganic Fe_2/S_2-cluster (green/magenta). CotB4 contains prosthetic heme group (gray) and cyclooct-9-en-5,7-diol (red). Essential hydrogen bonds for the interaction of Afx and CotB4 to facilitate binding and electron transport are highlighted in black. 18

Figure S2j. Structural superposition of the PdR•Pdx (red·blue) with the modelled AfR•Afx complex (ocher•gray). Prosthetic FAD group of PdR and modelled AfR are shown in light blue. 2Fe·2S cluster of Pdx and modelled Afx are shown in magenta/green. The important hydrogen bridge (black) between Pdx_{D38} and PdR_{R310} needed for binding as well as PdR_{W330} facilitating electron transport are shown. Differing position of PdR_{W330} corresponding to W297 in AfR, presumably acting in electron transfer from FAD to Pdx is shown in ocher. Analogous interactions facilitating binding of AfR to Afx could not be deciphered. Substitution of Pdx_{D38} to E38 is suggested to impair electron transport by an alternate binding mode of Pdx_{D38E} to native PdR. 19

Figure S2k. Modelled CotB3 harboring (-)-casbene. CotB3 (light purple) contains prosthetic heme group (blue) and docked (-)-casbene (gray). The heme group contains the iron cation (magenta) and the dative bond to cysteine 408 (green) of the CotB3. (-)-Casbene is shown in the conformation comprising the lowest binding energy derived by cluster analysis of AutodockVina. Hydroxylated C-10 of (-)-casbene is shown in blue. 20

Figure S2l. Modelled CotB3 harboring sinularcasbene D. CotB3 (light purple) contains prosthetic heme group (blue) and docked sinularcasbene D (gray). The heme group contains the iron cation (magenta) and the dative bond to cysteine 408 (green) of the CotB3. Sinularcasbene D is shown in the conformation comprising the lowest binding energy derived by cluster analysis of AutodockVina. 21

Table S3. PROCHECK results of CotB3/4, AfR and Afx. 22

QMEAN Z-scores 22

Figure S2m. QMEAN Z-score of the AfR model (-0.31) 22

Figure S2n. QMEAN Z-score of the Afx model (-0.10) 23

Figure S2o. QMEAN Z-score of the CotB3 model (-1.15) 23

Figure S2p. QMEAN Z-score of the CotB4 model (-1.33) 24

3. NMR-Spectroscopy 25

Figure S3a. 1H NMR (500 MHz, CDCl3) of cyclooctat-9-en-7-ol 25

Figure S3b. ^{13}C NMR (125 MHz, $CDCl_3$) of cyclooctat-9-en-7-ol 26

Figure S3c. ^{1}H NMR (500 MHz, $CDCl_3$) of cyclooctat-9-en-5,7-diol 27

Figure S3d. ^{13}C NMR (125 MHz, $CDCl_3$) of cyclooctat-9-en-5,7-diol 28

Figure S3e. ^{1}H NMR (500 MHz, CD_3-OD) of cyclooctatin 29

Figure S3f. ^{13}C NMR (125 MHz, CD_3-OD) of cyclooctatin 30

Figure S3g. COSY (500 MHz, CD_3-OD) NMR of cyclooctatin 31

Figure S3h. HSQC (500 MHz, CD_3-OD) NMR of cyclooctatin 32

Figure S3i. NOESY NMR (500 MHz, CD3-OD) of cyclooctatin 33

Figure S3j ^{1}H NMR (500 MHz, $CDCl_3$) of the C-5 R-MTPA derivative of cyclooctat-9-en-5,7-diol. 34

Figure S3k. ^{13}C NMR (125 MHz, $CDCl_3$) of the C-5 R-MTPA derivative of cyclooctat-9-en-5,7-diol 35

Figure S3l. COSY NMR (500 MHz, $CDCl_3$) of the C-5 R-MTPA derivative of cyclooctat-9-en-5,7-diol 36

Figure S3m. HSQC NMR (500 MHz, $CDCl_3$) of the C-5 R-MTPA derivative of cyclooctat-9-en-5,7-diol 37

Figure S3n. NOESY NMR (500 MHz, $CDCl_3$) of the C-5 R-MTPA derivative of cyclooctat-9-en-5,7-diol 38

Figure S3o. ^{1}H NMR (500 MHz, $CDCl_3$) of the C-5 S-MTPA derivative of cyclooctat-9-en-5,7-diol 39

Figure S3p. ^{13}C NMR (125 MHz, $CDCl_3$) of the C-5 S-MTPA derivative of cyclooctat-9-en-5,7-diol 40

Figure S3q. COSY NMR (500 MHz, $CDCl_3$) of the C-5 S-MTPA derivative of cyclooctat-9-en-5,7-diol 41

Figure S3r. HSQC NMR (500 MHz, $CDCl_3$) of the C-5 S-MTPA derivative of cyclooctat-9-en-5,7-diol 42

Figure S3s. NOESY NMR (500 MHz, $CDCl_3$) of the C-5 S-MTPA derivative of cyclooctat-9-en-5,7-diol 43

Chiral derivatizing and analysis 44

Figure S3t. ^{1}H NMR (500 MHz, $CDCl_3$) Overlay of the C-5 R/S-MTPA derivatives of cyclooctat-9-en-5,7-diol 44

Table S4. Experimental ^{1}H ($CDCl_3$, 500 MHz, δ_H) and ^{13}C ($CDCl_3$, 125 MHz, δ_C) NMR data of the C-5 R-MTPA derivatives of cyclooctat-9-en-5,7-diol 45

Table S5. Experimental ^{1}H ($CDCl_3$, 500 MHz, δ_H) and ^{13}C ($CDCl_3$, 125 MHz, δ_C) NMR data of the C-5 S-MTPA derivatives of cyclooctat-9-en-5,7-diol 46

Figure S4. Δδ (S – R) values (ppm) for the C-5 MTPA derivatives of cyclooctat-9-en-5,7-diol 47

Figure S5a. ^{1}H NMR (500 MHz, $CDCl_3$) of (-)-Casbene 48

Figure S5b. ^{13}C NMR (125 MHz, $CDCl_3$) of (-)-Casbene 49

Table S6. Experimental ^{1}H ($CDCl_3$, 500 MHz, δ_H, J in Hz) and ^{13}C ($CDCl_3$, 125 MHz, δ_C) NMR data of sinularcasbane D 50

Figure S6. COSY and HMBC correlations of sinularcasbane D 51

Figure S7. Illustrated NOESY correlations of sinularcasbane D. Modelling was done using molecular mechanics (MM2) calculations with ChemDraw Ultra Ver. 13.0 (CambridgeSoft). 51

Figure S8a. ^{1}H NMR (500 MHz, $CDCl_3$) of sinularcasbane D 52

Figure S8b. ^{1}H NMR (500 MHz, $CDCl_3$) of sinularcasbane D 53

Figure S8c. ^{13}C NMR (125 MHz, $CDCl_3$) of sinularcasbane D 54

Figure S8d. COSY NMR (500 MHz, $CDCl_3$) of sinularcasbane D 55

Figure S8e. HSQC NMR (500 MHz, $CDCl_3$) of sinularcasbane D 56

Figure S8f. HMBC NMR (500 MHz, $CDCl_3$) of sinularcasbane D 57

Figure S8g. NOESY NMR (500 MHz, $CDCl_3$) of sinularcasbane D 58

4. CD-Spectroscopy 59

Figure S9. Circular dichroism spectroscopy of cyclooctat-9-en-7-ol (**a**), cyclooctat-9-en-5-7-diol (**b**), cyclooctatin (**c**) 59

5. Mass spectrometry 60

Figure S10a. Mass spectrum of silylated cyclooctat-9-en-7-ol (RT: 21:16), recorded on a Trace GC Ultra with DSQII (Thermo Scientific), m/z was analyzed from [50-650]. m/z $C_{23}H_{42}OSi$ calculated. 362.30. 60

Figure S10b. Mass spectrum of silylated cyclooctat-9-en-5,7-diol (RT: 22:94), recorded on a Trace GC Ultra with DSQII (Thermo Scientific), m/z was analyzed from [50-650]. *m/z* $C_{26}H_{50}O_2Si_2$ calculated 450.33. 60

Figure S10c. Mass spectrum of silylated cyclooctatin (RT: 24:40), recorded on a Trace GC Ultra with DSQII (Thermo Scientific), m/z was analyzed from [50-650]. *m/z* $C_{28}H_{56}O_3Si_3$ calculated 524.35. 61

Figure S10f. Mass spectrum of sinularcasbane D (RT: 21:89), recorded on a Trace GC Ultra with DSQII (Thermo Scientific), m/z was analyzed from [50-650]. *m/z* $C_{20}H_{32}O$ calculated 288.24. 61

Figure S11. High resolution electron spray ionization of cyclooctat-9-en-7-ol (**a**) *m/z* ($[M–H_2O]^+$ 272.2568 calculated $C_{20}H_{32}$ 272.2504, cyclooctat-9-en-5-7-diol (**b**) *m/z* ($[M–H_2O]^+$ 272.2450 calculated $C_{20}H_{32}O$ 288.2453, cyclooctatin (**c**) *m/z* ($[M–H_2O]^+$ 304.2415 calculated $C_{20}H_{32}O_2$ 304.2402. 62

Figure S12. GC-MS spectra from cell pellet extract of a 72h shake flask culture of (-)-casbene and hydroxylation to sinularcasbene D by CotB3 on a Trace GC Ultra with DSQII (Thermo Scientific). 63

6. Genes 64

Figure S13. Gene of 1-deoxy-D-xylulose 5-phosphate synthase (dxs) 64

Figure S14. Gene of 1-deoxy-D-xylulose 5-phosphate reductoisomerase (*dxp*) 65

Figure S15. Bi-cistronic operon of 2-C-methyl-D-erythriol 4-phosphate cytidyltransferase synthase (*ispD*) 2-C-methyl-D-erythritol 2,4-cyclodiphosphate synthase (*ispF*) 66

Figure S16. Gene of Isopentenyl-diphosphate delta isomerase (*Idi*) 66

Figure S17. Gene of *cotB2* 67

Figure S18. Bi-cistronic *cotB3*/*cotB4* operon 68

Figure S19. Bi-cistronic *afR*/*afx* operon 69

Figure S20. Bi-cistronic *pdR*/*pdx* operon 70

Figure S21.(-)-casbene synthase (*cs*) from *Jatropha curcas* 71

Figure S22. Taxadiene synthase (*txs*) from *Taxus brevifolia* 72

1. *In vivo* assay

Figure S1a. *In vivo* assay for the conversion of cyclooctat-9-en-7-ol by CotB3 to cyclooctat-9-en-5,7-diol using different redox system variants. The data is illustrated as boxplot diagram (25% to 75% with whiskers with maximum 1.5 IQR) from five whole cell hydroxylation experiments. 100% yield correspond to the median of the CotB3/AfR/Afx control.

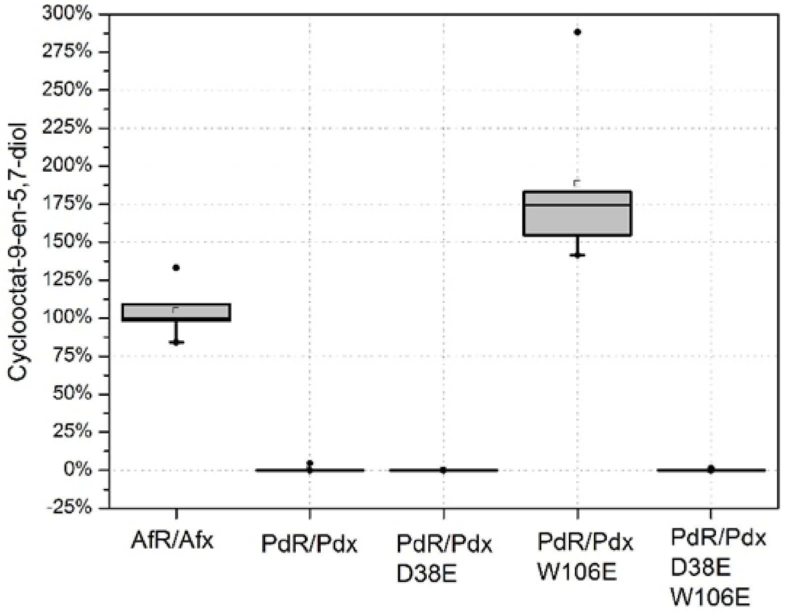

Figure S1b. *In vivo* assay for the conversion of cyclooctat-9-en-5,7-diol by CotB4 to cyclooctatin using different redox system variants. The data is illustrated as boxplot diagram (25% to 75% with whiskers with maximum 1.5 IQR) from five whole cell hydroxylation experiments. 100% yield correspond to the median of the CotB3/AfR/Afx control.

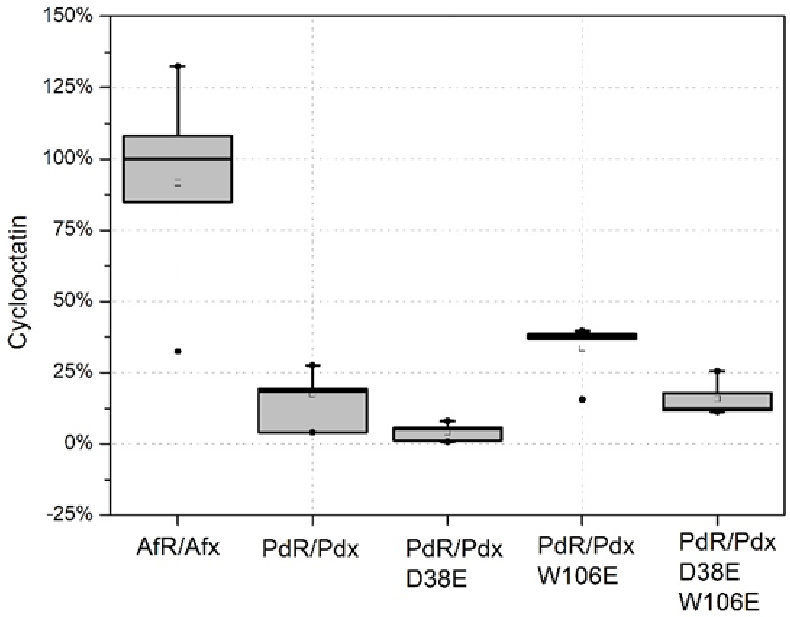

Figure S1c. SDS-Page of the whole cell proteome from the *in vivo* assay (24 hours). The catalytic efficiency of CotB3 was evaluated using different redox system variants.

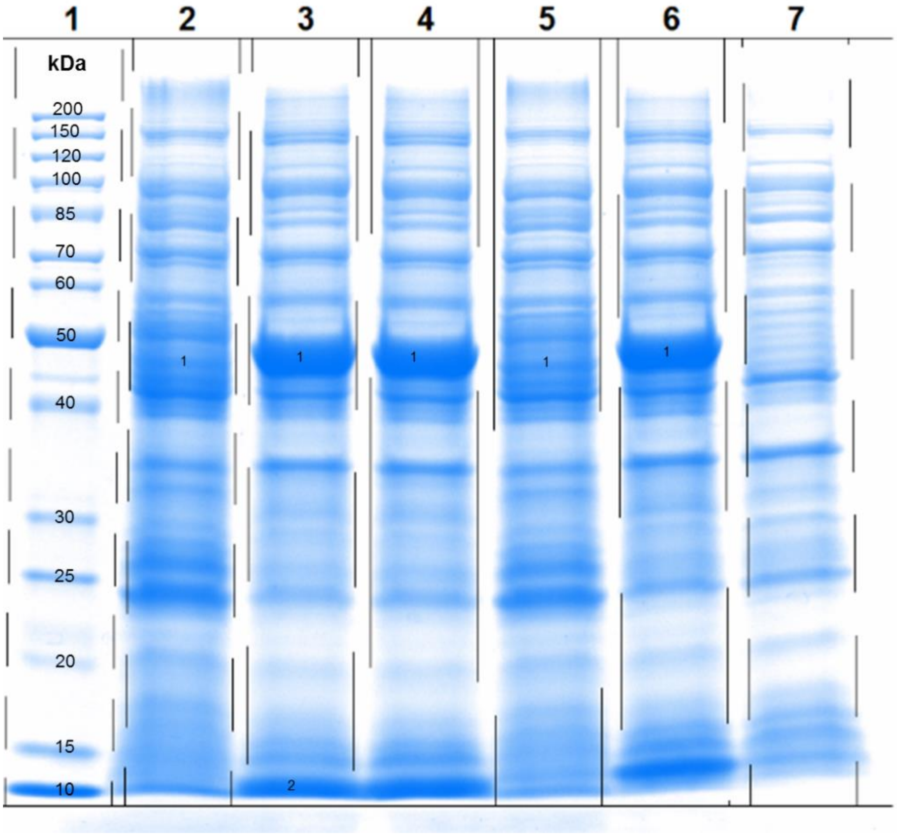

Table S1. Description of Figure S1c

Lane #	*Lane Description*	*Protein Band #*	*Protein Identified*
1	Marker	1	CotB3
2	pACYC-Duet-1 (*afR*/*afx*, cotB3)	2	Pdx
3	pACYC-Duet-1 (*pdR*/*pdx*, cotB3)		
4	pACYC-Duet-1 (*pdR*/*pdx*-D38E, cotB3)		
5	pACYC-Duet-1 (*pdR*/*pdx*-D38E, cotB3)		
6	pACYC-Duet-1 (pdR/pdx-D38E/W106E, cotB3)		
7	pACYC-Duet-1 (empty)		

Figure S1d. SDS-Page of the whole cell proteome from the *in vivo* assay (24 hours). The catalytic efficiency of CotB4 was evaluated using different redox system variants.

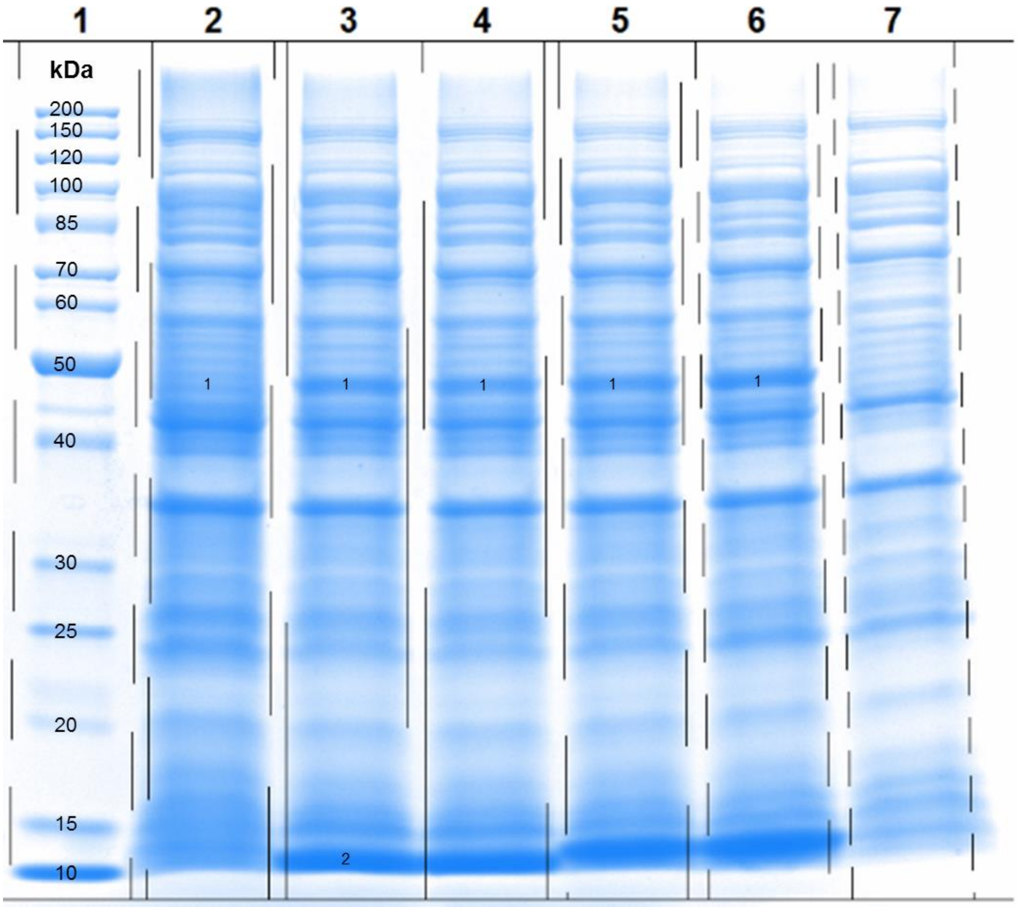

Table S2. Description of Figure S1d

Lane #	*Lane Description*	*Protein Band #*	*Protein Identified*
1	Marker	1	CotB4
2	pACYC-Duet-1 (*afR*/*afx*, cotB4)	2	Pdx
3	pACYC-Duet-1 (*pdR*/*pdx*, cotB4)		
4	pACYC-Duet-1 (*pdR*/*pdx*-D38E, cotB4)		
5	pACYC-Duet-1 (*pdR*/*pdx*-D38E, cotB4)		
6	pACYC-Duet-1 (*pdR*/*pdx*-D38E/W106E, cotB4)		
7	pACYC-Duet-1 (empty)		

2. Bioinformatics

Figure S2a. Model of CotB3 harboring cyclooct-9-en-7-ol. Modelled CotB3 (gray), prosthetic heme group (magenta) and cyclooct-9-en-7-ol (blue).

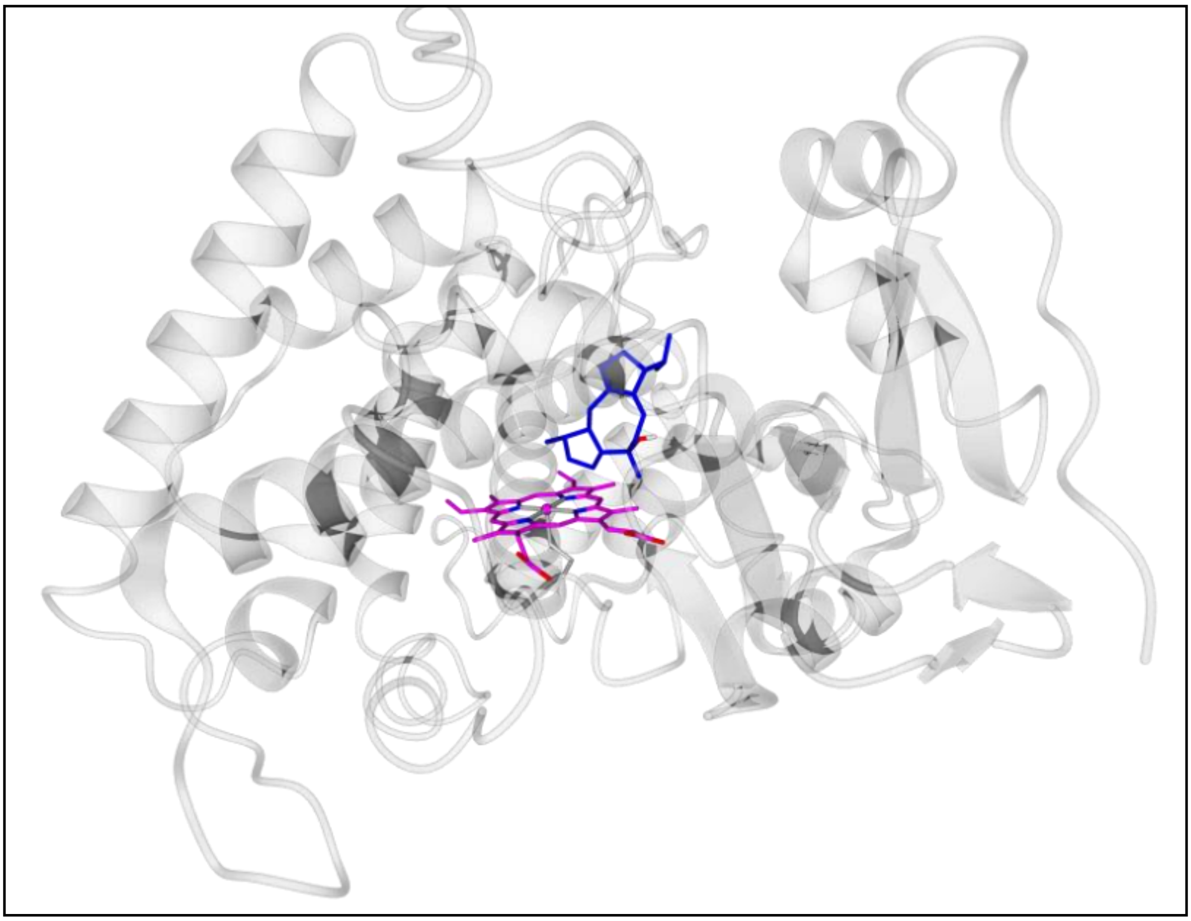

Figure S2b. Structural alignment of CotB3 (cyan) and P450cam (PDB-ID: 4JX1, red). The alignment has an RMSD of 0.142 Å over 313 aligned residues with 19% sequence identity. Note that the RMSD value considers only atoms that are present in both structures. Not matching loops in CotB3 are not incorporated into the alignment. The interaction region of CotB3 with Afx/Pdx is part of the matched helical structures.

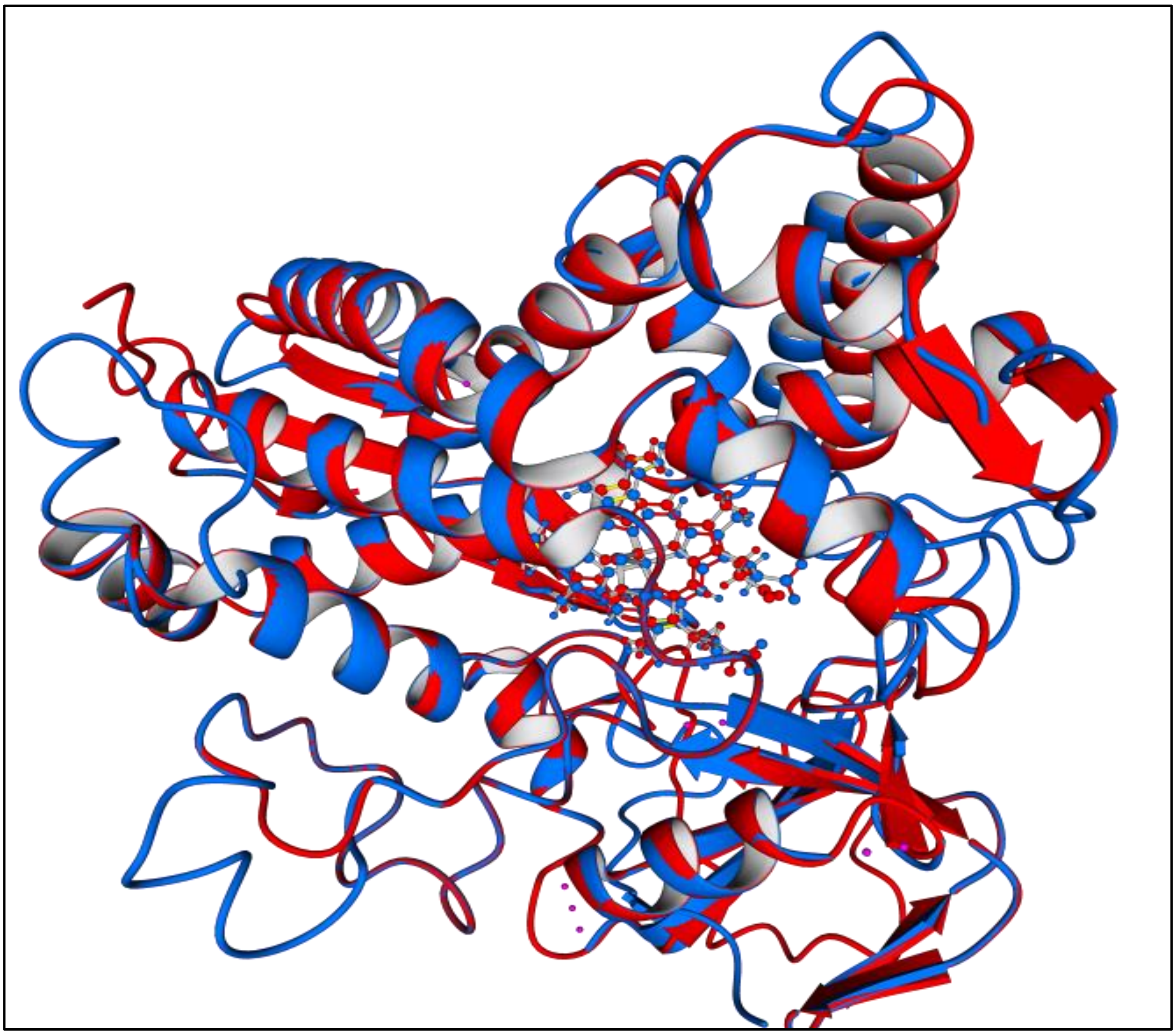

Figure S2c. Model of CotB4 harboring cyclooct-9-en-5,7-diol. Modelled CotB4 (gray), prosthetic heme group (magenta) and cyclooct-9-en-5,7-diol (blue).

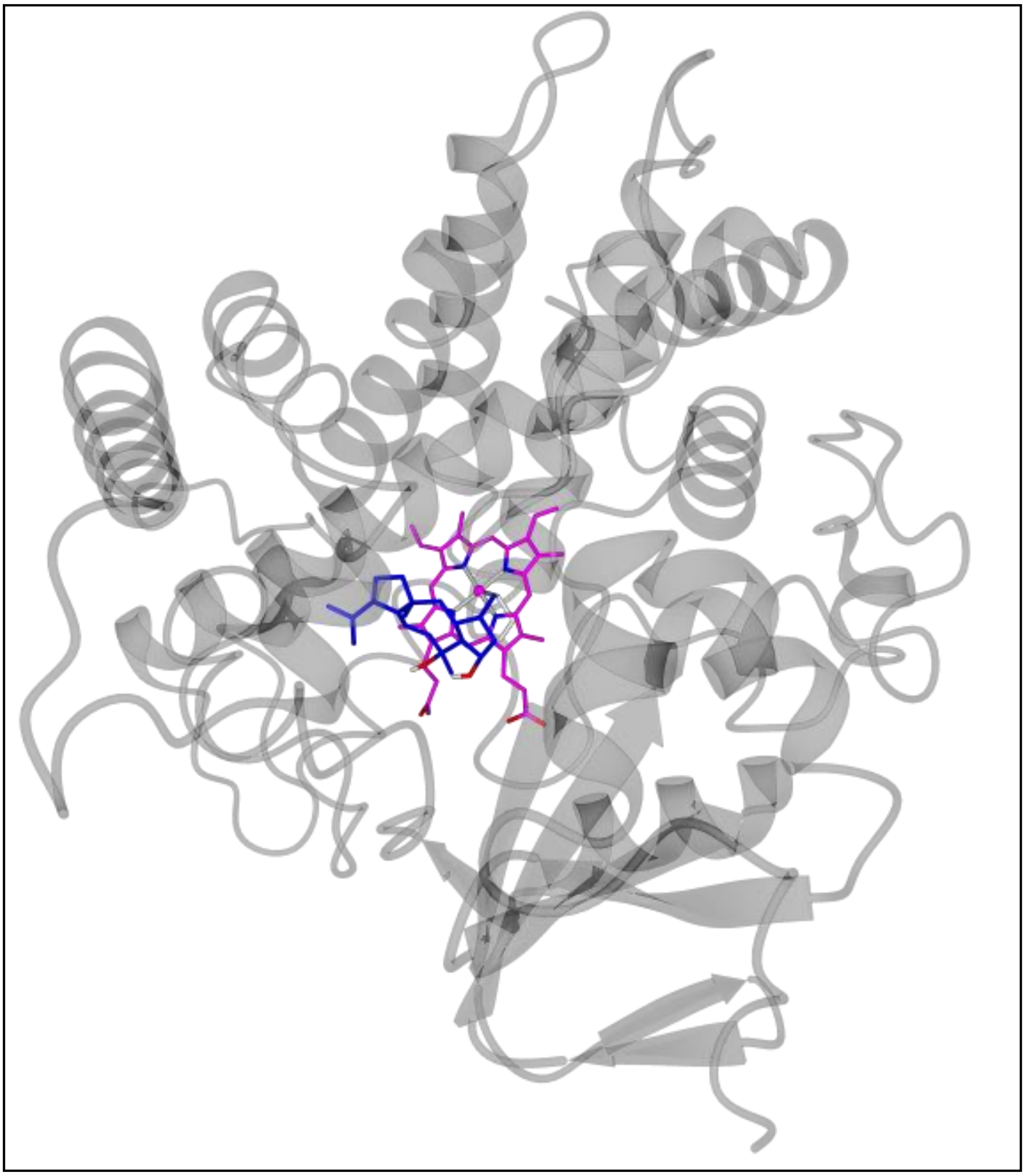

Fig S2d. Structural alignment of CotB4 (cyan) and P450cam (PDB-ID: 4JX1, red). The alignment has an RMSD of 0.144 Å over 339 aligned residues with 18% sequence identity. Note that the RMSD value considers only atoms that are present in both structures. Not matching loops in CotB4 are not incorporated into the alignment. The interaction region of CotB4 with Afx/Pdx is part of the matched helical structures.

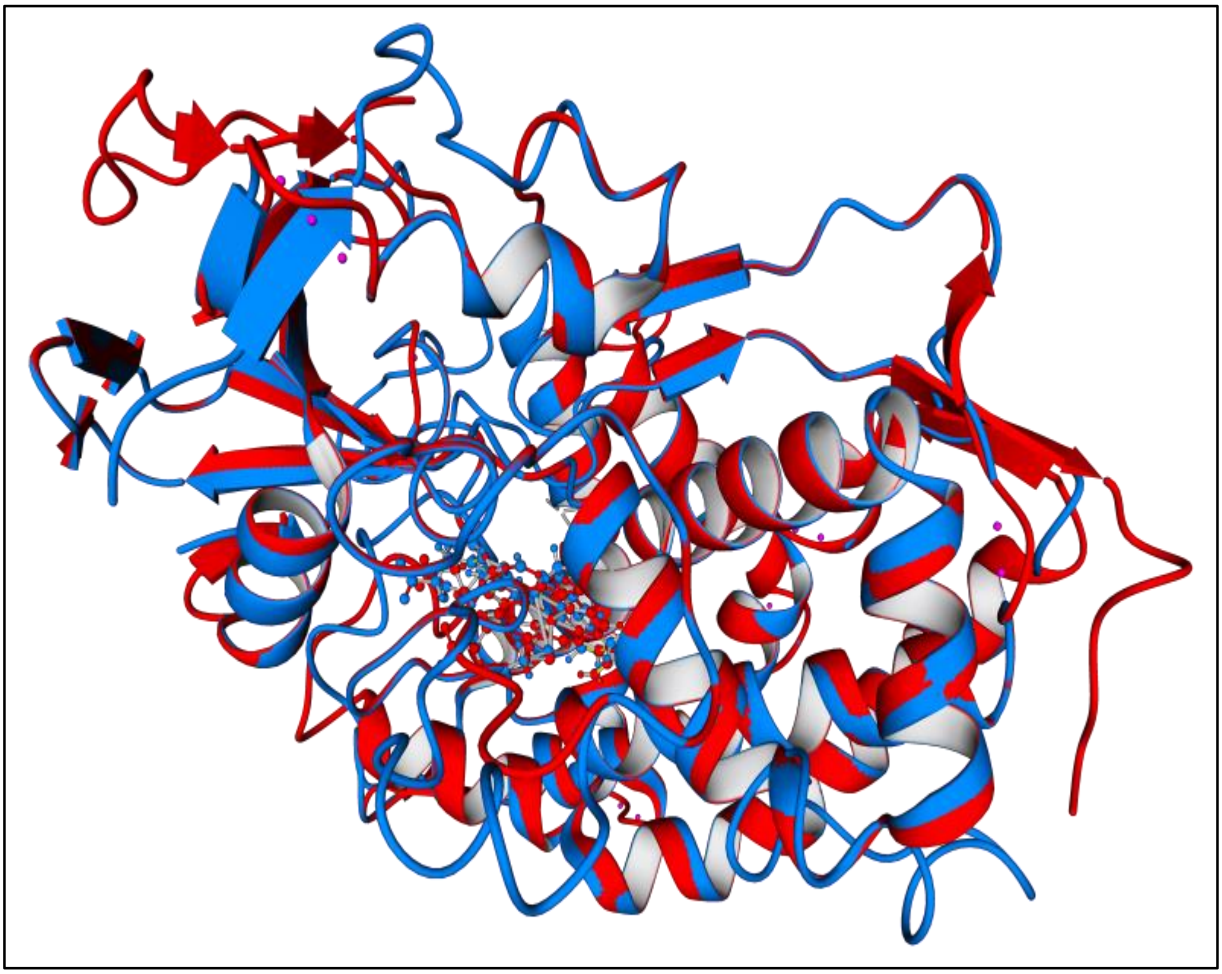

Figure S2e. Modelled AfR•Afx complex with AfR harboring FAD (Flavin-Adenine-Dinucleotide) and Afx harboring inorganic Fe_2/S_2-cluster. AfR•Afx complex (dark blue/gray). AfR with bound prosthetic FAD (magenta) and Afx bound to inorganic Fe_2/S_2-cluster (light blue). W297 of AfR proposed to be acting in electron transfer is shown in red.

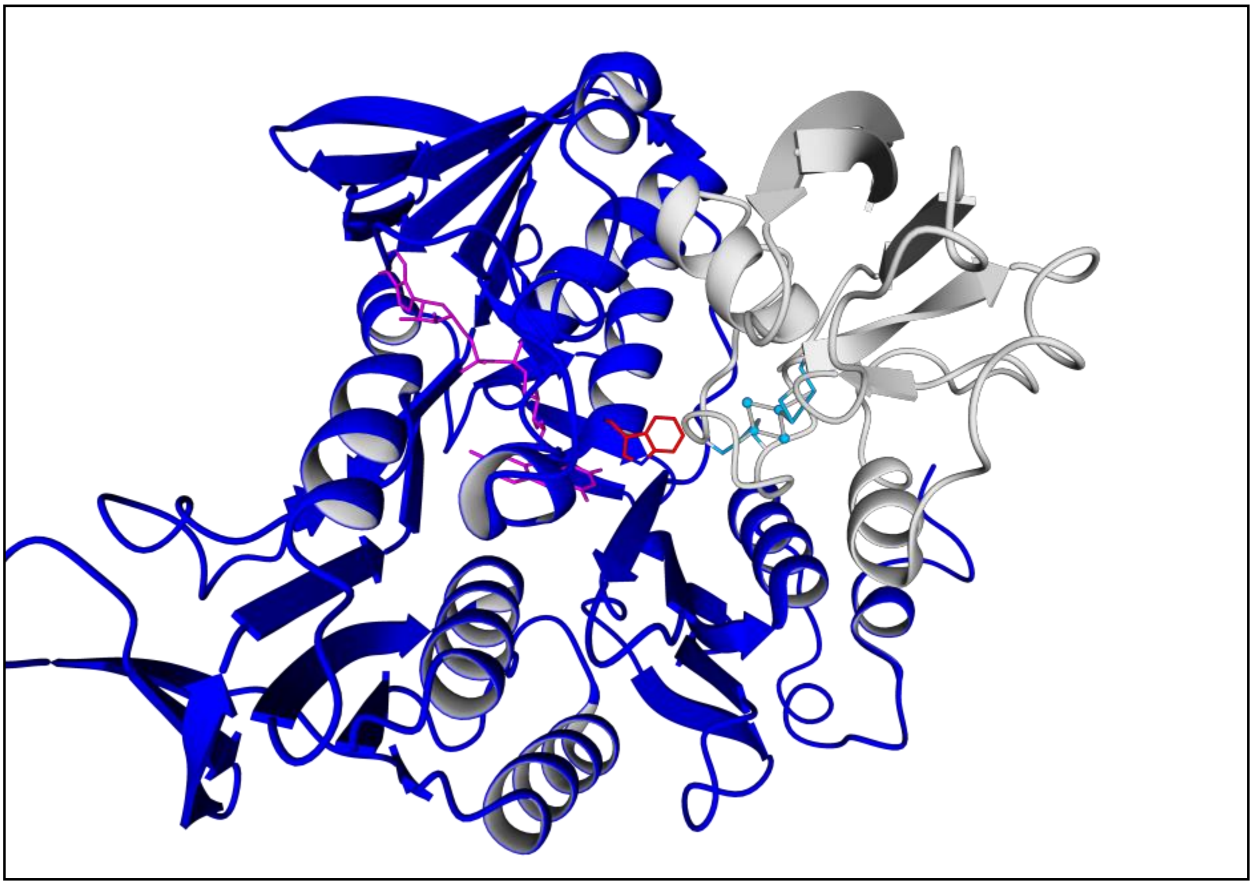

Figure S2f. Modelled CotB3•Afx complex harboring cyclooct-9-en-7-ol. CotB3•Afx complex (dark blue/orange). CotB3 contains prosthetic heme group (magenta) and cyclooct-9-en-7-ol (light blue). Afx bound to inorganic Fe_2/S_2-cluster (green/magenta).

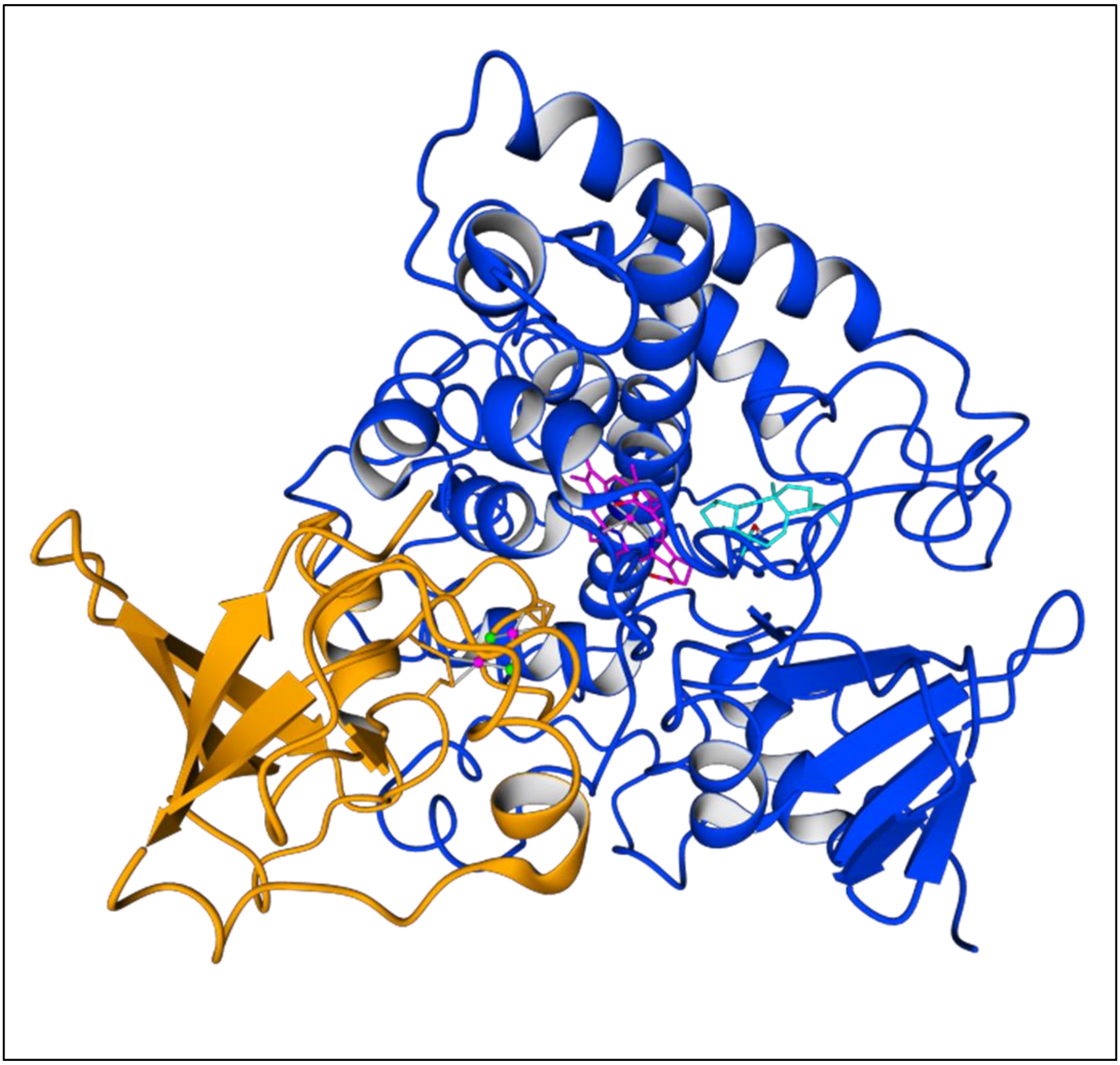

Figure S2g. Modelled CotB4•Afx complex harboring cyclooct-9-en-7-ol. CotB4•Afx complex (dark blue/orange). CotB4 contains prosthetic heme group (magenta) and cyclooct-9-en-5,7-diol (light blue). Afx contains inorganic Fe2/S2-cluster (green/magenta).

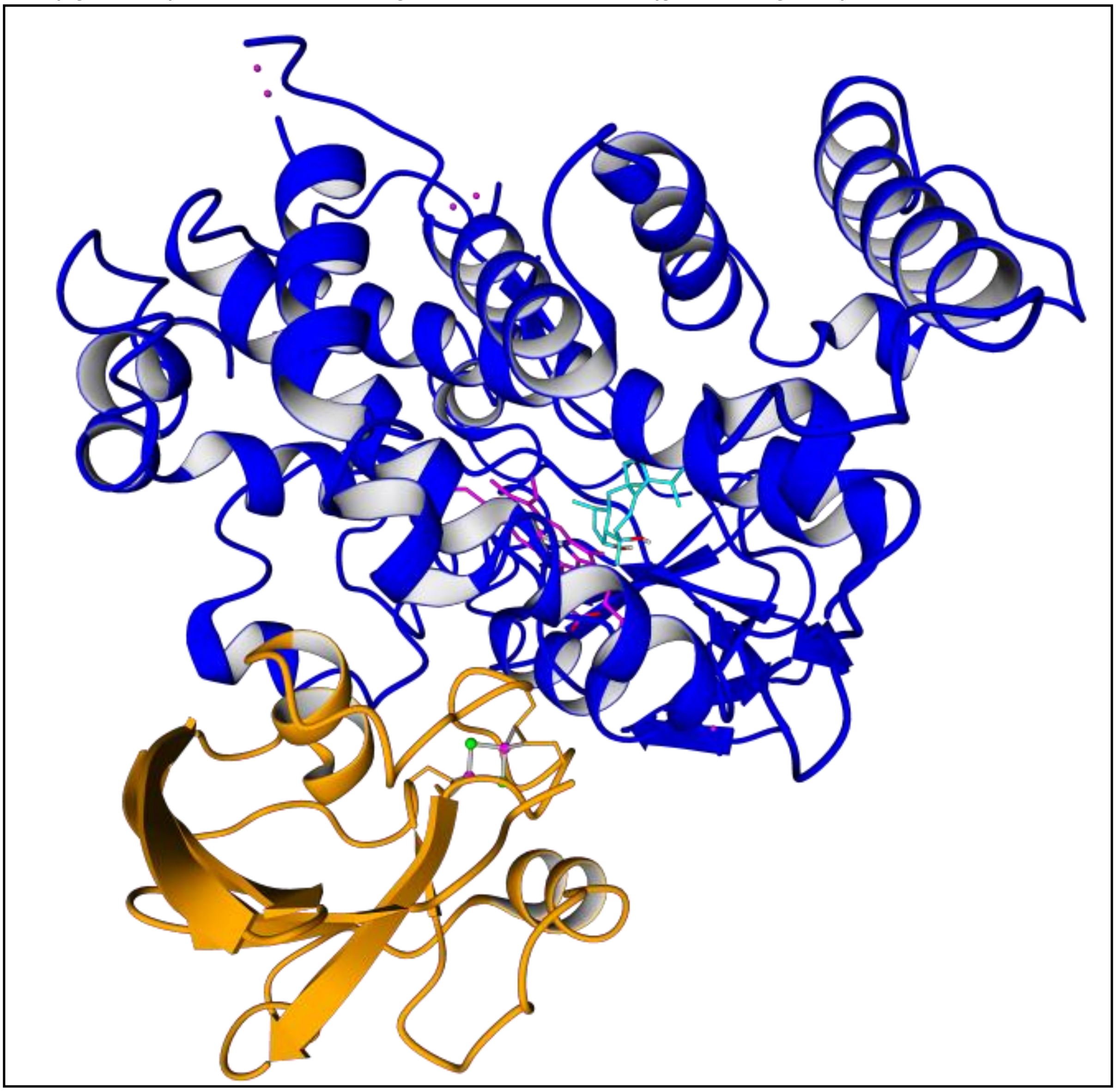

Figure S2h. Inner view of the Cotb3•Pdx complex (dark blue•red). Pdx contains inorganic Fe2/S2-cluster (green/magenta). CotB3 contains prosthetic heme group (gray) and cyclooct-9-en-7-ol (red). Essential hydrogen bonds for the interaction of Pdx and CotB3 to facilitate binding and electron transport are highlighted in black. Hydrogen bond formation between $CotB3_{R110}$ and $CotB3_{R114}$ with Pdx_{W106} needed for efficient binding and electron transfer, is strongly impaired due to the chemical nature of participating amino acids. The Cotb4•Pdx complex exhibits the same structure and also the same interacting residues.

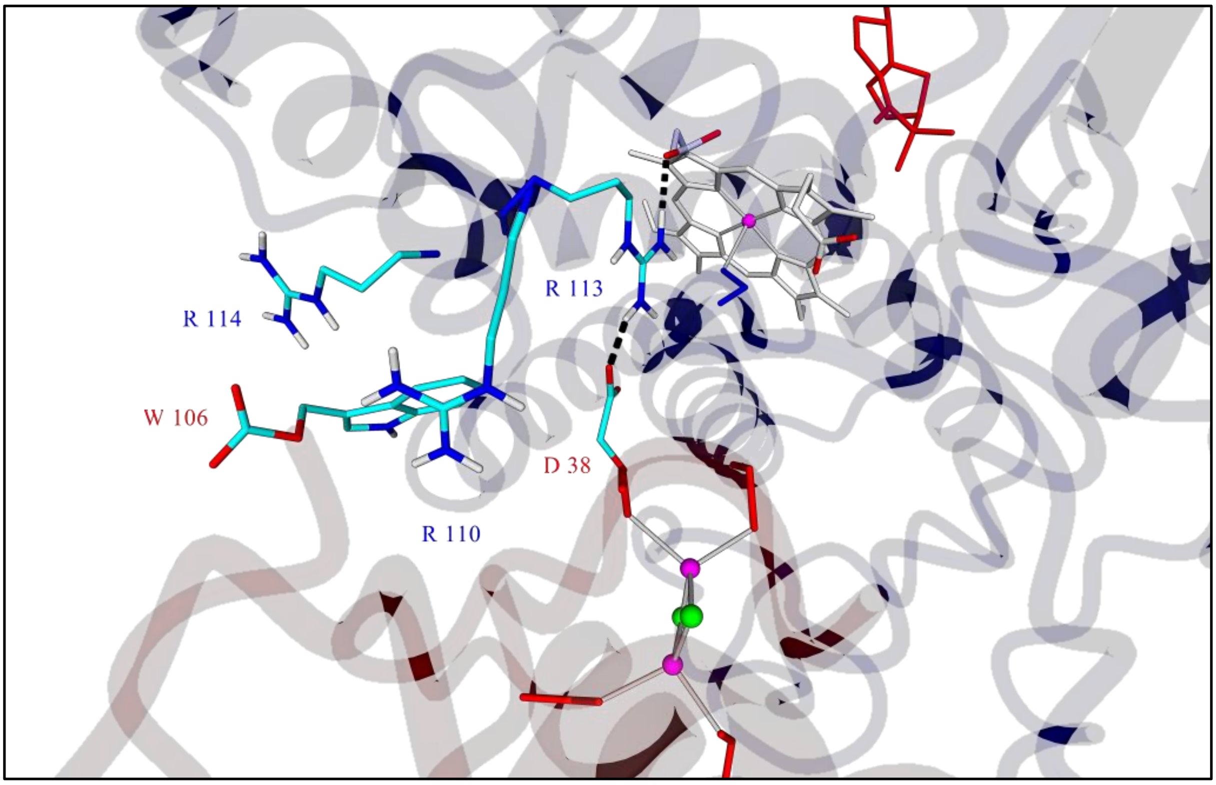

Figure S2i. Inner view of the Cotb4•Afx complex (dark blue•red). Afx contains inorganic Fe_2/S_2-cluster (green/magenta). CotB4 contains prosthetic heme group (gray) and cyclooct-9-en-5,7-diol (red). Essential hydrogen bonds for the interaction of Afx and CotB4 to facilitate binding and electron transport are highlighted in black.

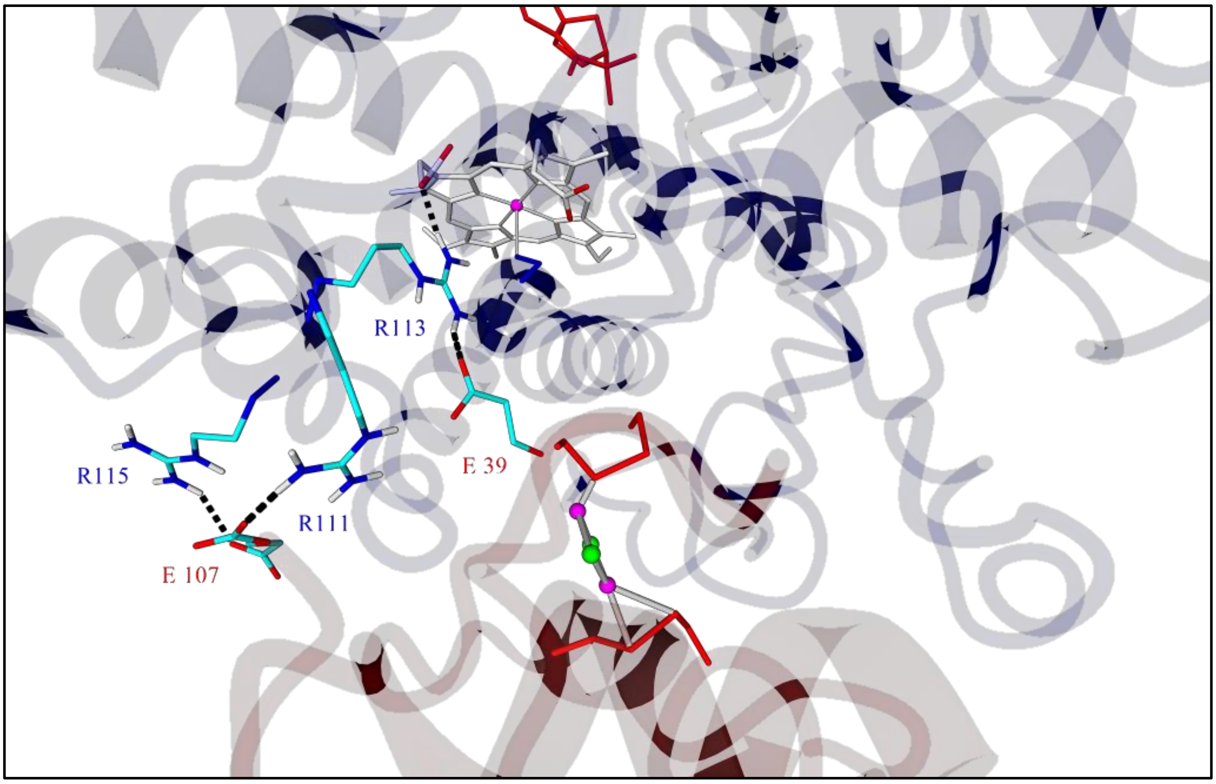

Figure S2j. Structural superposition of the PdR•Pdx (red·blue) with the modelled AfR•Afx complex (ocher•gray). Prosthetic FAD group of PdR and modelled AfR are shown in light blue. 2Fe·2S cluster of Pdx and modelled Afx are shown in magenta/green. The important hydrogen bridge (black) between Pdx_{D38} and PdR_{R310} needed for binding as well as PdR_{W330} facilitating electron transport are shown. Differing position of PdR_{W330} corresponding to W297 in AfR, presumably acting in electron transfer from FAD to Pdx is shown in ocher. Analogous interactions facilitating binding of AfR to Afx could not be deciphered. Substitution of Pdx_{D38} to E38 is suggested to impair electron transport by an alternate binding mode of Pdx_{D38E} to native PdR.

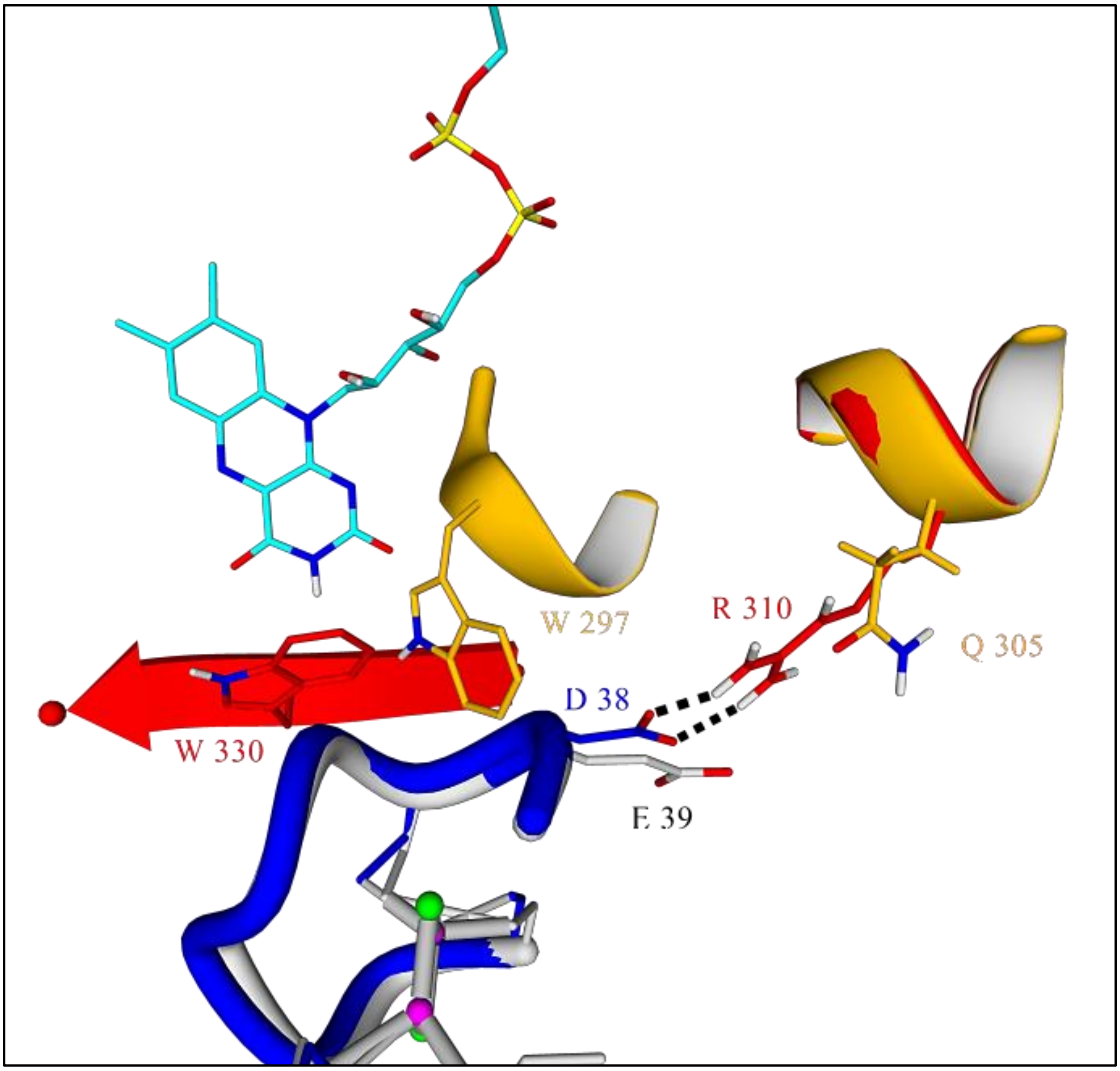

Figure S2k. Modelled CotB3 harboring (-)-casbene. CotB3 (light purple) contains prosthetic heme group (blue) and docked (-)-casbene (gray). The heme group contains the iron cation (magenta) and the dative bond to cysteine 408 (green) of the CotB3. (-)-Casbene is shown in the conformation comprising the lowest binding energy derived by cluster analysis of AutodockVina. Hydroxylated C-10 of (-)-casbene is shown in blue.

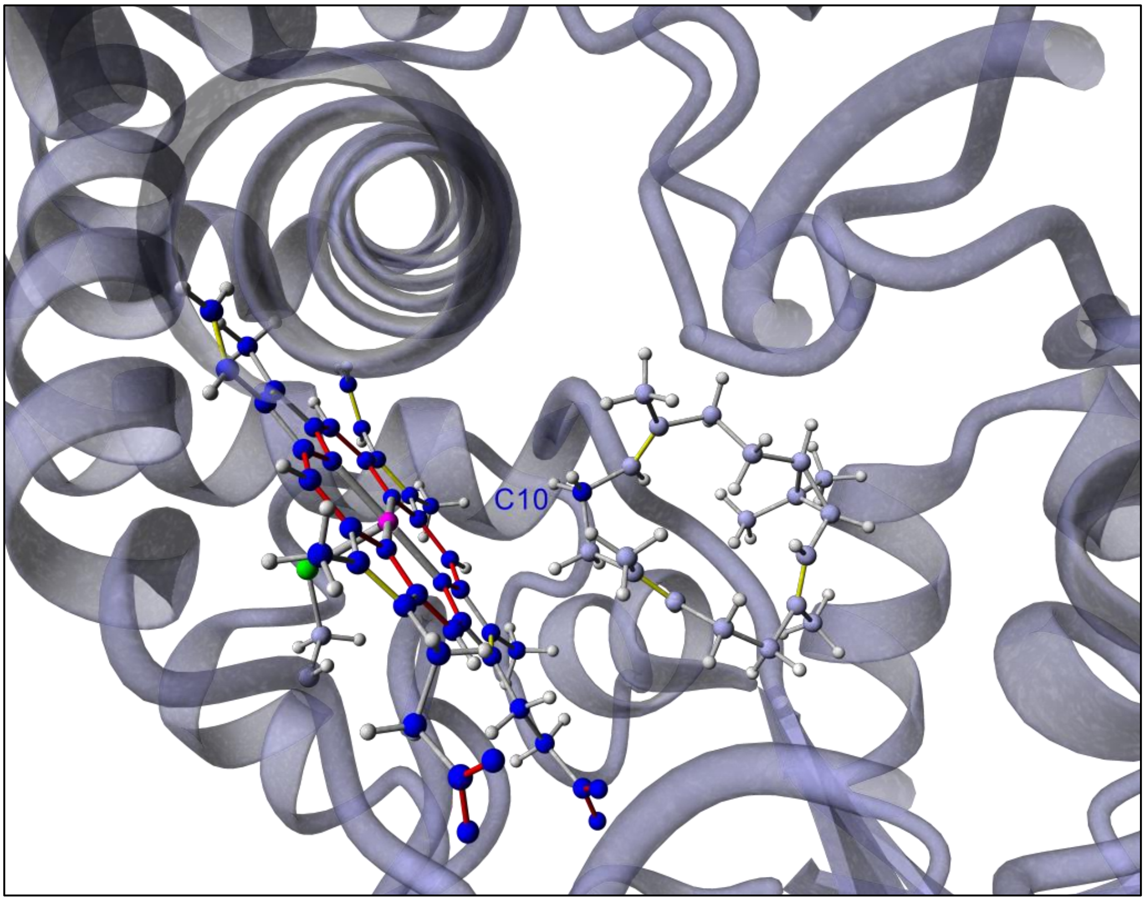

Figure S2l. Modelled CotB3 harboring sinularcasbane D. CotB3 (light purple) contains prosthetic heme group (blue) and docked sinularcasbane D (gray). The heme group contains the iron cation (magenta) and the dative bond to cysteine 408 (green) of the CotB3. Sinularcasbane D is shown in the conformation comprising the lowest binding energy derived by cluster analysis of AutodockVina.

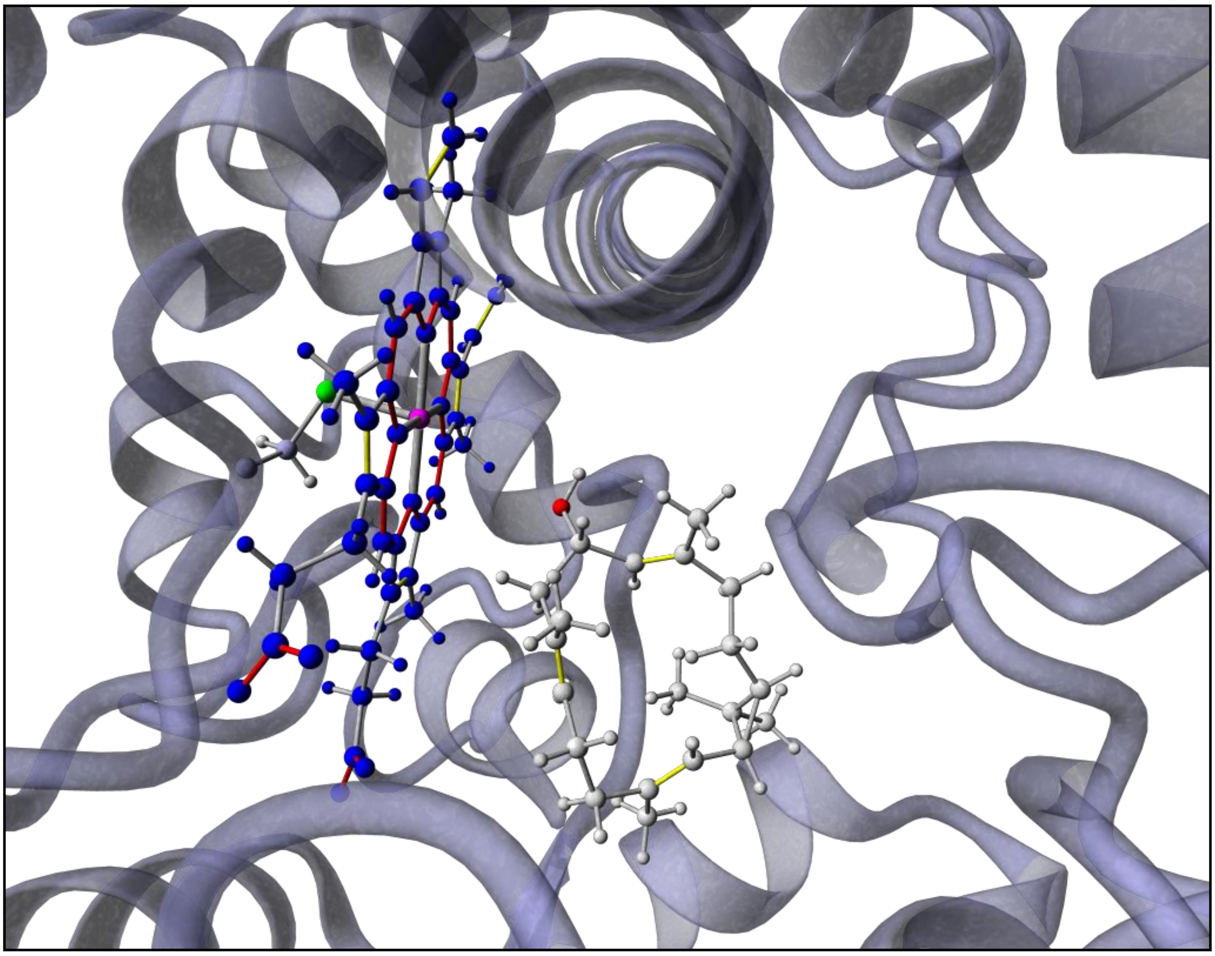

Table S3. PROCHECK results of CotB3/4, AfR and Afx.

	Most favored regions in RAMACHANDRAN Plot [%]	G-factor/ dihedral angles [average score]	G-factor/Main-chain covalent forces [average score]
CotB3	92.5	-0.20	-0.29
CotB4	90.4	-0.27	-0.32
AfR	92.8	-0.01	-0.13
Afx	94.1	-0.02	-0.09

Our models exceed the demanded 90% of the RAMACHANDRAN Plot factoring, which indicates that these are excellent models. Also the G-factors are all above the -0.5 threshold indicating that no unusual folding patterns occur.

QMEAN Z-scores

Figure S2m. QMEAN Z-score of the AfR model (-0.31).

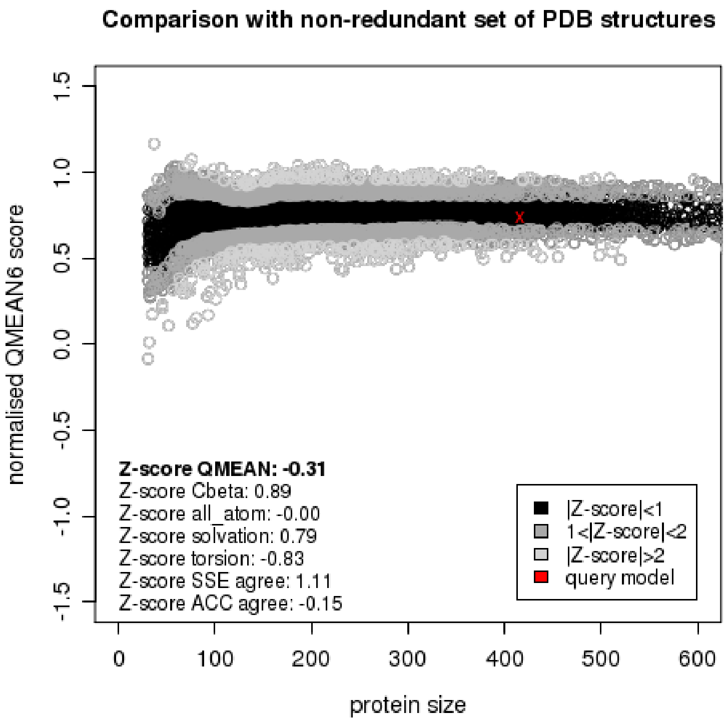

Figure S2n. QMEAN Z-score of the Afx model (-0.10).

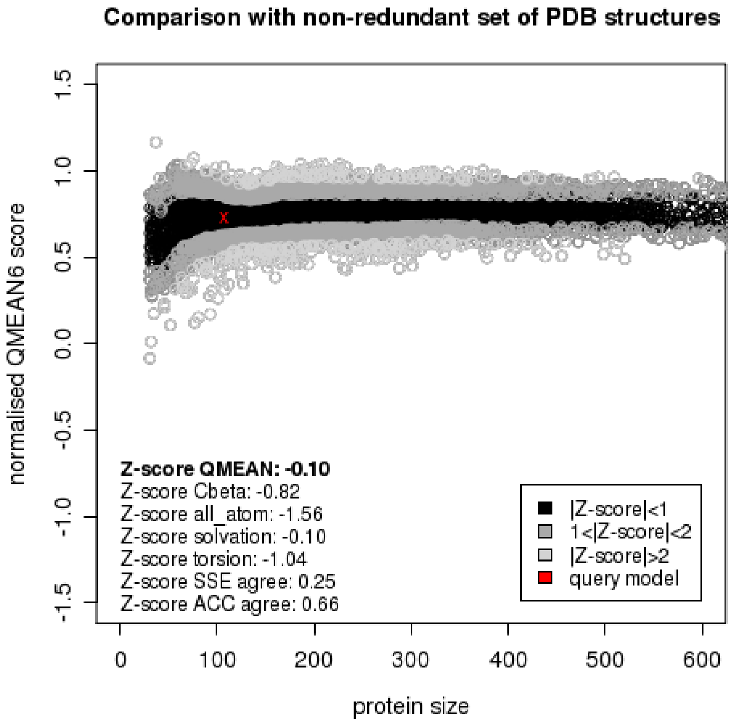

Figure S2o. QMEAN Z-score of the CotB3 model (-1.15).

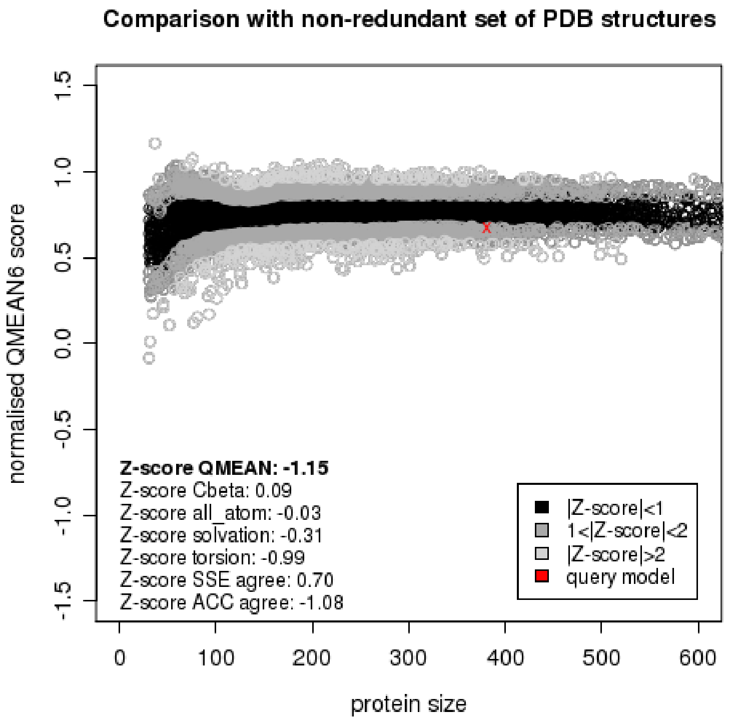

Figure S2p. QMEAN Z-score of the CotB4 model (-1.33).

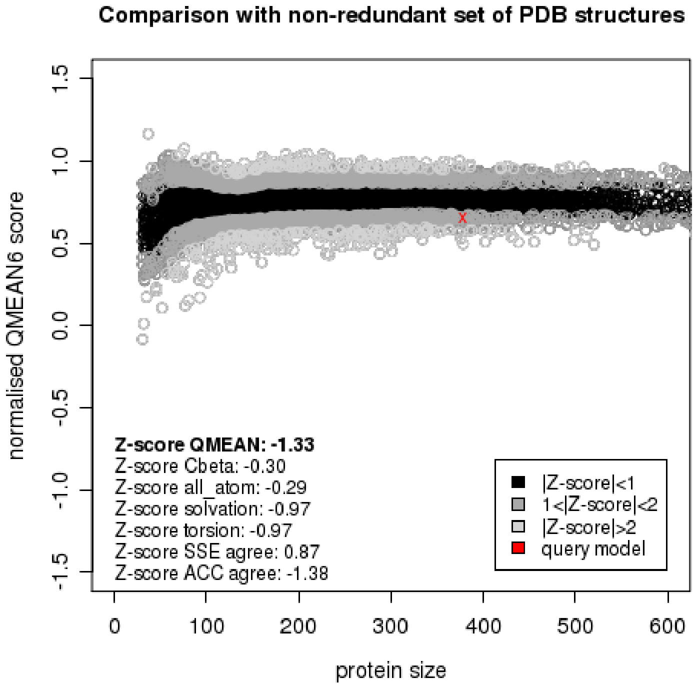

The QMEAN Z-score of the aforementioned modeled proteins suggest that these models are of comparable quality to high resolution experimental structures. The reference structures are a non-redundant subset of the PDB sharing less than 30% sequence identity among each other and are solved at a resolution below than 2 Å.

The structural overlap of the PdR•Pdx and AfR•Afx complexes displayed high agreement and were therefore not subjected to further adjustments. In contrast, the hydroxylases CotB3/4 showed extended secondary structure motifs compared to the P450cam template. Both CotB3/4 display additional loop regions which are missing in P450cam. Consequently, the extra protein loops could not be aligned with the P450cam template (Figure S2b, d). Hence the extra loops will also complicate the quality assessments as *ab initio* loop modelling of unknown proteins are a typical source of error during modelling approaches. Therefore, we have excluded the extra loop regions in CotB3/4 in our PROCHECK and QMEAN Z-score quality assessments. As the excluded loops are neither in the vicinity of the active site, heme group or the ferredoxin interaction domain, an effect on identification of essential amino acid interactions can be excluded. In the absence of x-ray crystal structures, our modelling methodology provides the best insights into the essential protein interactions that drive the observed catalytic processes.

3. NMR-Spectroscopy

Figure S3a. 1H NMR (500 MHz, CDCl3) of cyclooctat-9-en-7-ol.

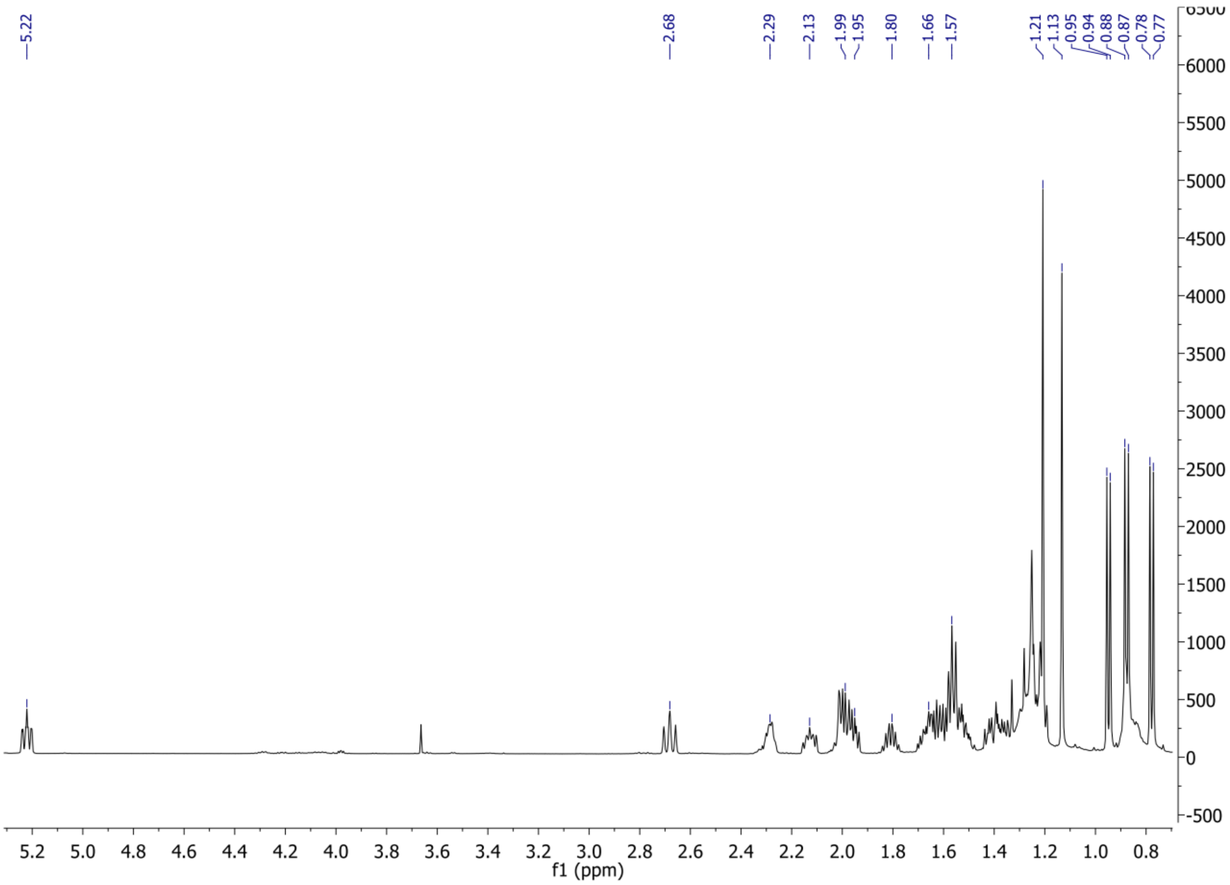

Figure S3b. ^{13}C NMR (125 MHz, $CDCl_3$) of cyclooctat-9-en-7-ol.

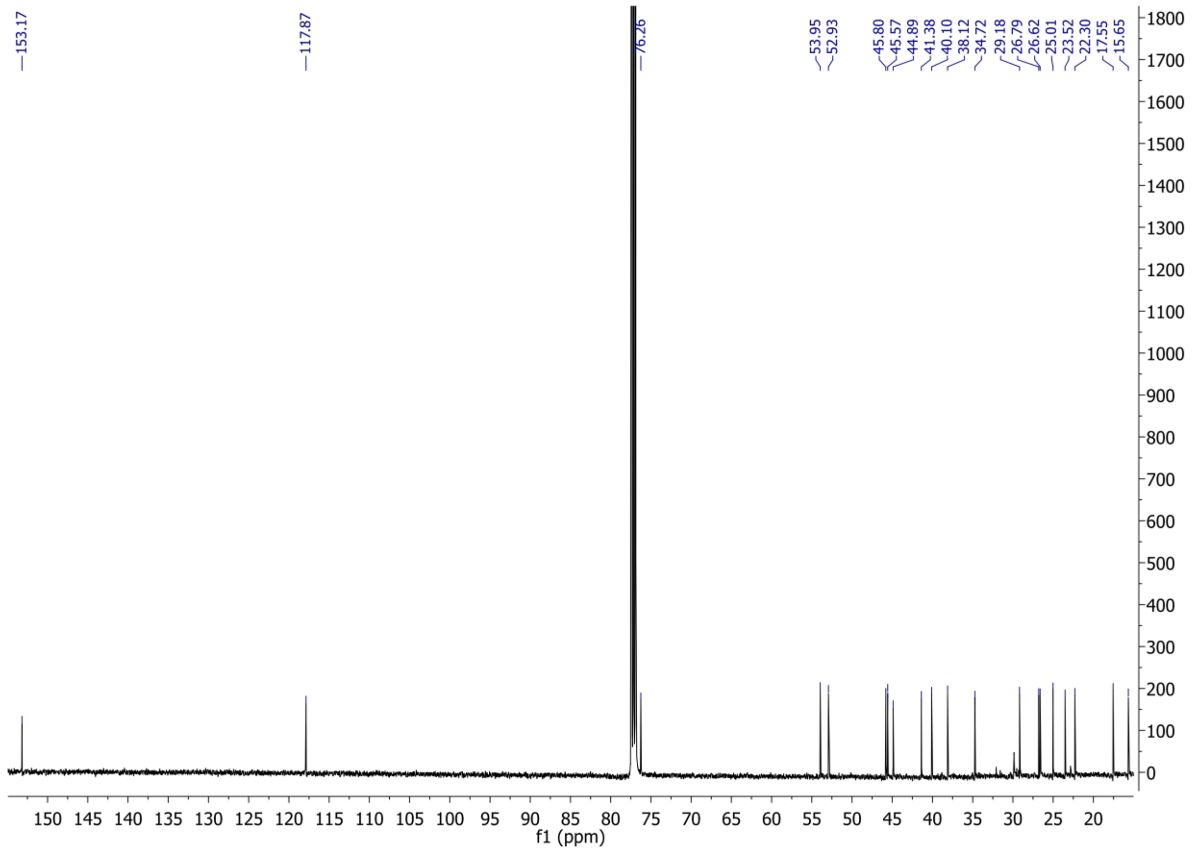

Figure S3c. ^{1}H NMR (500 MHz, $CDCl_3$) of cyclooctat-9-en-5,7-diol.

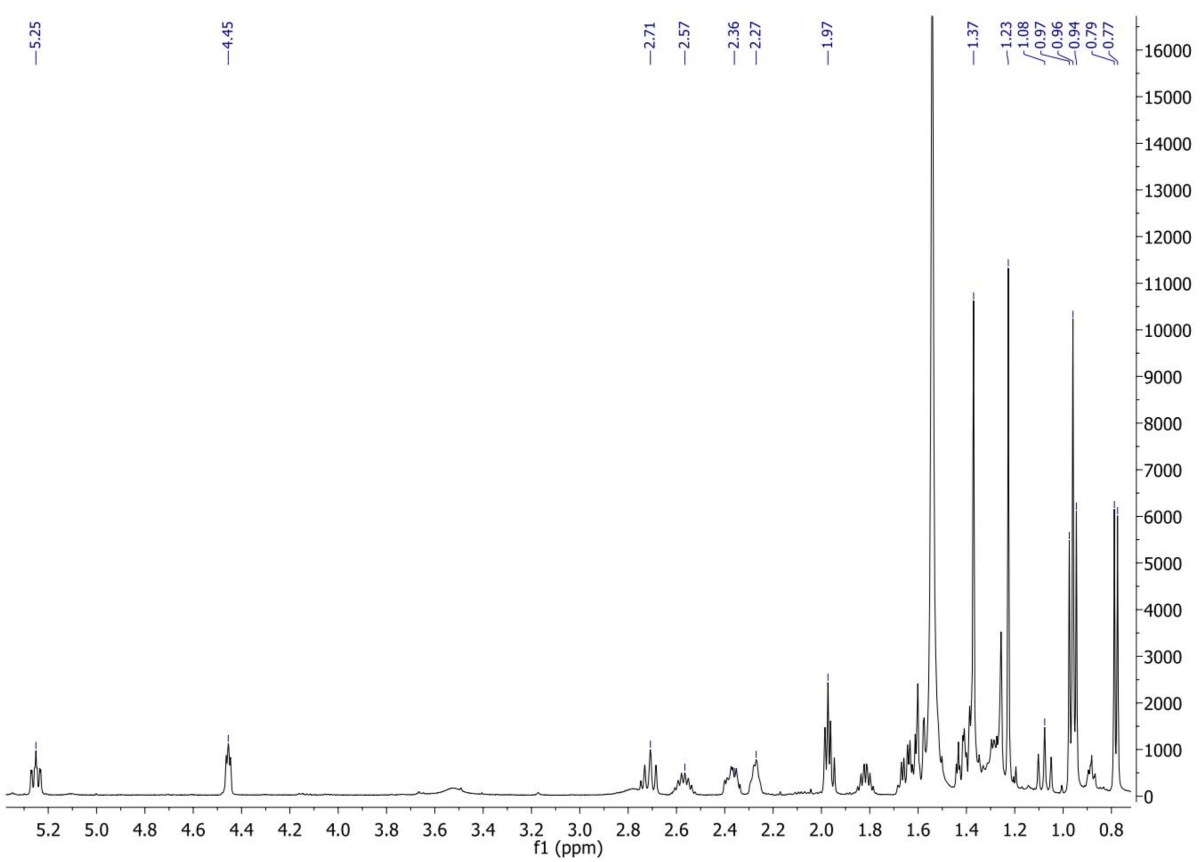

Figure S3d. ^{13}C NMR (125 MHz, $CDCl_3$) of cyclooctat-9-en-5,7-diol.

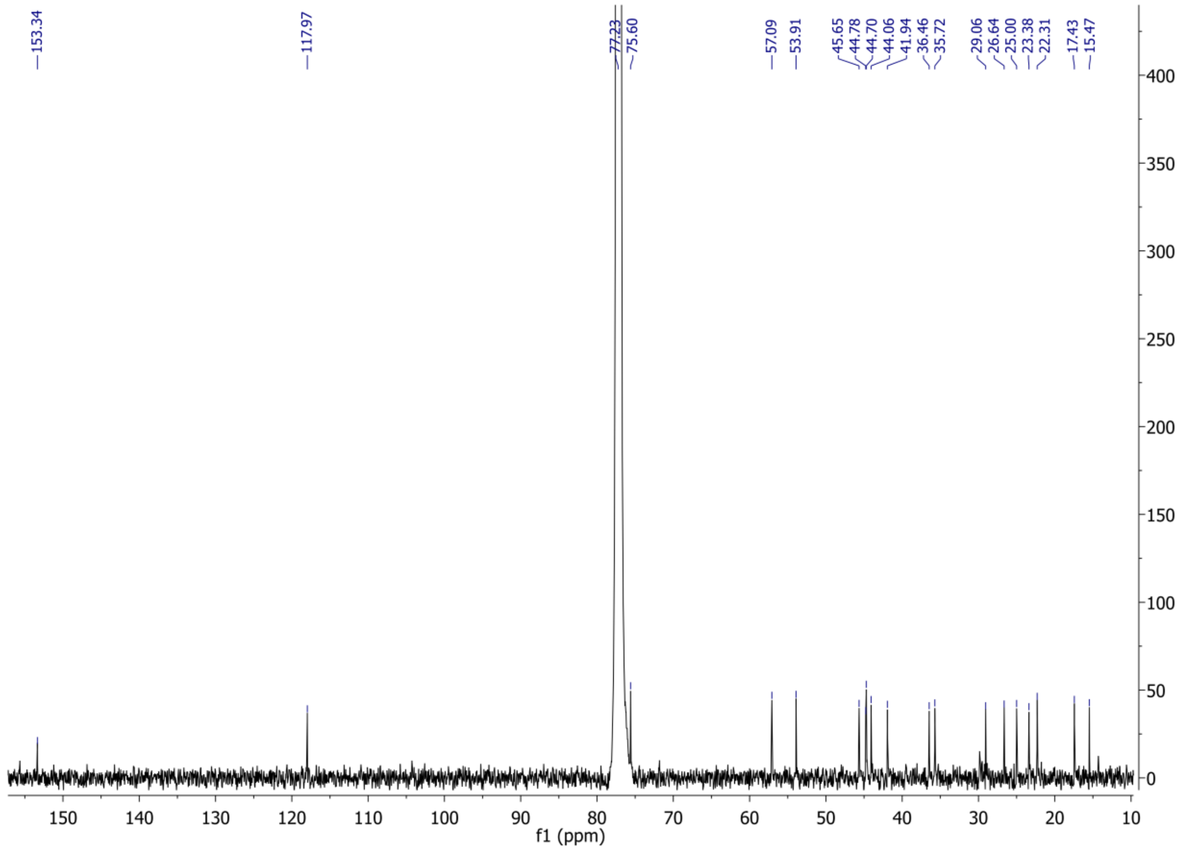

Figure S3e. ^{1}H NMR (500 MHz, CD_3-OD) of cyclooctatin.

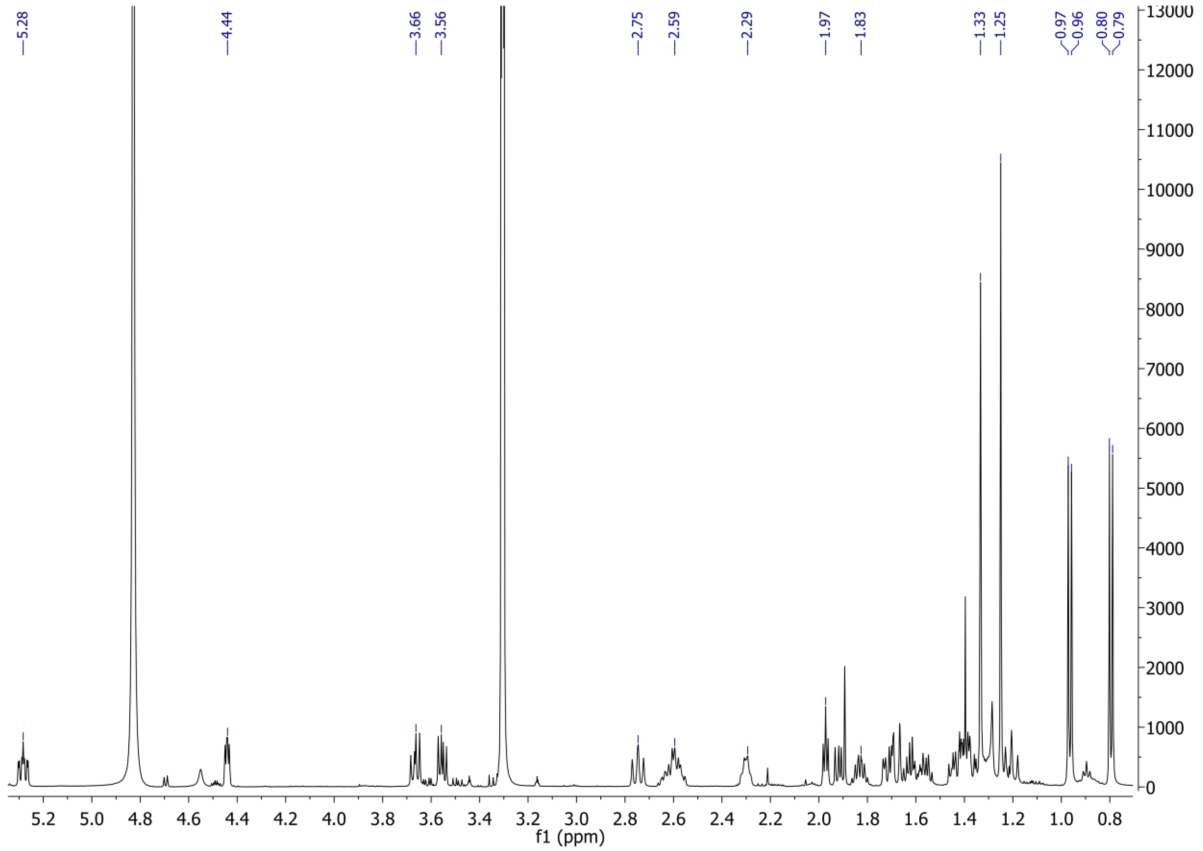

Figure S3f. ^{13}C NMR (125 MHz, CD_3-OD) of cyclooctatin.

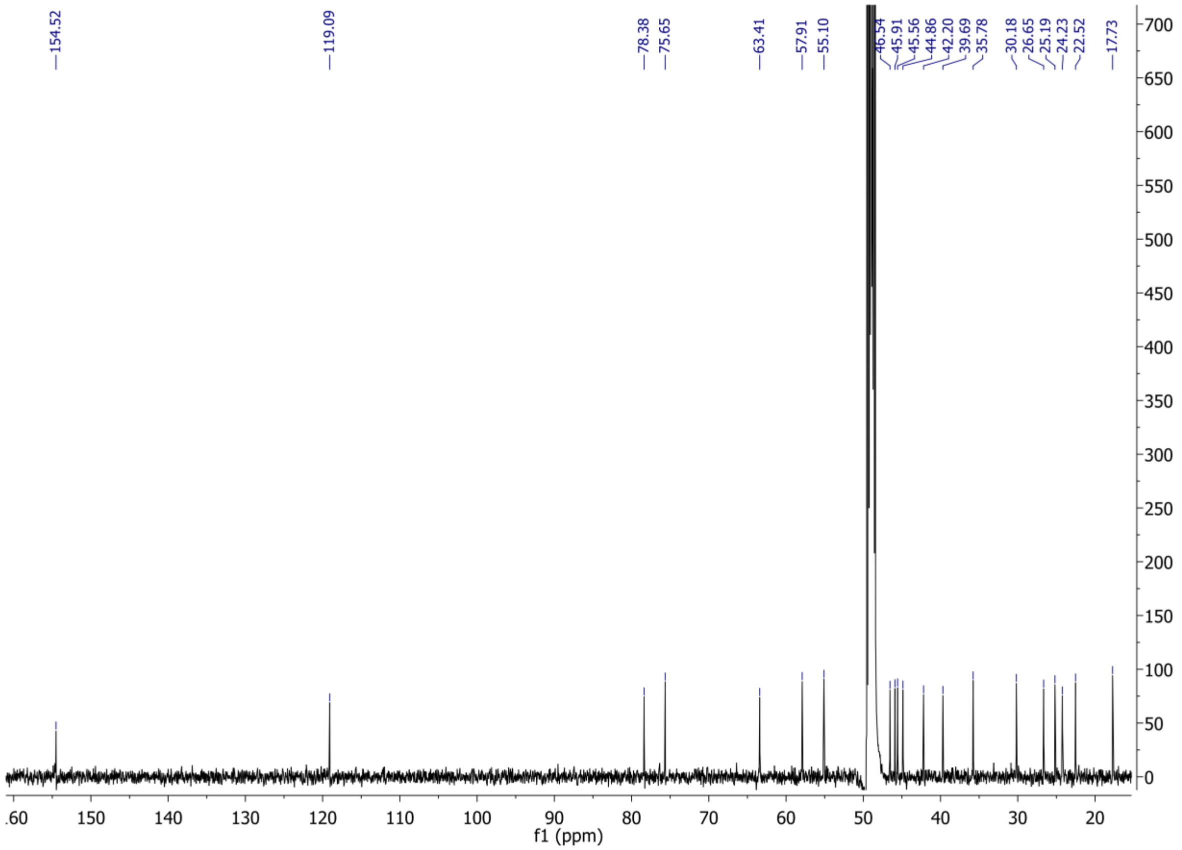

Figure S3g. COSY (500 MHz, CD_3-OD) NMR of cyclooctatin.

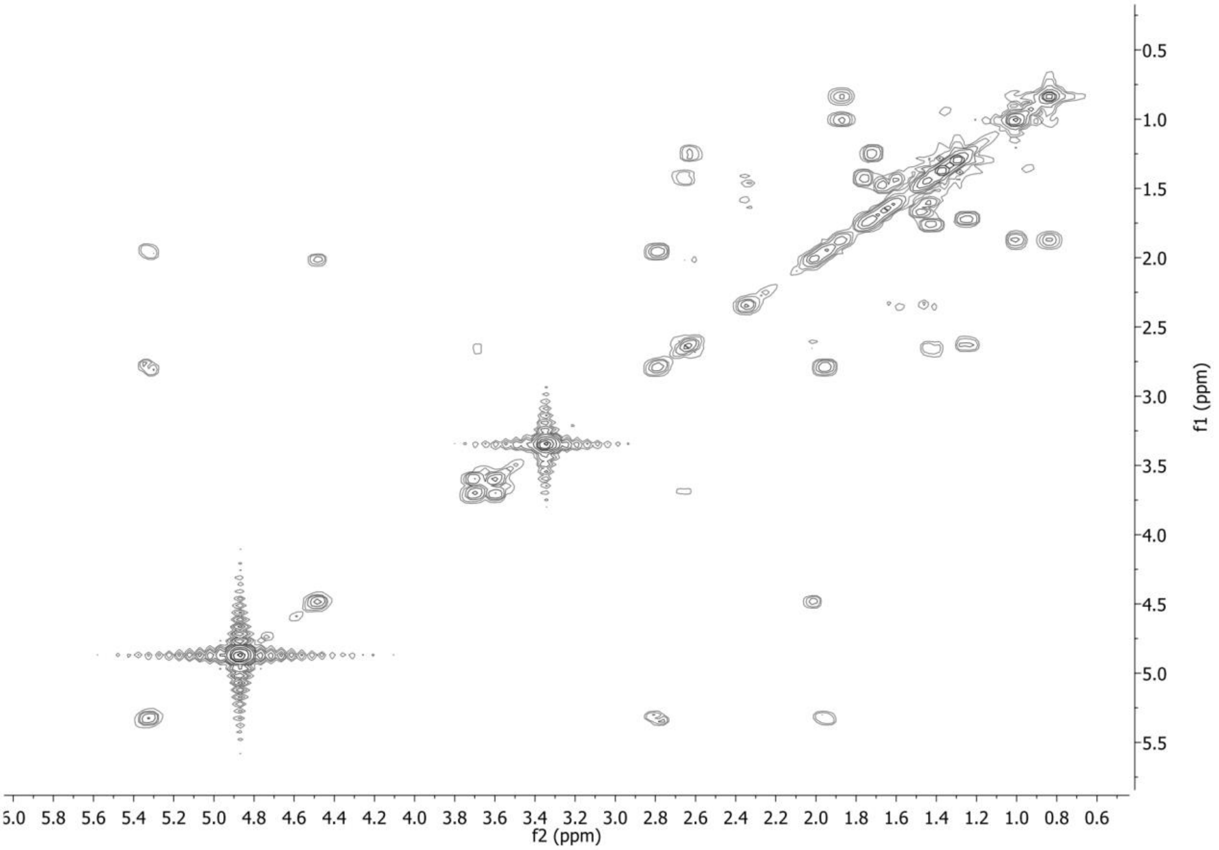

Figure S3h. HSQC (500 MHz, CD_3-OD) NMR of cyclooctatin.

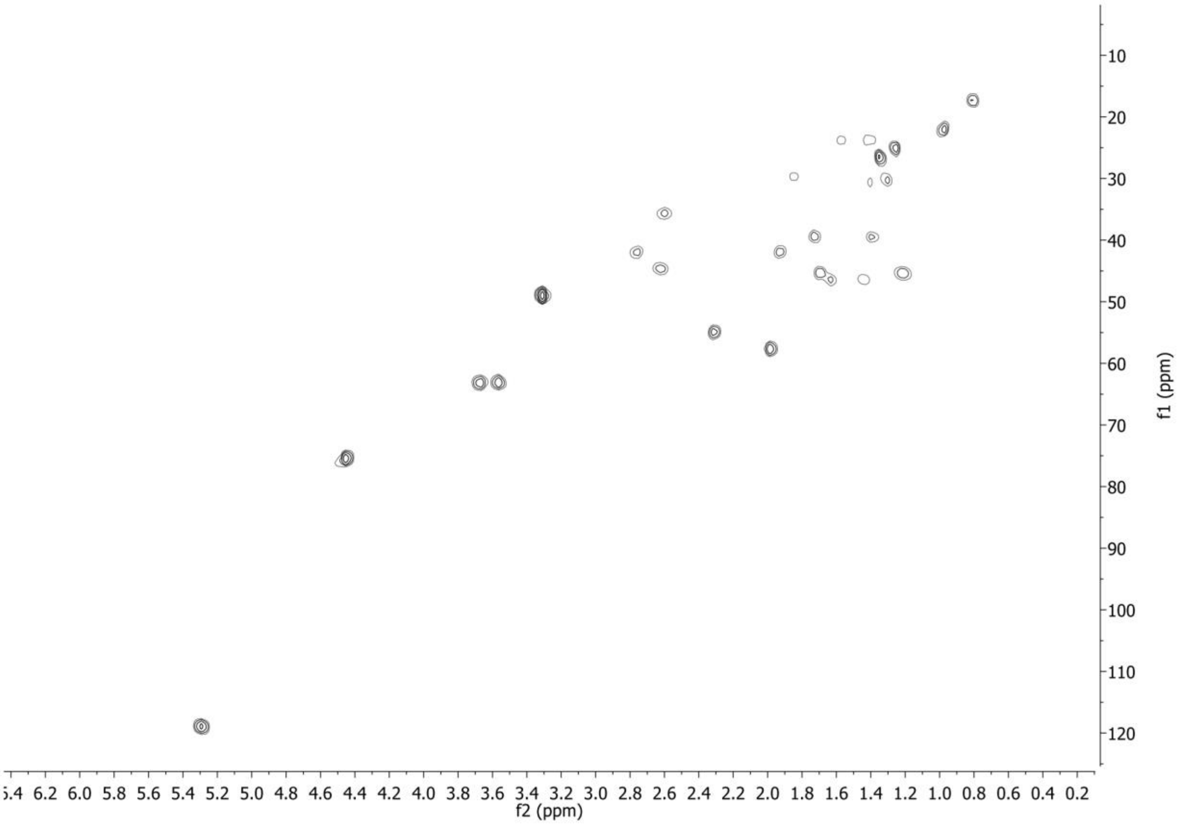

Figure S3i. NOESY NMR (500 MHz, CD3-OD) of cyclooctatin.

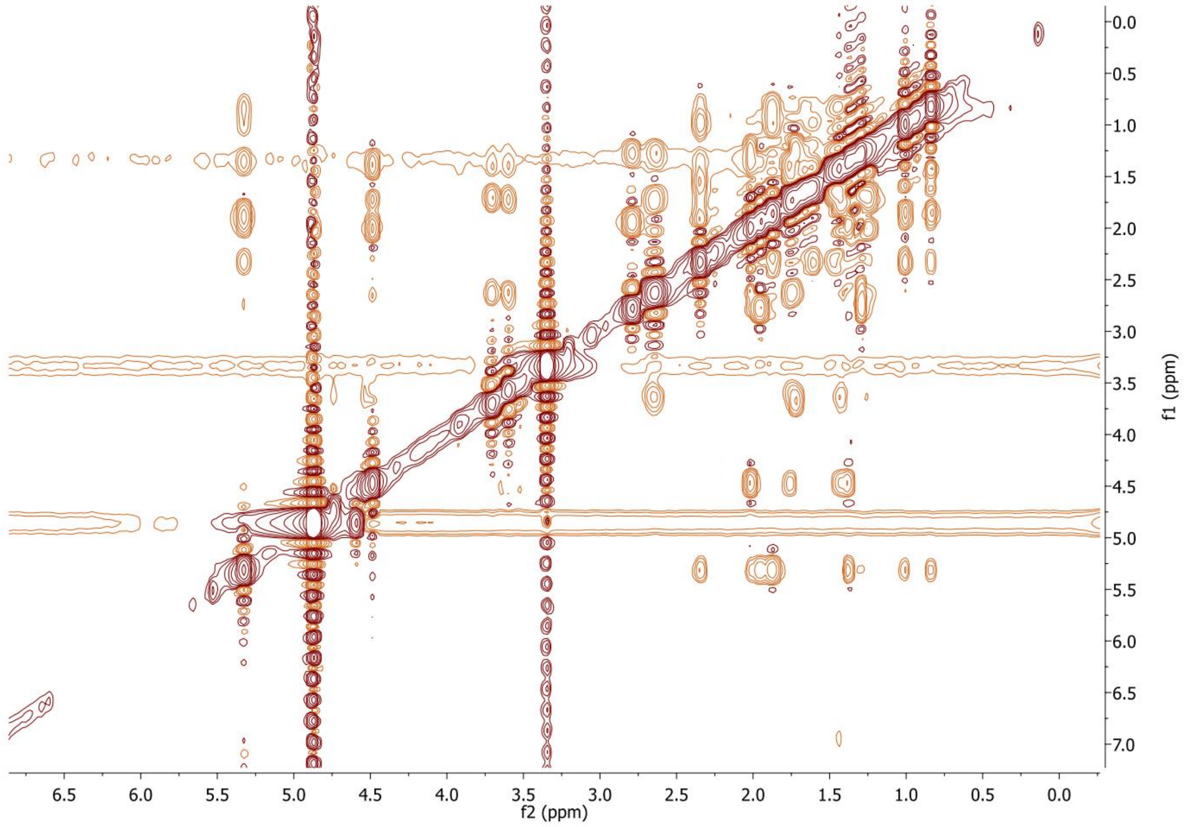

Figure S3j ^{1}H NMR (500 MHz, $CDCl_3$) of the C-5 R-MTPA derivative of cyclooctat-9-en-5,7-diol.

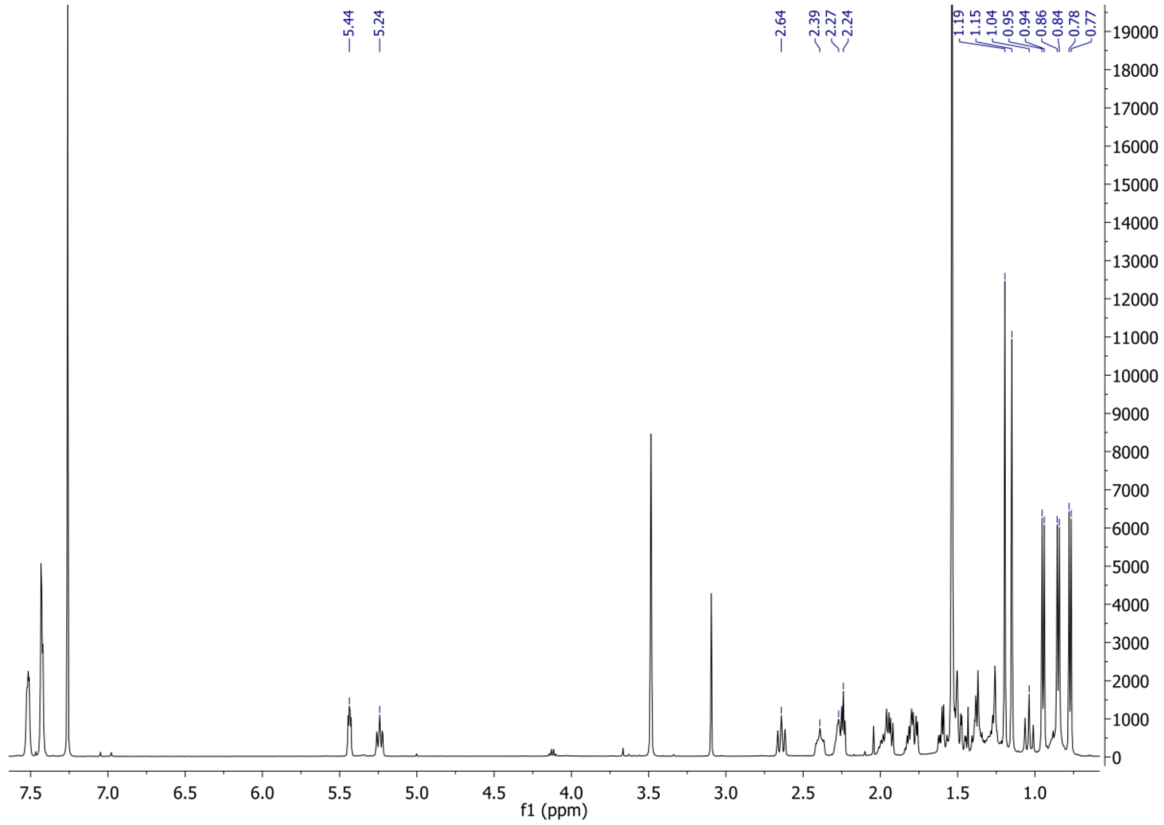

Figure S3k. ^{13}C NMR (125 MHz, $CDCl_3$) of the C-5 R-MTPA derivative of cyclooctat-9-en-5,7-diol.

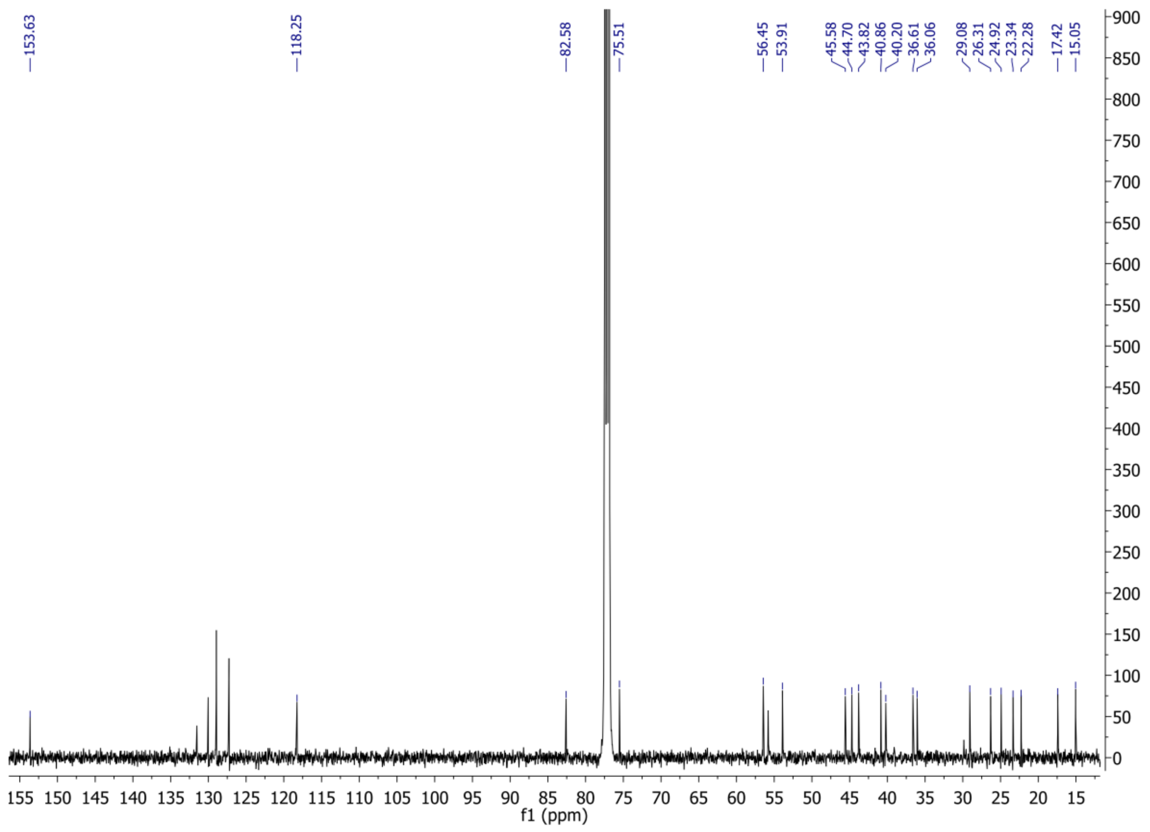

Figure S3l. COSY NMR (500 MHz, $CDCl_3$) of the C-5 R-MTPA derivative of cyclooctat-9-en-5,7-diol.

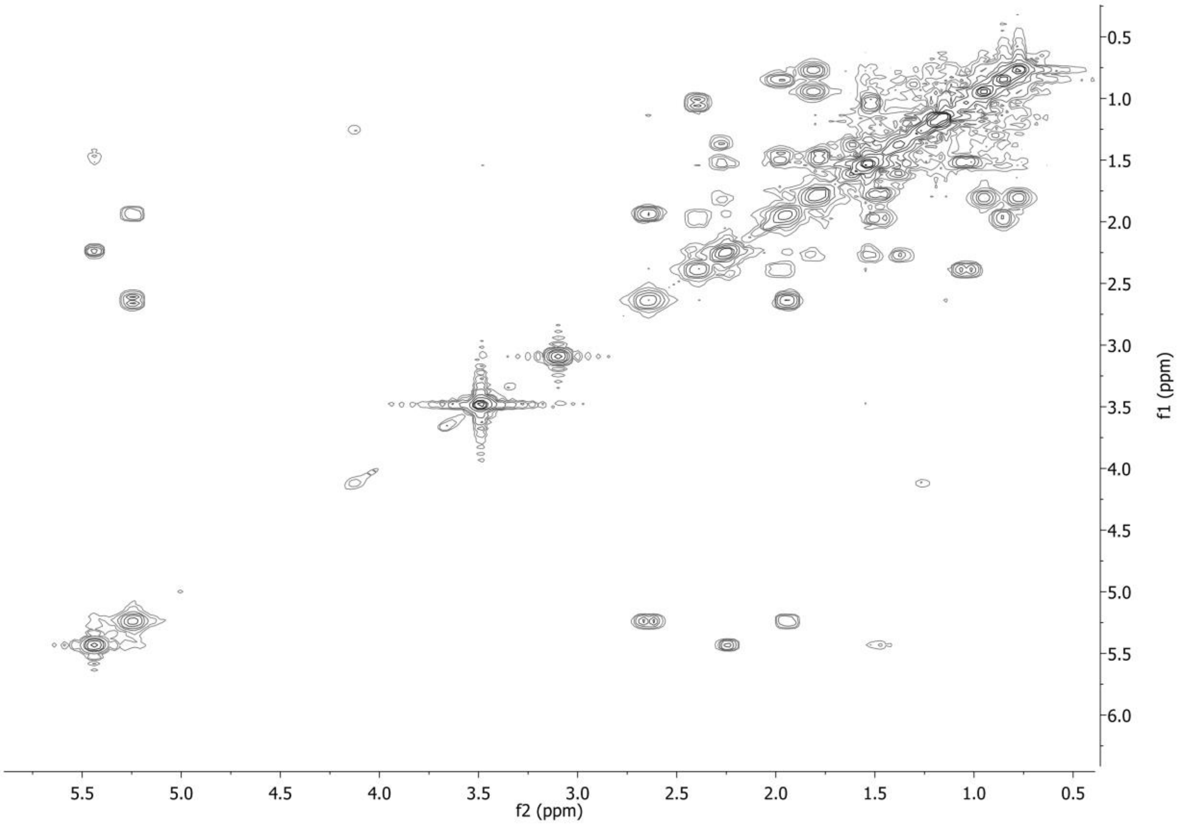

Figure S3m. HSQC NMR (500 MHz, $CDCl_3$) of the C-5 R-MTPA derivative of cyclooctat-9-en-5,7-diol.

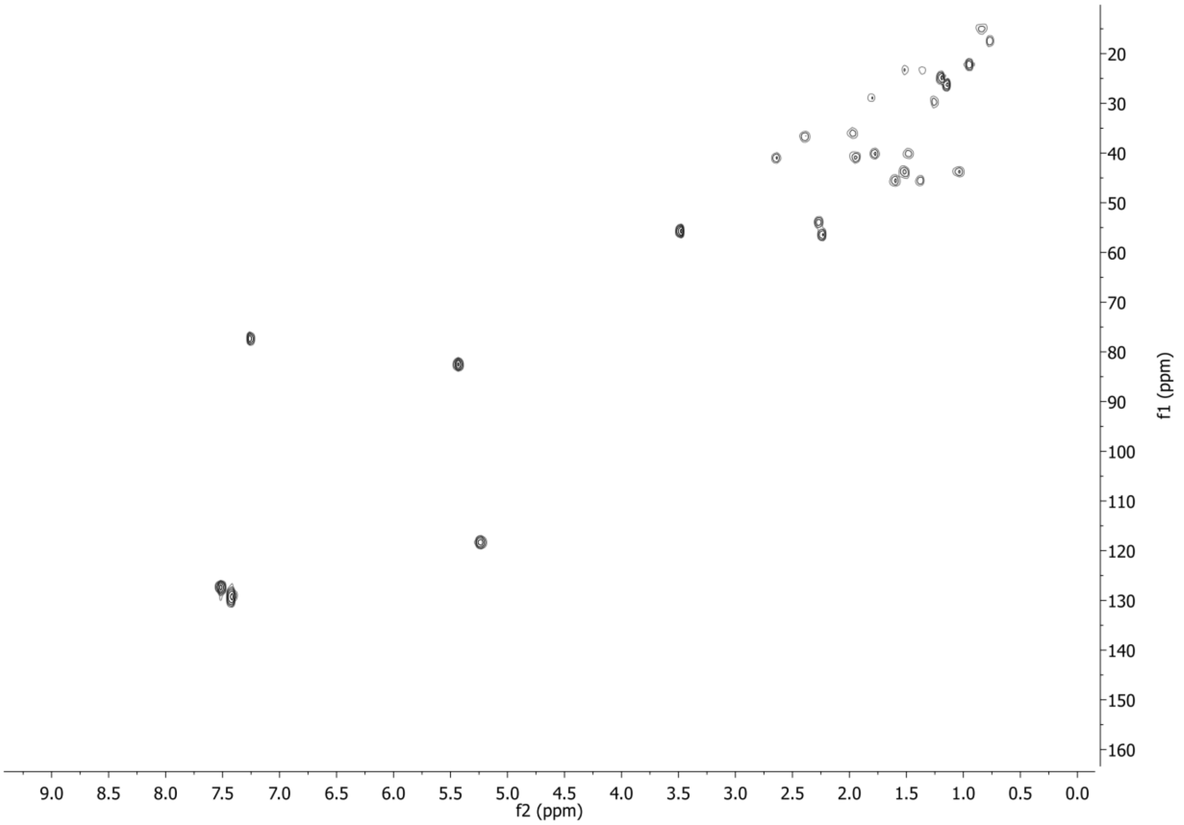

Figure S3n. NOESY NMR (500 MHz, $CDCl_3$) of the C-5 R-MTPA derivative of cyclooctat-9-en-5,7-diol.

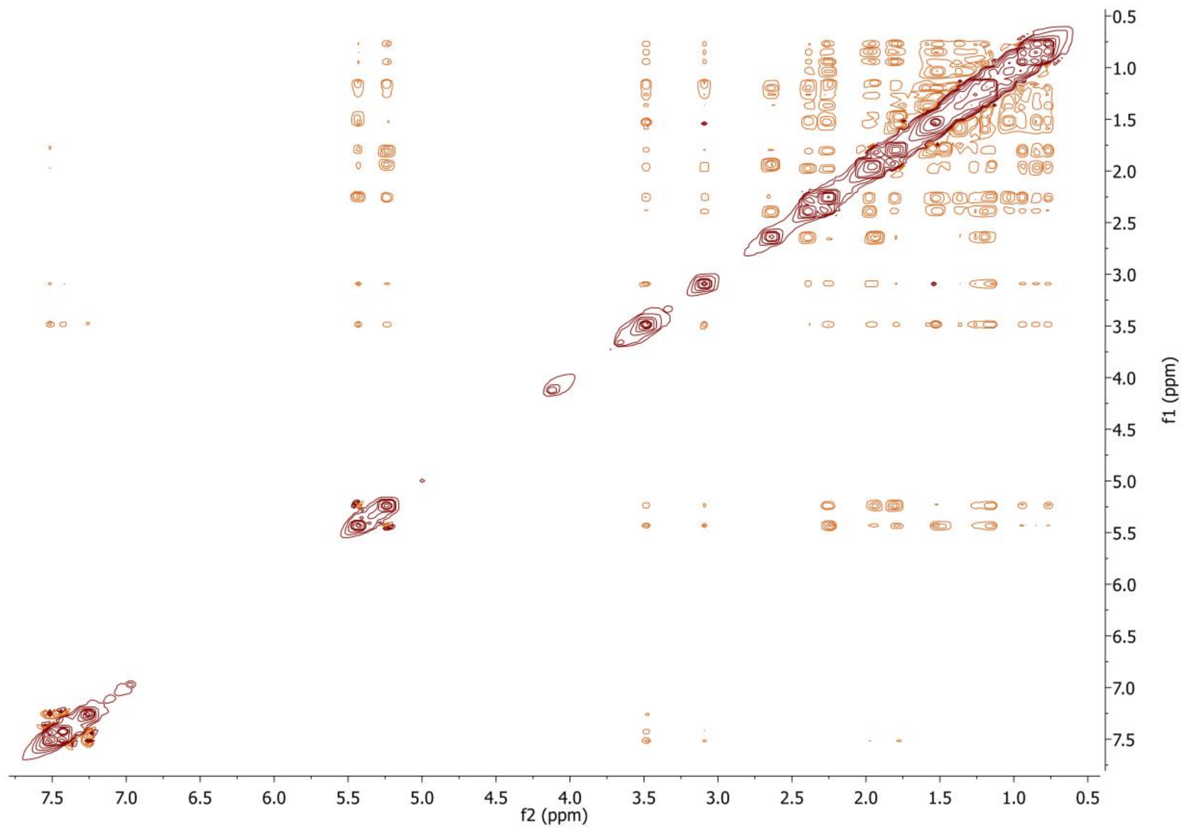

Figure S3o. ^{1}H NMR (500 MHz, $CDCl_3$) of the C-5 S-MTPA derivative of cyclooctat-9-en-5,7-diol.

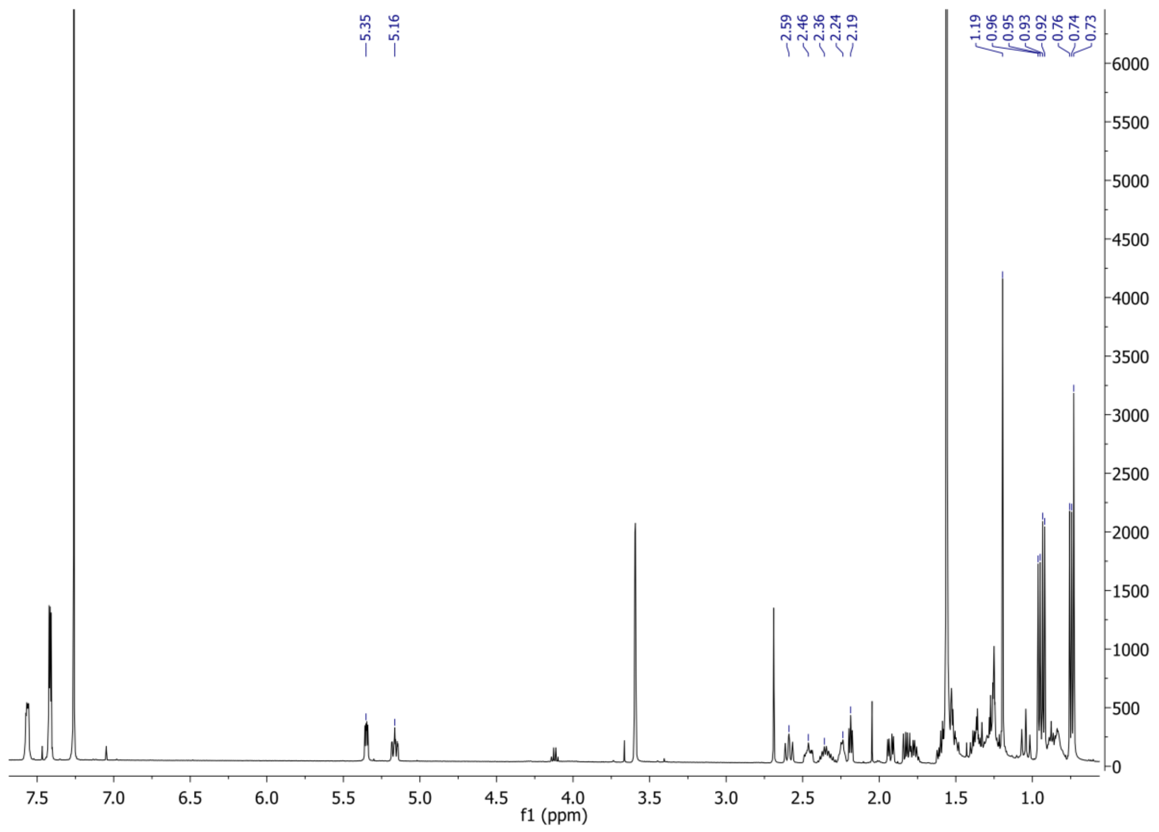

Figure S3p. ^{13}C NMR (125 MHz, $CDCl_3$) of the C-5 S-MTPA derivative of cyclooctat-9-en-5,7-diol.

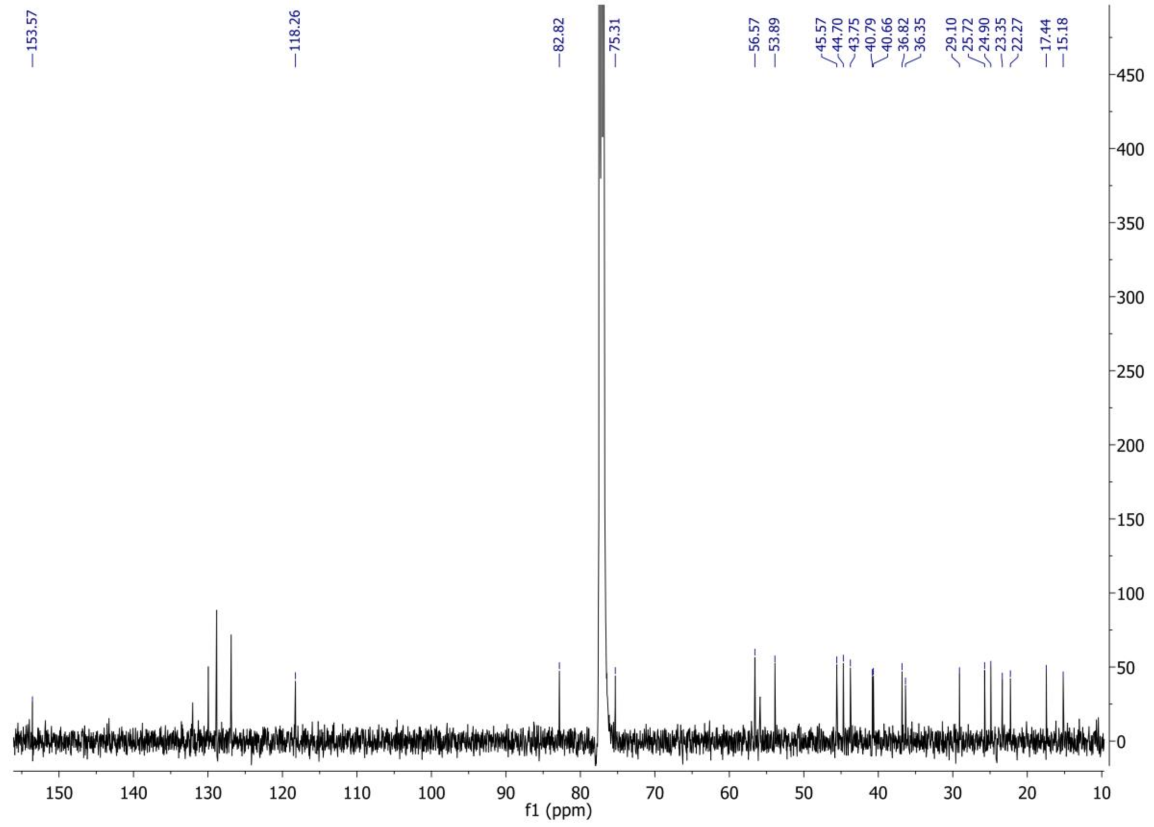

Figure S3q. COSY NMR (500 MHz, $CDCl_3$) of the C-5 S-MTPA derivative of cyclooctat-9-en-5,7-diol.

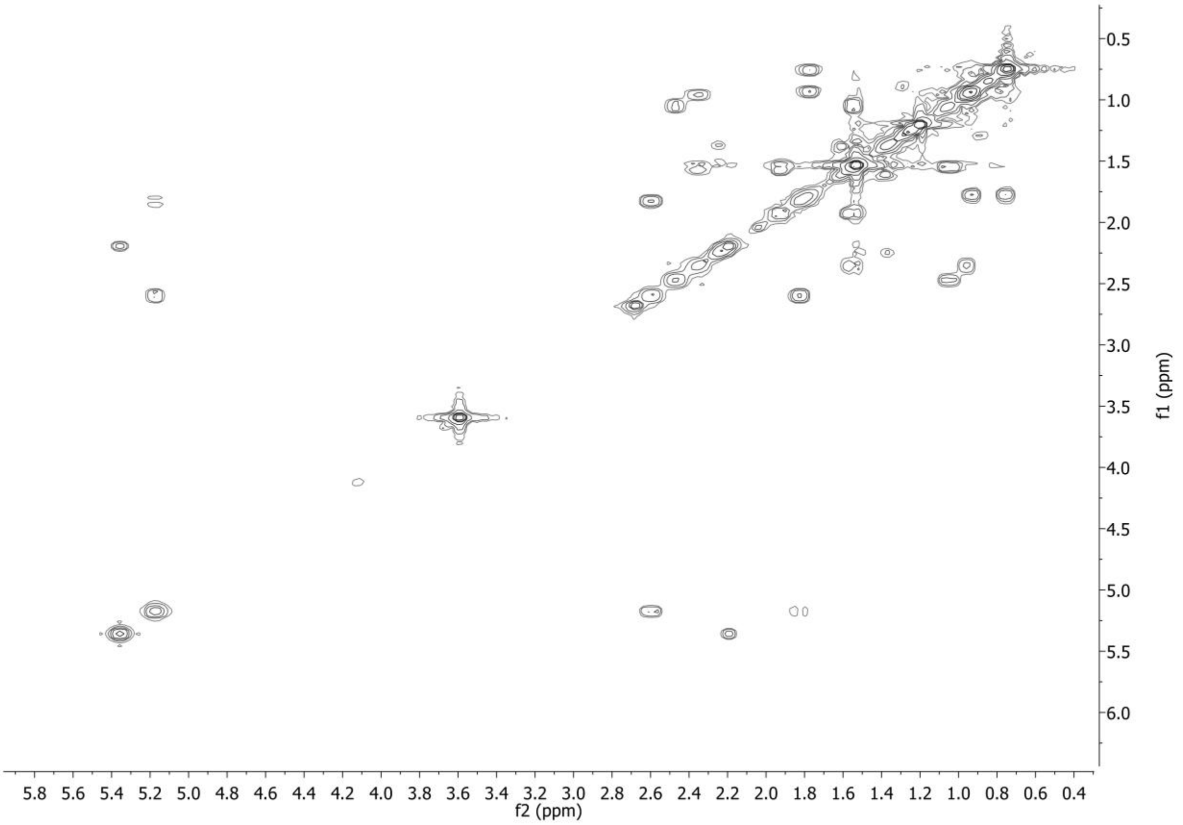

Figure S3r. HSQC NMR (500 MHz, $CDCl_3$) of the C-5 S-MTPA derivative of cyclooctat-9-en-5,7-diol.

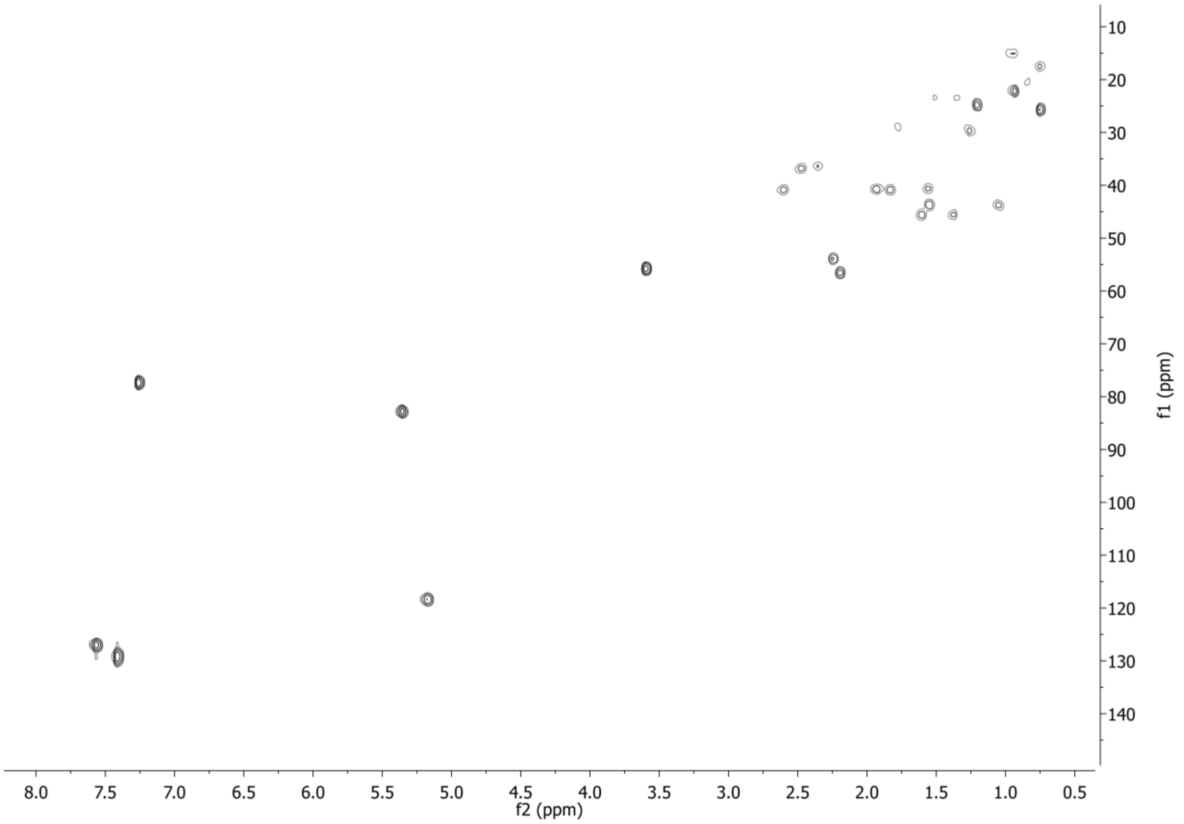

Figure S3s. NOESY NMR (500 MHz, $CDCl_3$) of the C-5 S-MTPA derivative of cyclooctat-9-en-5,7-diol.

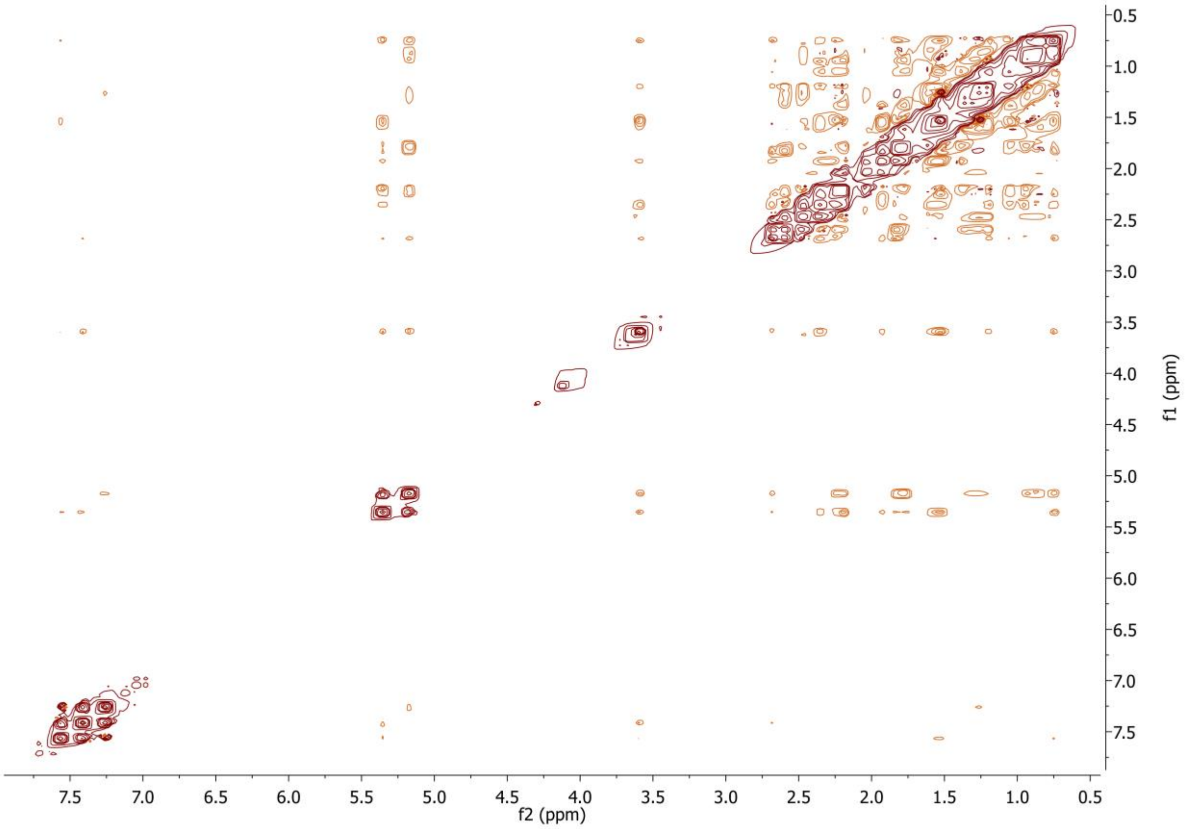

Chiral derivatizing and analysis

Figure S3t. ^{1}H NMR (500 MHz, $CDCl_3$) Overlay of the C-5 R/S-MTPA derivatives of cyclooctat-9-en-5,7-diol.

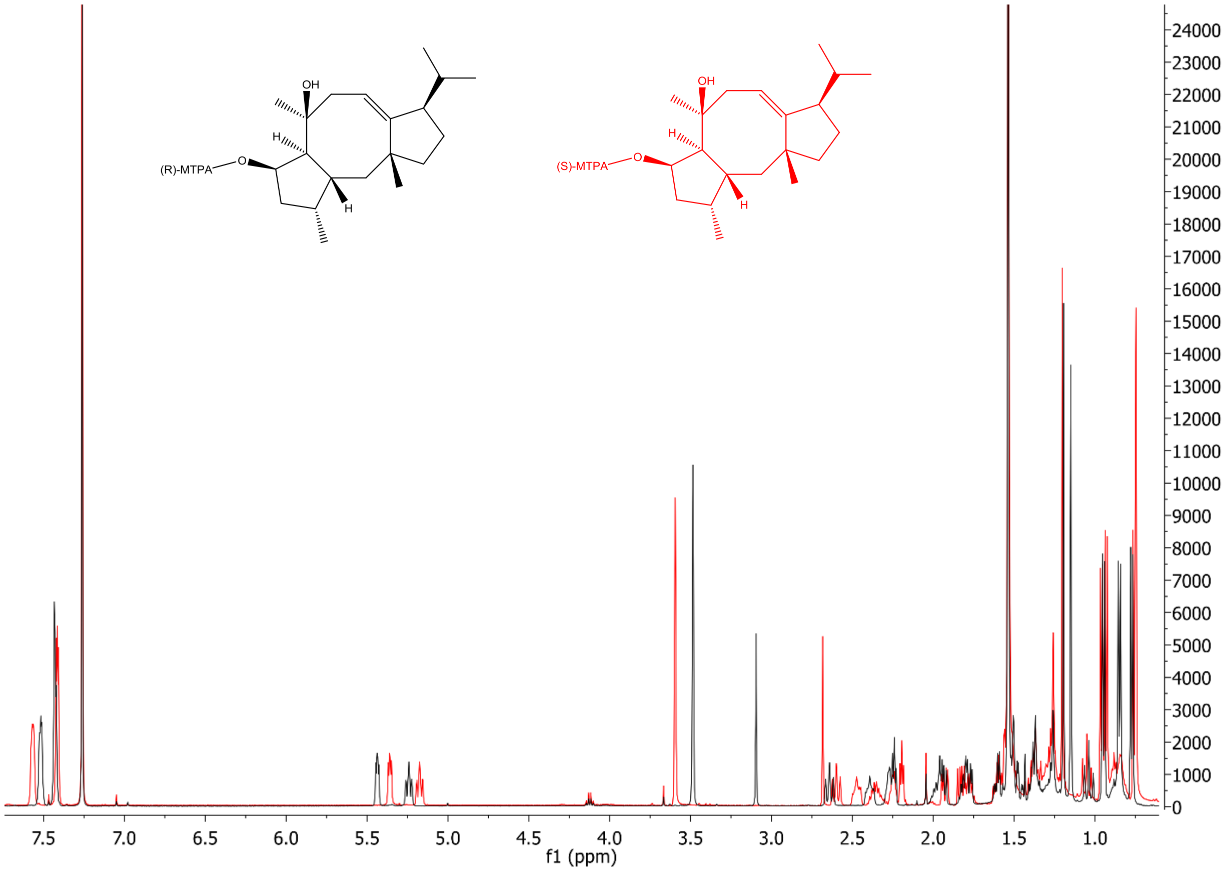

Table S4. Experimental ^{1}H ($CDCl_3$, 500 MHz, δ_H) and ^{13}C ($CDCl_3$, 125 MHz, δ_C) NMR data of the C-5 R-MTPA derivatives of cyclooctat-9-en-5,7-diol.

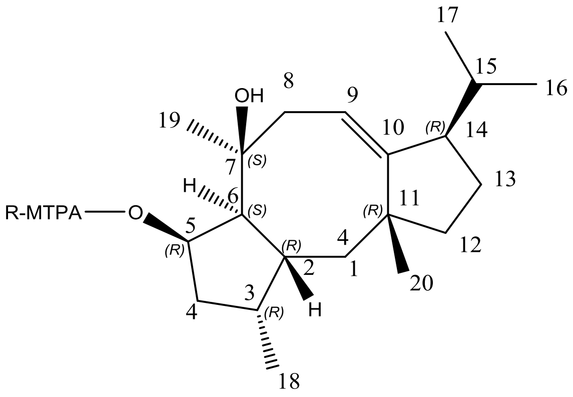

Position	δ_C	δ_H
**1**	43.82	1.04, 1.52
**2**	36.61	2.39
**3**	36.06	1.97
**4**	40.20	1.49, 1.78
**5**	82.58	5.43
**6**	56.45	2.24
**7**	75.51	
**8**	40.86	1.94, 2.64
**9**	118.25	5.24
**10**	153.63	
**11**	44.70	
**12**	45.58	1.38, 1.60
**13**	23.34	1.36, 1.51
**14**	53.91	2.27
**15**	29.09	1.26
**16**	17.42	0.77
**17**	22.28	0.95
**18**	15.05	0.84
**19**	26.31	1.14
**20**	24.92	1.19

Table S5. Experimental ^{1}H ($CDCl_3$, 500 MHz, δ_H) and ^{13}C ($CDCl_3$, 125 MHz, δ_C) NMR data of the C-5 S-MTPA derivatives of cyclooctat-9-en-5,7-diol.

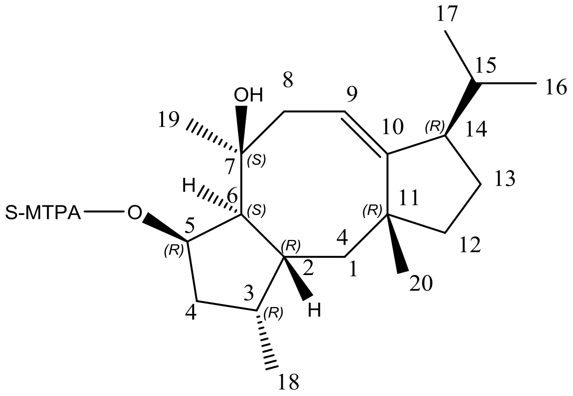

Position	δ_C	δ_H
**1**	43.75	1.04, 1.55
**2**	36.82	2.48
**3**	36.35	2.35
**4**	40.66	1.56, 1.93
**5**	82.82	5.36
**6**	56.57	2.19
**7**	75.31	
**8**	40.79	1.83, 2.60
**9**	118.26	5.17
**10**	153.57	
**11**	44.7	
**12**	45.57	1.37, 1.60
**13**	23.35	1.35, 1.51
**14**	53.89	2.25
**15**	29.10	1.26
**16**	17.44	0.75
**17**	22.27	0.93
**18**	15.18	0.95
**19**	25.72	0.75
**20**	24.9	1.21

Figure S4. Δδ (S – R) values (ppm) for the C-5 MTPA derivatives of cyclooctat-9-en-5,7-diol.

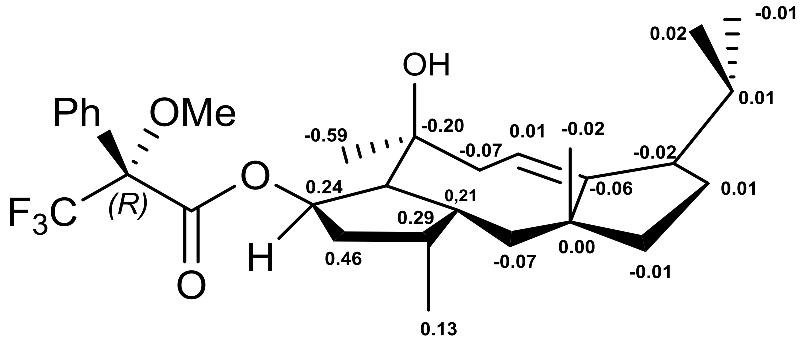

^{13}C-NMR Δδ (S – R) values (ppm)

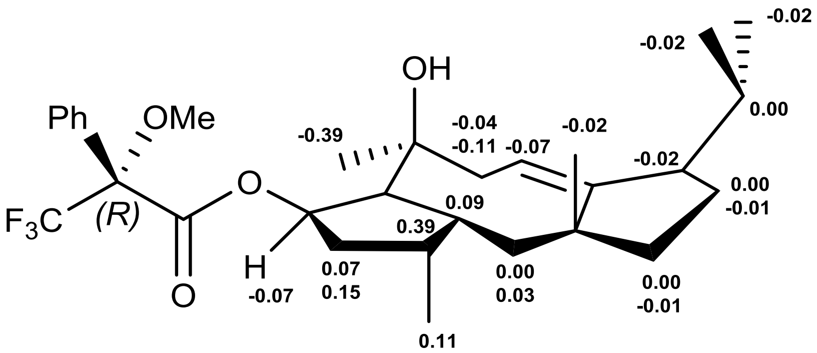

^{1}H-NMR Δδ (S – R) values (ppm)

Figure S5a. ^{1}H NMR (500 MHz, $CDCl_3$) of (-)-Casbene.

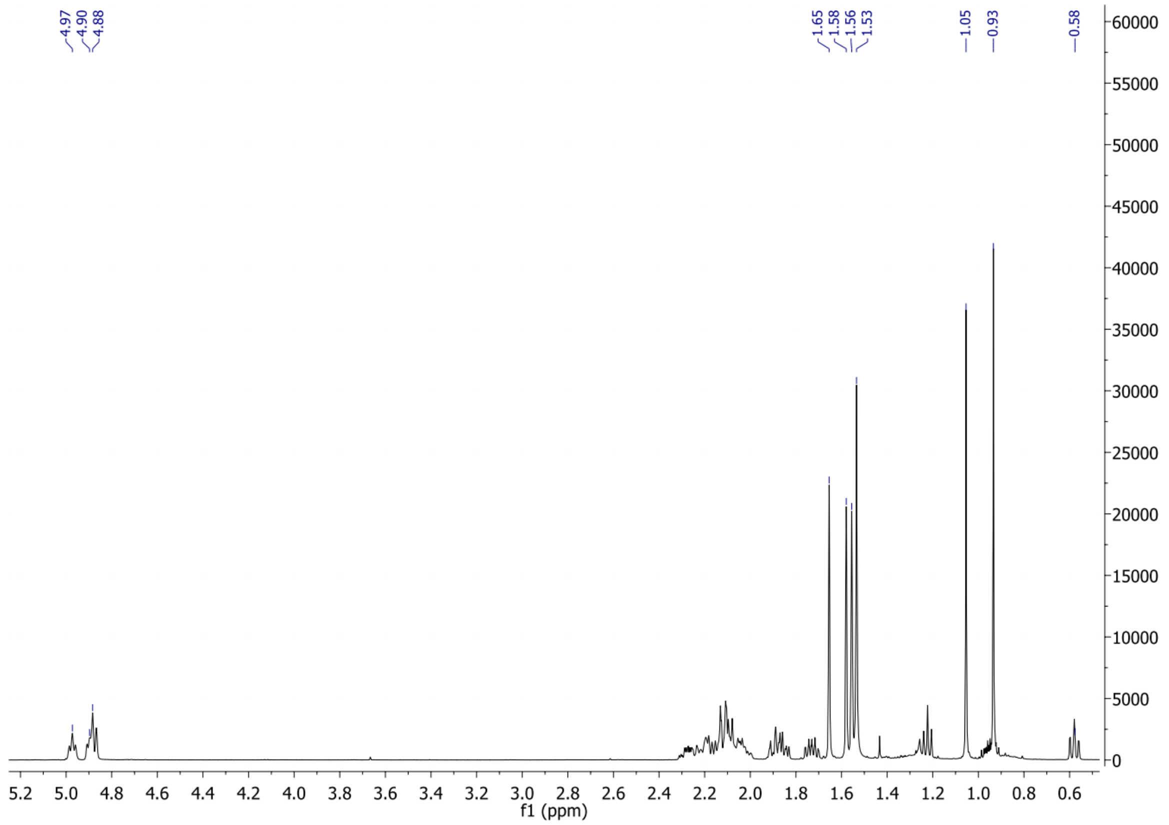

Figure S5b. ^{13}C NMR (125 MHz, $CDCl_3$) of (-)-Casbene.

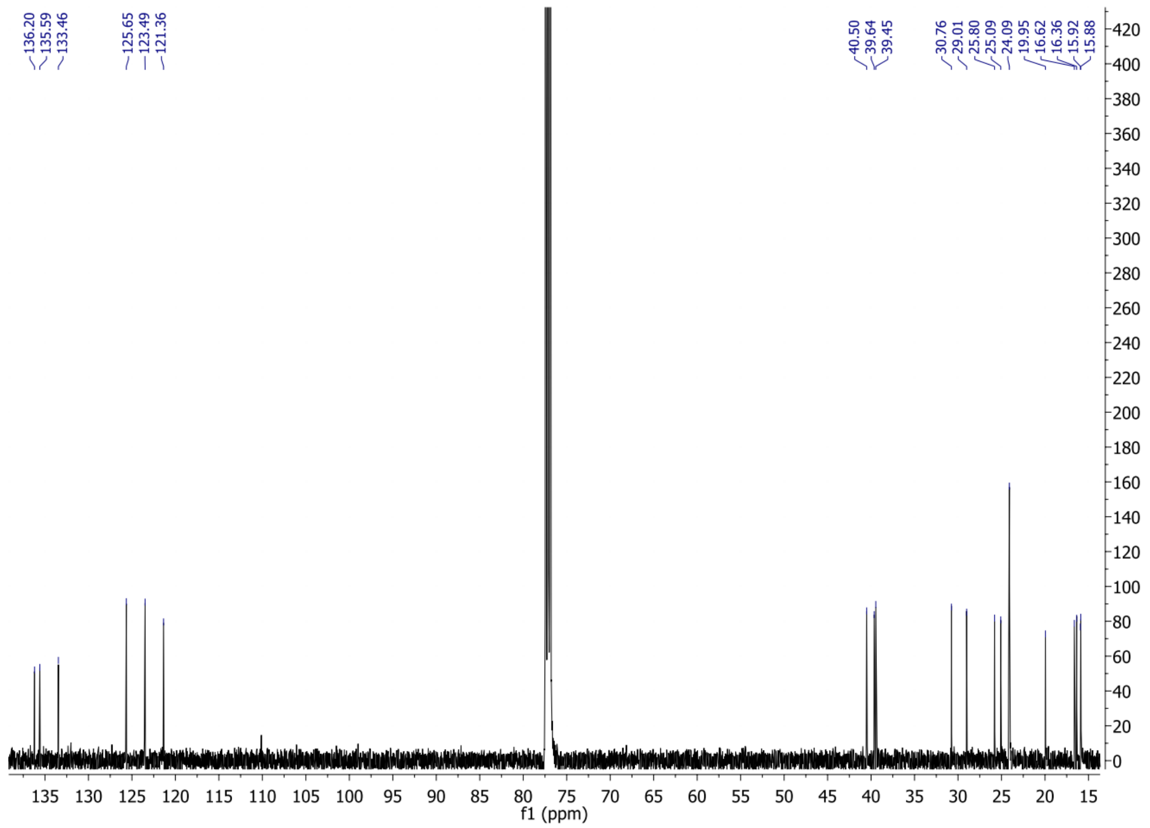

Table S6. Experimental ^{1}H ($CDCl_3$, 500 MHz, δ_H, J in Hz) and ^{13}C ($CDCl_3$, 125 MHz, δ_C) NMR data of sinularcasbane D.

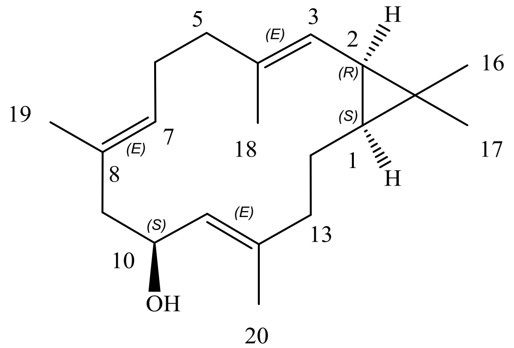

Position	δ_C	δ_H
**1**	30.80 (CH)	0.65 ddd (10.5, 8.7, 1.7)
**2**	25.97 (CH)	1.21 t (8.5)
**3**	121.41 (CH)	4.84 d (8.3)
**4**	135.89 (C)	
**5**	39.57 (CH_2)	2.19 (m)<br>2.06 (m)
**6**	25.09 (CH_2)	2.19 (m)<br>2.05 (m)
**7**	127.09 (CH)	4.87 ddd (6.7, 4.4, 1.2)
**8**	130.95 (C)	
**9**	48.15 (CH_2)	2.35 (m)<br>2.08 (m)
**10**	67.00 (CH)	4.52 ddd (10.7, 9.1, 3.2)
**11**	127.01 (CH)	4.98 dq (9.2, 1.4)
**12**	140.31 (C)	
**13**	39.70 (CH_2)	2.24 (m)<br>1.81 (m)
**14**	24.03 (CH_2)	1.68 (m)<br>1.13 (m)
**15**	20.16 (C)	
**16**	29.06 (CH_3)	1.07 (s)
**17**	15.74 (CH_3)	0.96 (s)
**18**	16.08 (CH_3)	1.62 (s)
**19**	17.25 (CH_3)	1.59 (s)
**20**	18.72 (CH_3)	1.66 (s)

Figure S6. COSY and HMBC correlations of sinularcasbane D.

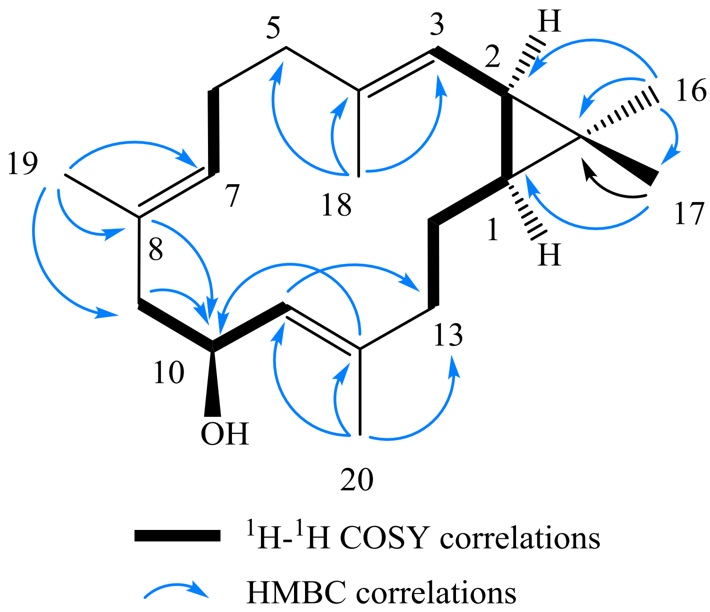

Figure S7. Illustrated NOESY correlations of sinularcasbane D. Modelling was done using molecular mechanics (MM2) calculations with ChemDraw Ultra Ver. 13.0 (CambridgeSoft).

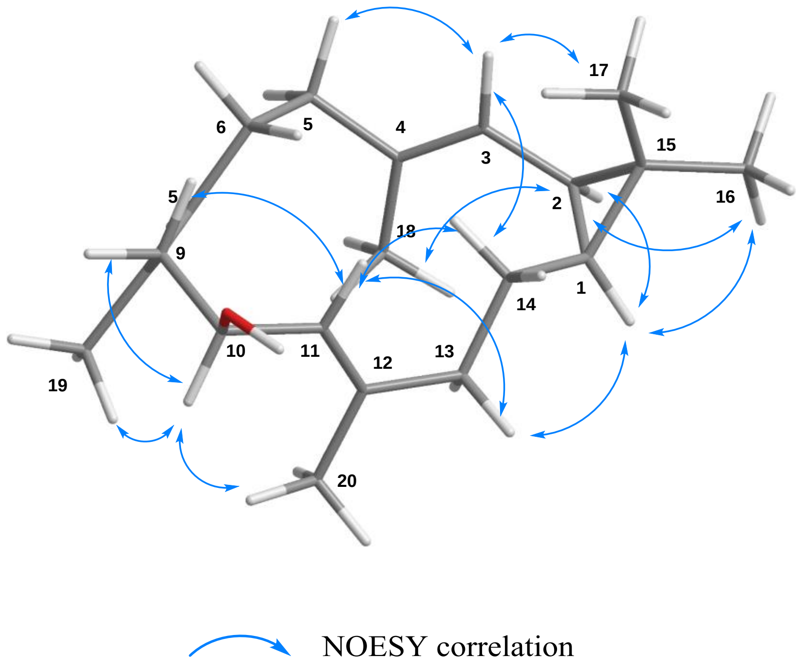

Figure S8a. ^{1}H NMR (500 MHz, $CDCl_3$) of sinularcasbane D.

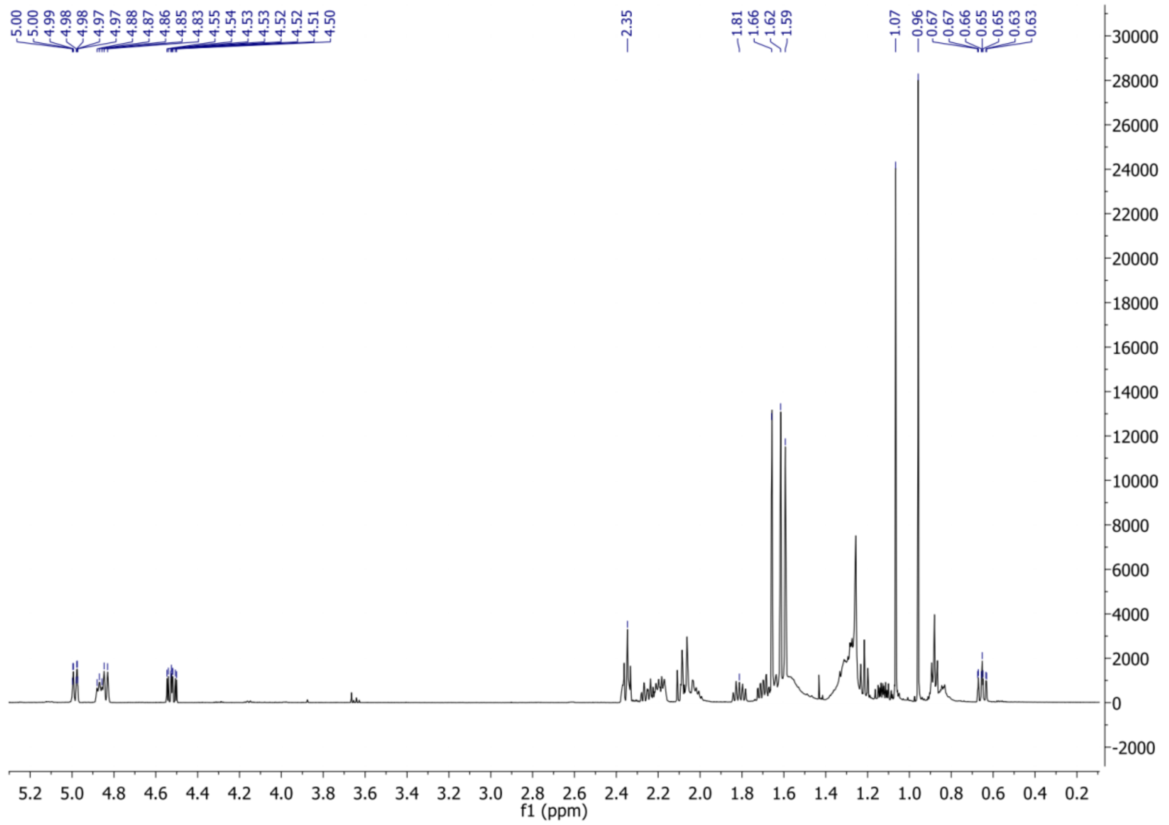

Figure S8b. ^1H NMR (500 MHz, $CDCl_3$) of sinularcasbane D.

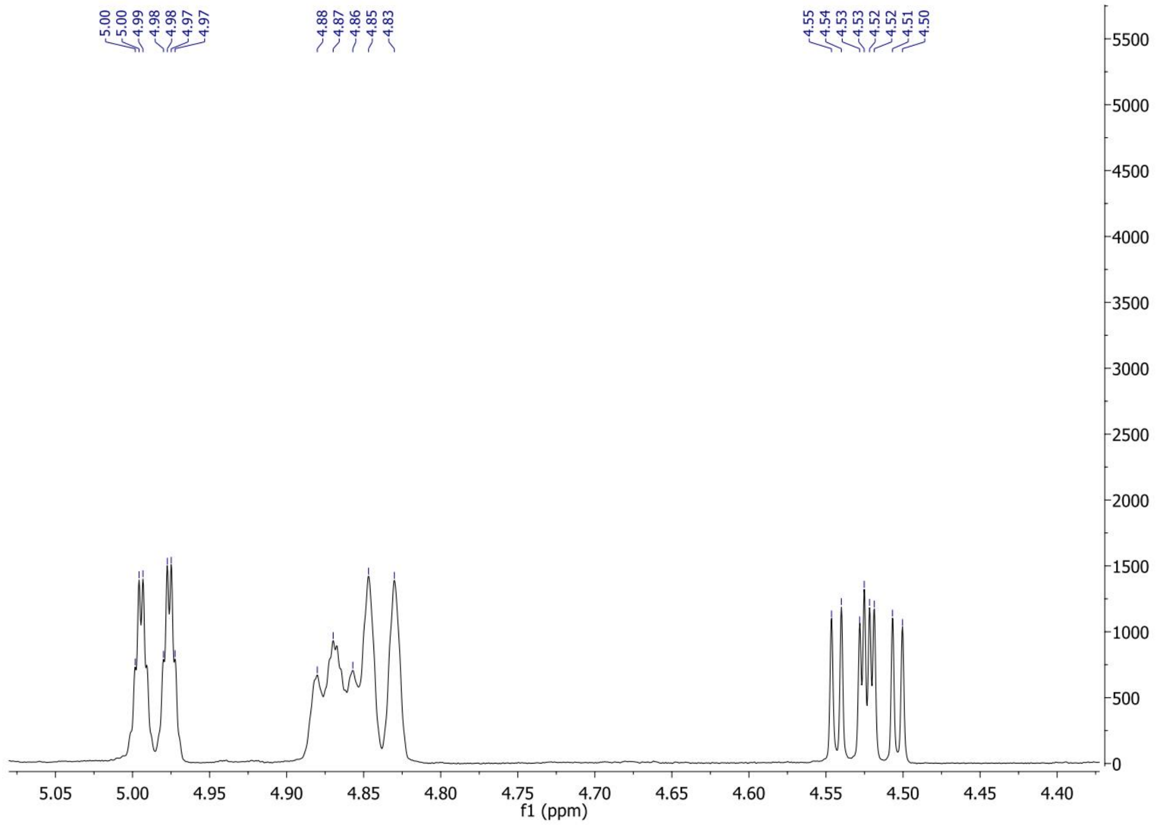

Figure S8c. ^{13}C NMR (125 MHz, $CDCl_3$) of sinularcasbane D.

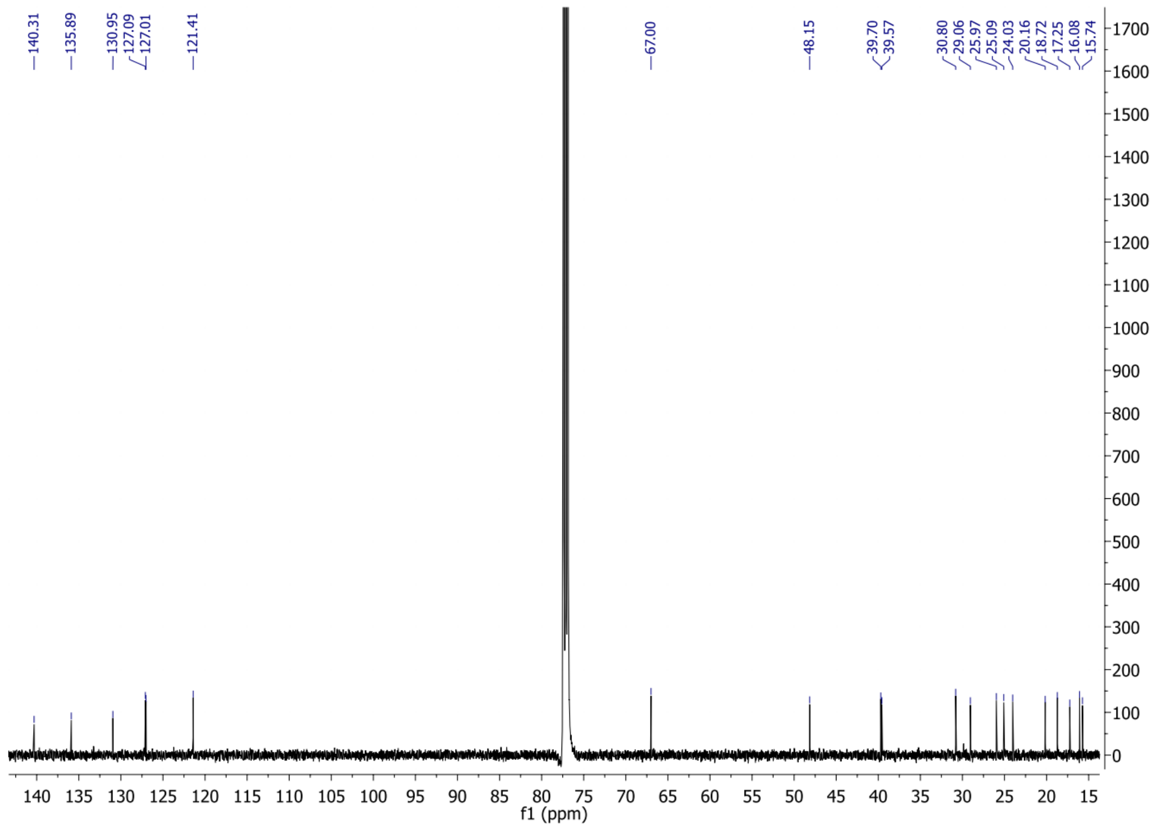

Figure S8d. COSY NMR (500 MHz, $CDCl_3$) of sinularcasbane D.

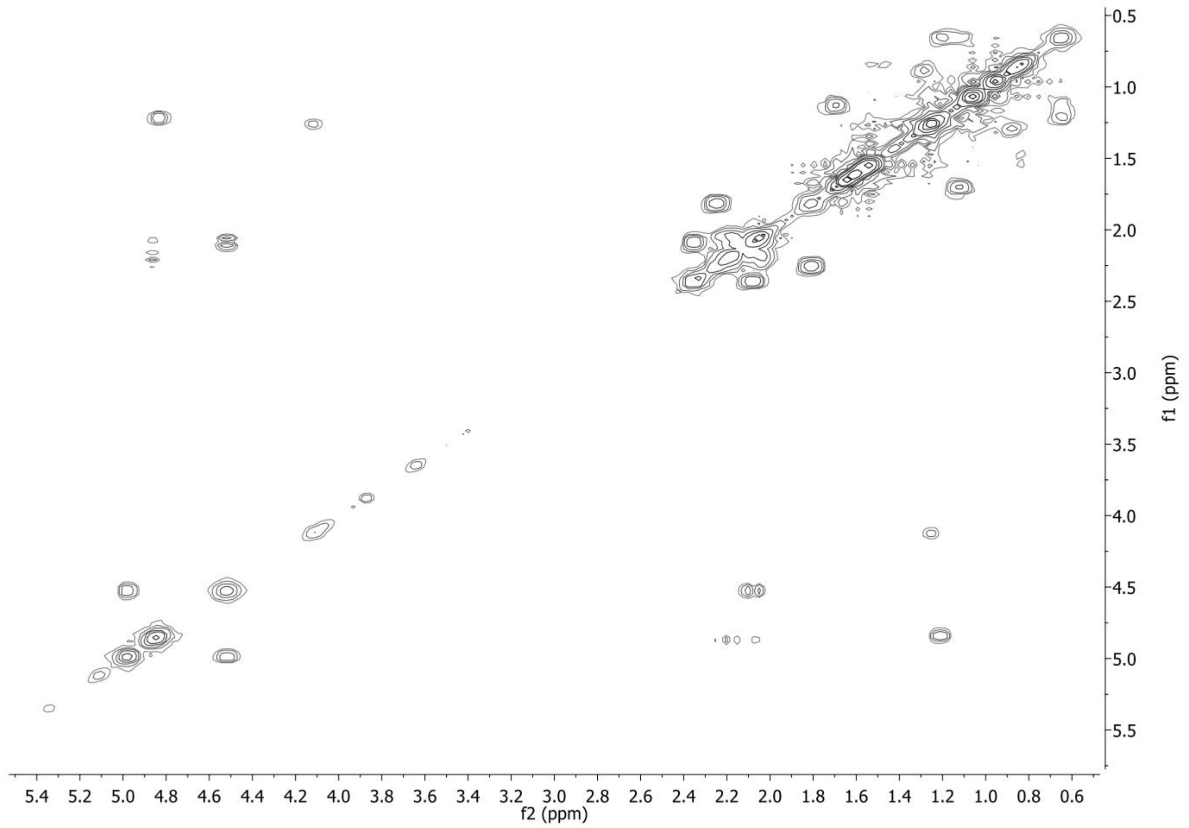

Figure S8e. HSQC NMR (500 MHz, $CDCl_3$) of sinularcasbane D.

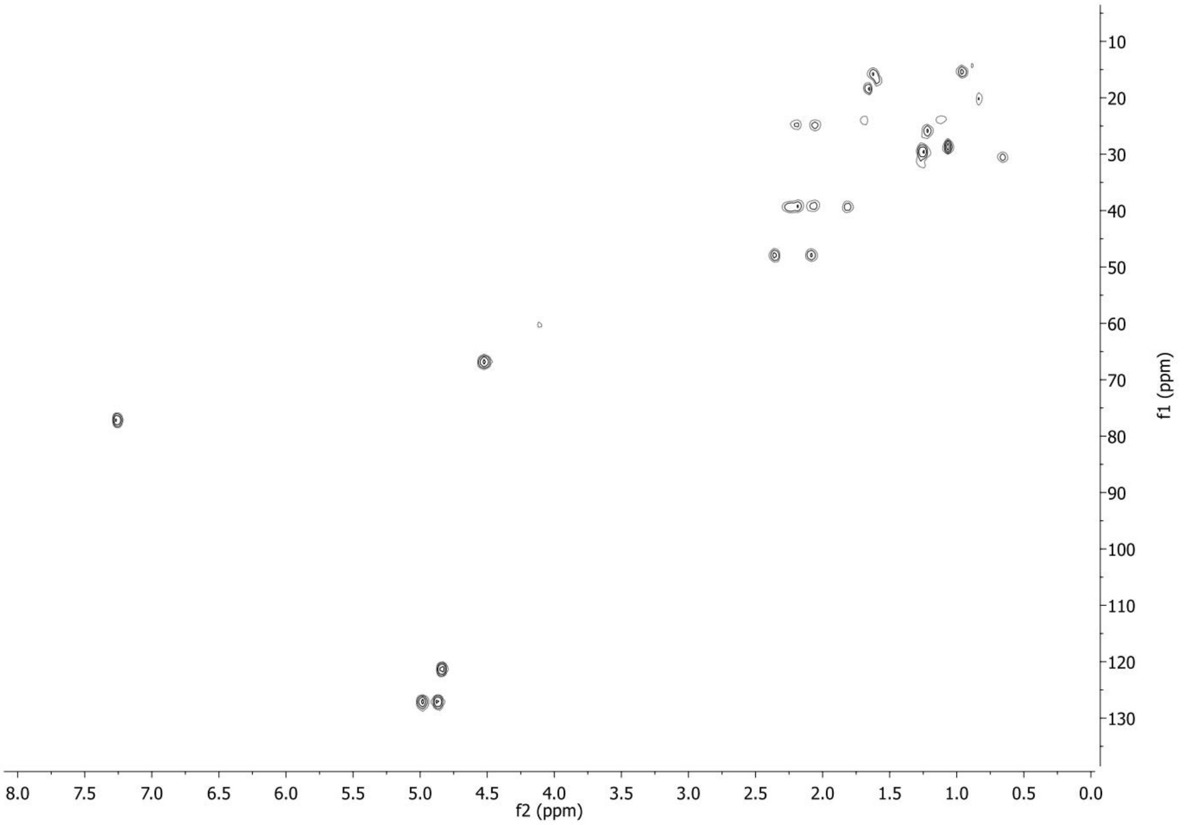

Figure S8f. HMBC NMR (500 MHz, $CDCl_3$) of sinularcasbane D.

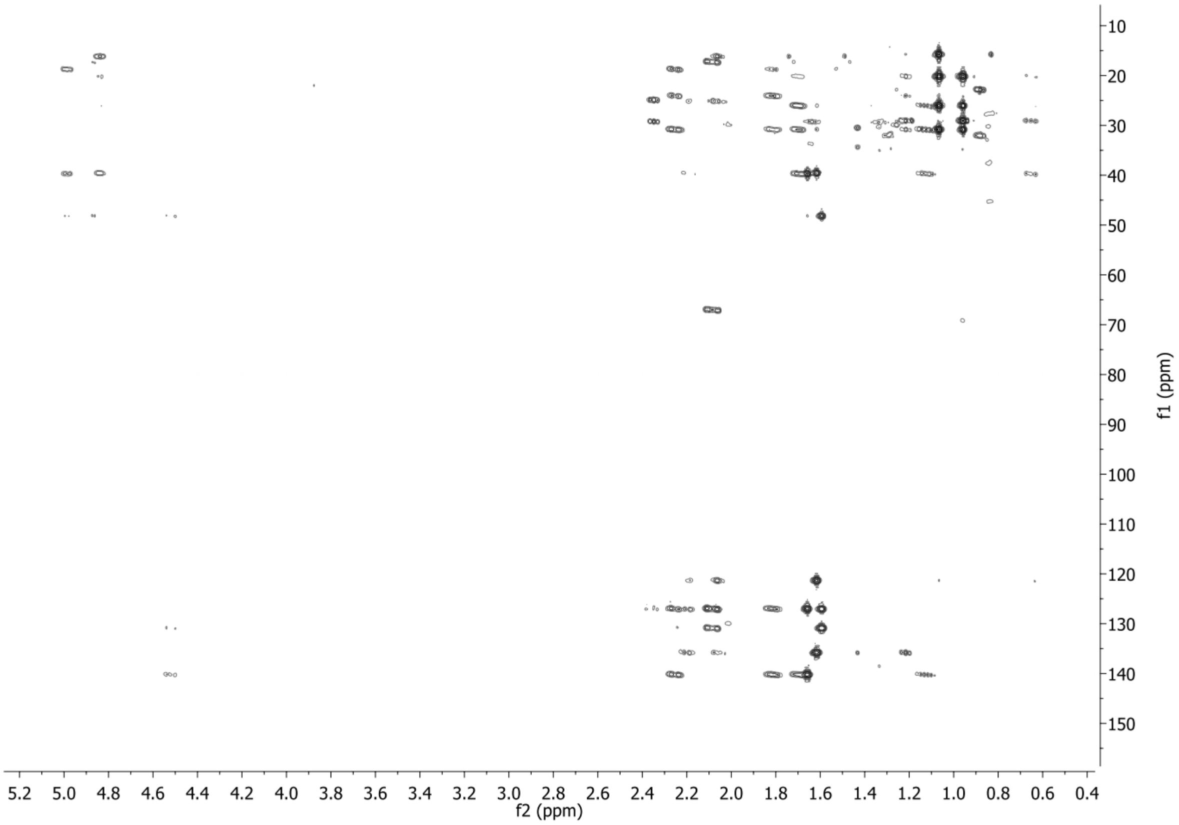

Figure S8g. NOESY NMR (500 MHz, $CDCl_3$) of sinularcasbane D.

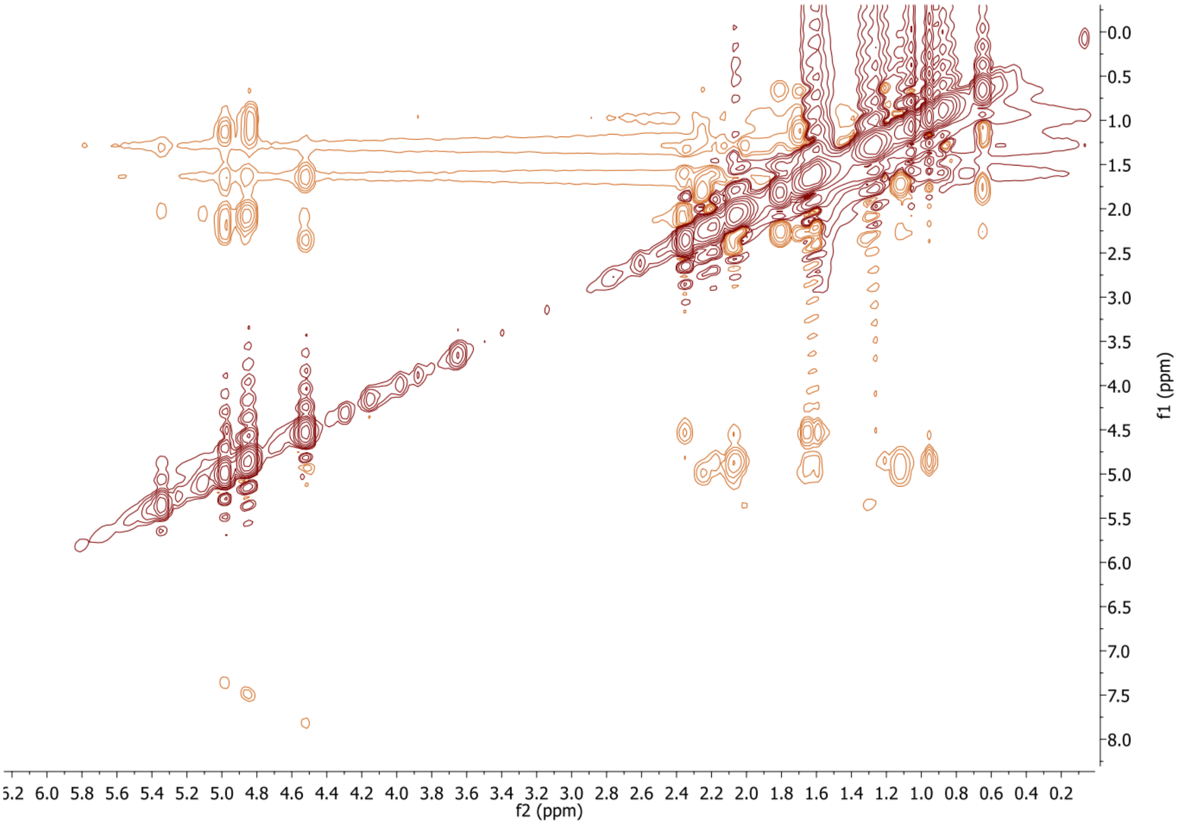

4. CD-Spectroscopy

Figure S9. Circular dichroism spectroscopy of cyclooctat-9-en-7-ol (**a**), cyclooctat-9-en-5-7-diol (**b**), cyclooctatin (**c**).

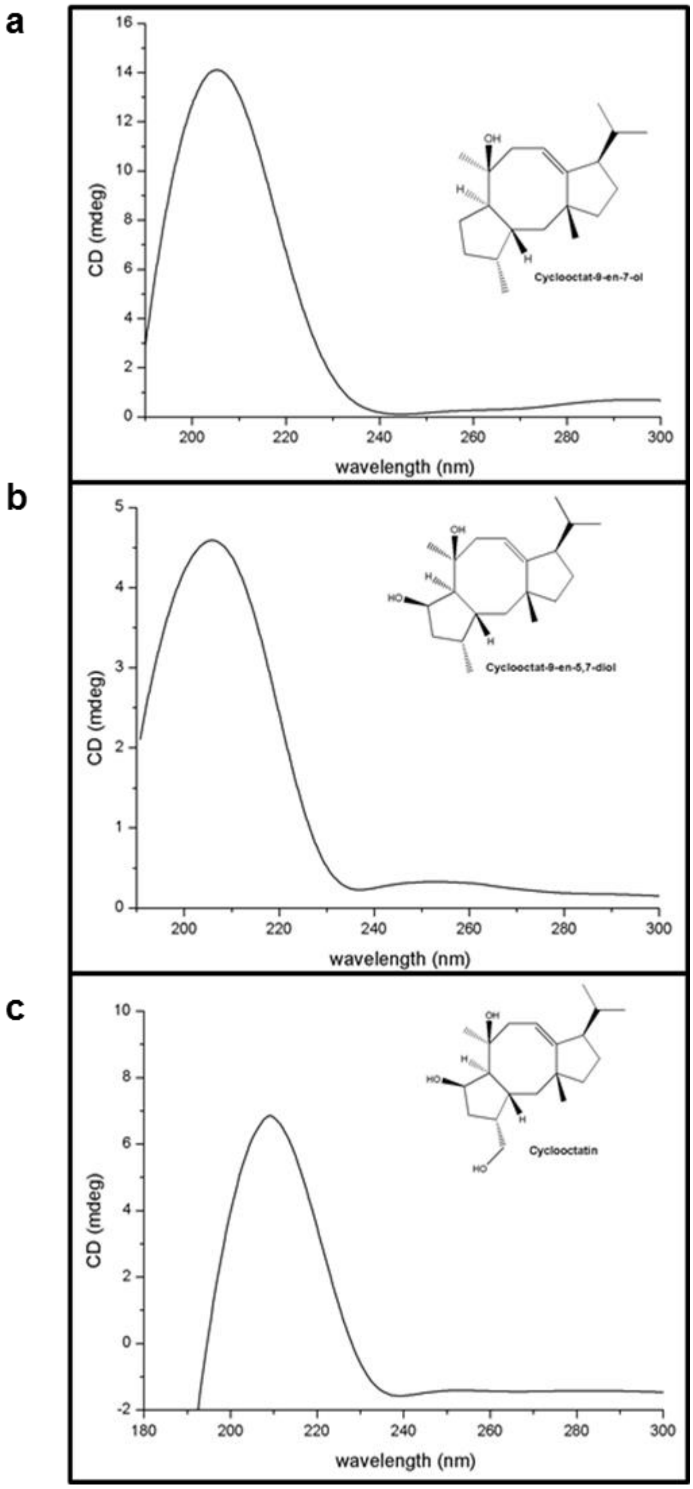

5. Mass spectrometry

Figure S10a. Mass spectrum of silylated cyclooctat-9-en-7-ol (RT: 21:16), recorded on a Trace GC Ultra with DSQII (Thermo Scientific), m/z was analyzed from [50-650]. m/z $C_{23}H_{42}OSi$ calculated. 362.30.

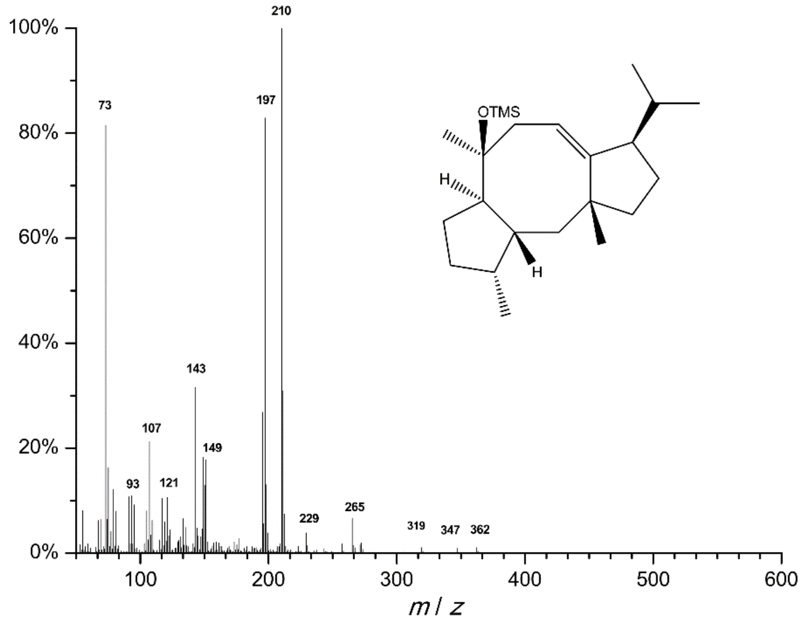

Figure S10b. Mass spectrum of silylated cyclooctat-9-en-5,7-diol (RT: 22:94), recorded on a Trace GC Ultra with DSQII (Thermo Scientific), m/z was analyzed from [50-650]. *m/z* $C_{26}H_{50}O_2Si_2$ calculated 450.33.

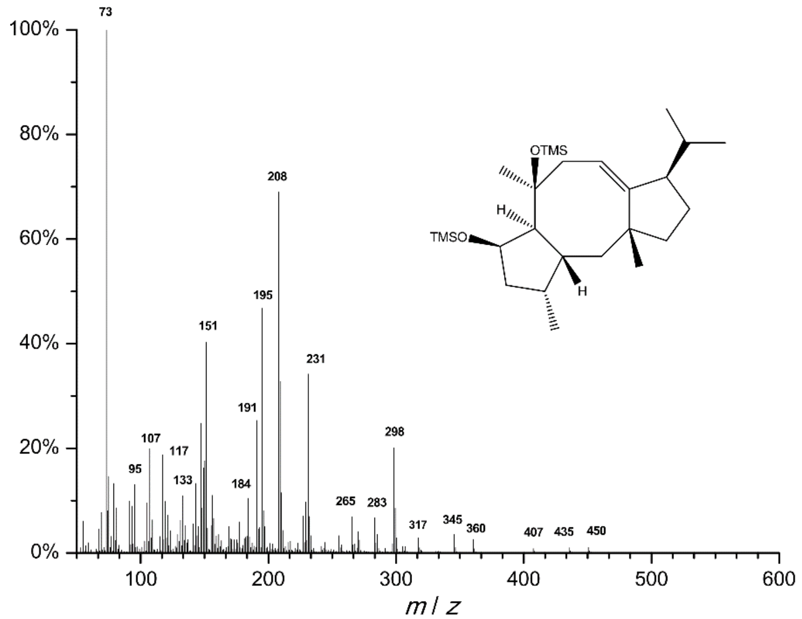

Figure S10c. Mass spectrum of silylated cyclooctatin (RT: 24:40), recorded on a Trace GC Ultra with DSQII (Thermo Scientific), m/z was analyzed from [50-650]. *m/z* $C_{28}H_{56}O_3Si_3$ calculated 524.35.

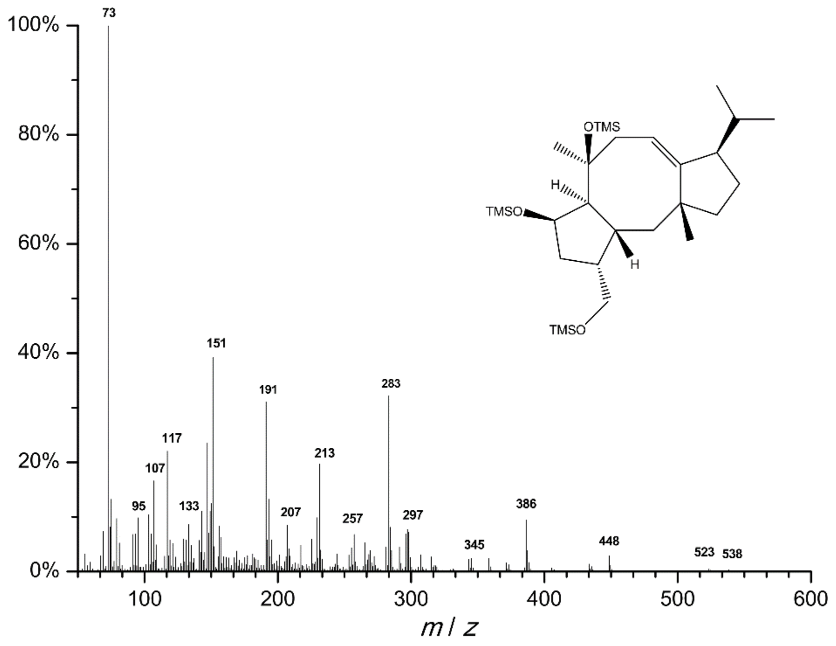

Figure S10f. Mass spectrum of sinularcasbane D (RT: 21:89), recorded on a Trace GC Ultra with DSQII (Thermo Scientific), m/z was analyzed from [50-650]. *m/z* $C_{20}H_{32}O$ calculated 288.24.

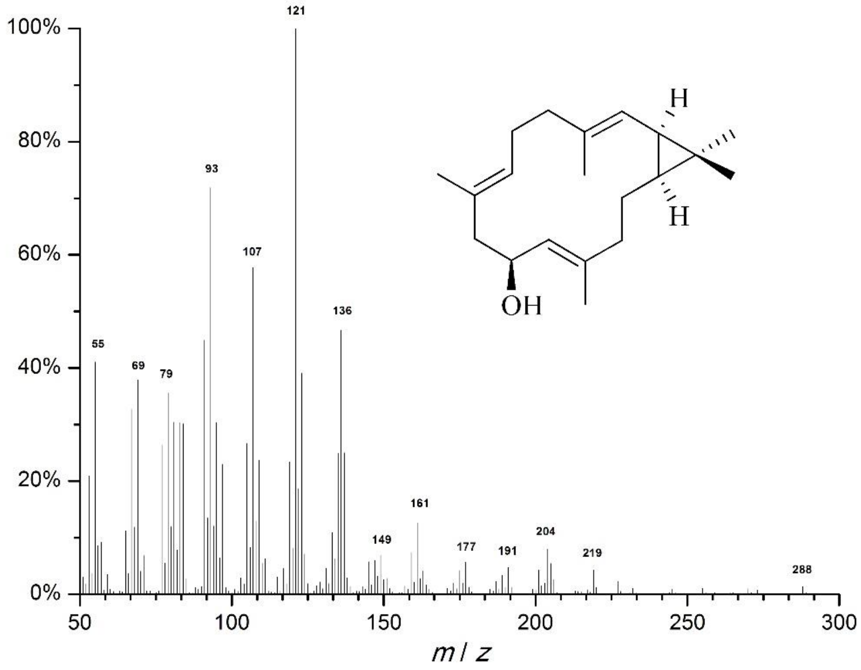

Figure S11. High resolution electron spray ionization of cyclooctat-9-en-7-ol (**a**) *m/z* ([M–H_2O]$^+$ 272.2568 calculated $C_{20}H_{32}$ 272.2504, cyclooctat-9-en-5-7-diol (**b**) *m/z* ([M–H_2O]$^+$ 272.2450 calculated $C_{20}H_{32}O$ 288.2453, cyclooctatin (**c**) *m/z* ([M–H_2O]$^+$ 304.2415 calculated $C_{20}H_{32}O_2$ 304.2402.

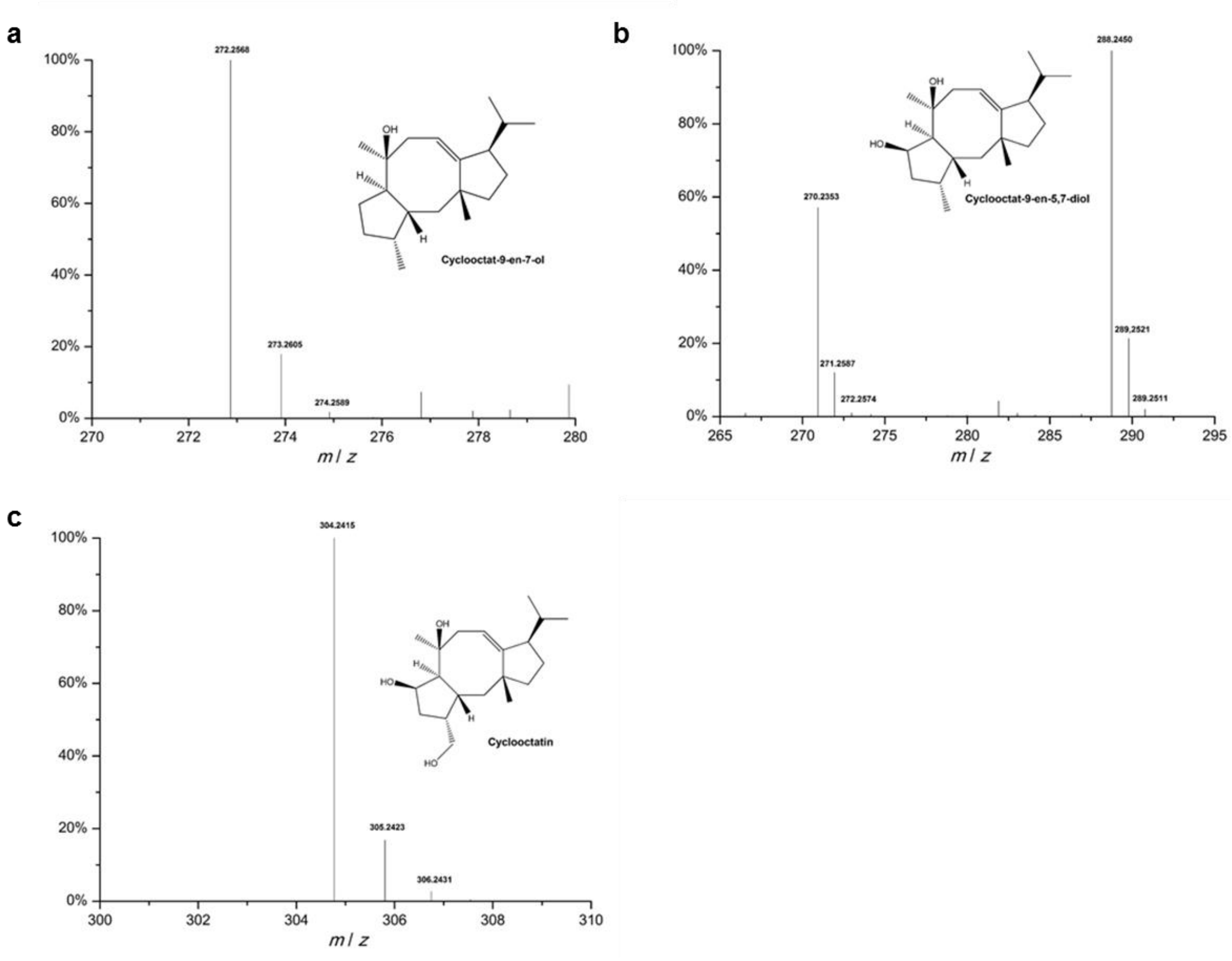

Figure S12. GC-MS spectra of a 72h shake flask culture of (-)-casbene and hydroxylation to sinularcasbane D by CotB3 on a Trace GC Ultra with DSQII (Thermo Scientific). 20 % of (-)-casbene were hydroxylated to sinularcasbane D.

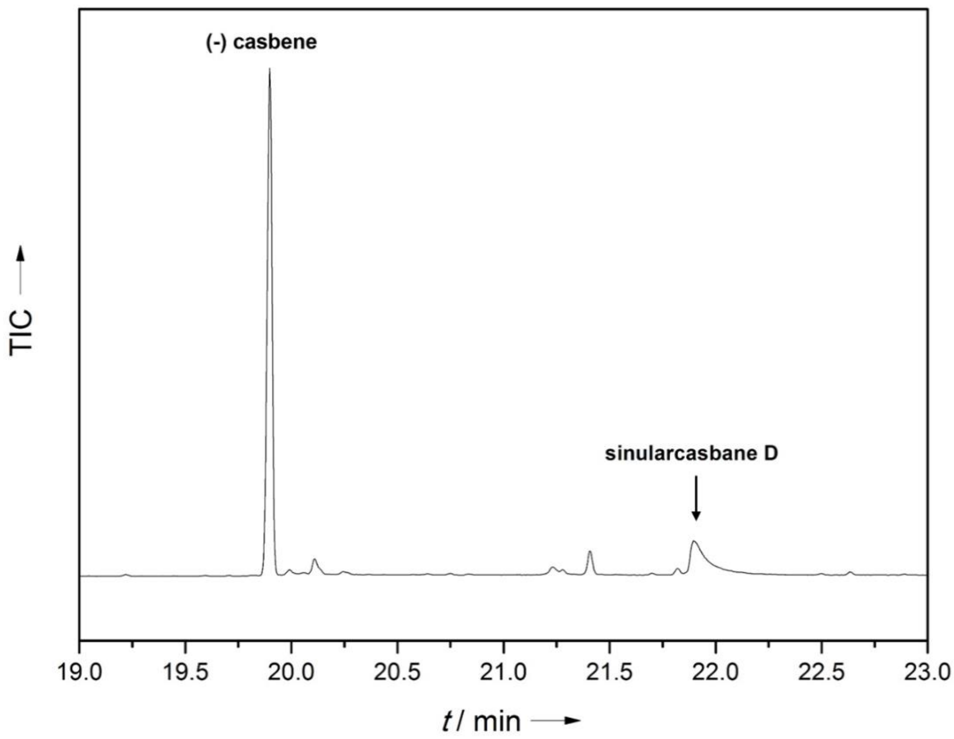

6. Genes

Figure S13. Gene of 1-deoxy-D-xylulose 5-phosphate synthase (dxs).

CCATGGATGAGTTTTGATATTGCCAAATACCCGACCCTGGCACTGGTCGACTCCACCCAGGAGTT
ACGACTGTTGCCGAAAGAGAGTTTACCGAAACTCTGCGACGAACTGCGCCGCTATTTACTCGACA
GCGTGAGCCGTTCCAGCGGGCACTTCGCCTCCGGGCTGGGCACGGTCGAACTGACCGTGGCGC
TGCACTATGTCTACAACACCCCGTTTGACCAATTGATTTGGGATGTGGGGCATCAGGCTTATCCG
CATAAAATTTTGACCGGACGCCGCGACAAAATCGGCACCATCCGTCAGAAAGGCGGCCTGCACC
CGTTCCCGTGGCGCGGCGAAAGCGAATATGACGTATTAAGCGTCGGGCATTCATCAACCTCCAT
CAGTGCCGGAATTGGTATTGCGGTTGCTGCCGAGAAAGAAGGCAAAAATCGCCGCACCGTCTGT
GTCATTGGCGATGGCGCGATTACCGCTGGCATGGCGTTTGAAGCGATGAATCACGCGGGCGATA
TCCGTCCTGATATGCTGGTGGTCCTCAACGACAATGAAATGTCGATTTCCGAAAATGTCGGCGCG
CTCAATAACCATCTGGCACAGCTGCTTTCCGGTAAGCTTTACTCTTCGCTGCGCGAAGGCGGGAA
AAAAGTTTTCTCTGGCGTTCCGCCAATTAAAGAGCTGCTCAAACGTACCGAAGAACATATTAAAGG
CATGGTAGTGCCTGGCACGTTGTTTGAAGAGCTGGGCTTTAACTACATCGGCCCGGTTGACGGT
CACGATGTGCTGGGGCTTATCACCACGCTGAAGAACATGCGCGACCTGAAAGGCCCGCAGTTCC
TGCATATCATGACCAAAAAAGGTCGTGGTTATGAACCGGCAGAAAAAGACCCCATCACTTTCCAC
GCCGTGCCTAAATTTGATCCCTCCAGCGGTTGTTTGCCGAAAAGTAGCGGCGGTTTGCCGAGCT
ATTCAAAAATCTTTGGCGACTGGTTGTGCGAAACGGCAGCGAAAGACAACAAGCTGATGGCGATT
ACTCCGGCGATGCGTGAAGGTTCCGGCATGGTCGAGTTTTCACGTAAATTCCCGGATCGTTACTT
CGACGTGGCAATCGCCGAGCAACACGCGGTGACCTTTGCCGCCGGTCTGGCGATTGGTGGGTA
CAAACCCATTGTCGCGATTTACTCCACTTTCCTGCAACGCGCCTATGATCAGGTGCTGCATGACG
TGGCGATTCAAAAGCTCCCGGTCCTGTTCGCCATCGACCGCGCGGGCATTGTTGGTGCTGACGG
TCAAACCCATCAGGGCGCTTTTGACCTCTCTTACCTGCGCTGTATACCGGAAATGGTCATTATGA
CCCCGAGCGATGAAAACGAATGTCGCCAGATGCTCTATACCGGCTATCACTATAACGACGGCCC
GTCCGCGGTGCGCTACCCGCGCGGTAACGCGGTTGGCGTGGAACTGACGCCGCTGGAAAAACT
GCCAATTGGCAAAGGCATTGTGAAGCGTCGTGGCGAGAAACTGGCGATCCTTAACTTTGGTACG
CTGATGCCAGACGCGGCGAAAGTCGCTGAATCGCTGAACGCTACGCTGGTCGATATGCGTTTTG
TGAAACCGCTTGATGAAGCGTTAATTCTGGAAATGGCCGCCAGCCATGAAGCGCTGGTCACCGT
AGAAGAAAACGCCATTATGGGCGGCGCAGGCAGCGGCGTGAACGAAGTGCTAATGGCCCATCG
TAAACCAGTACCCGTGCTGAACATTGGCCTGCCTGACTTCTTTATTCCACAAGGAACTCAGGAAG
AAATGCGCGCCGAACTCGGCCTCGATGCCGCCGGTATGGAAGCCAAAATCAAGGCCTGGCTGG
CATAAGAATTC

Figure S14. Gene of 1-deoxy-D-xylulose 5-phosphate reductoisomerase (*dxp*).

CATATGAAGCAACTCACCATTCTGGGCTCGACCGGCTCGATTGGTTGCAGCACGCTGGACGTGG
TGCGCCATAATCCCGAACACTTCCGCGTAGTTGCGCTGGTGGCAGGCAAAAATGTCACTCGCAT
GGTAGAACAGTGCCTGGAATTCTCTCCCCGCTATGCCGTAATGGACGATGAAGCGAGTGCGAAA
CTTCTTAAAACGATGCTACAGCAACAGGGTAGCCGCACCGAAGTCTTAAGTGGGCAACAAGCCG
CTTGCGATATGGCAGCGCTTGAGGATGTTGATCAGGTGATGGCAGCCATTGTTGGCGCTGCTGG
GCTGTTACCTACGCTTGCTGCGATCCGCGCGGGTAAAACCATTTTGCTGGCCAATAAAGAATCAC
TGGTTACCTGCGGACGTCTGTTTATGGACGCCGTAAAGCAGAGCAAAGCGCAATTGTTACCGGT
CGATAGCGAACATAACGCCATTTTTCAGAGTTTACCGCAACCTATCCAGCATAATCTGGGATACG
CTGACCTTGAGCAAAATGGCGTGGTGTCCATTTTACTTACCGGGTCTGGTGGCCCTTTCCGTGAG
ACGCCATTGCGCGATTTGGCAACAATGACGCCGGATCAAGCCTGCCGTCATCCGAACTGGTCGA
TGGGGCGTAAAATTTCTGTCGATTCGGCTACCATGATGAACAAAGGTCTGGAATACATTGAAGCG
CGTTGGCTGTTTAACGCCAGCGCCAGCCAGATGGAAGTGCTGATTCACCCGCAGTCAGTGATTC
ACTCAATGGTGCGCTATCAGGACGGCAGTGTTCTGGCGCAGCTGGGGGAACCGGATATGCGTAC
GCCAATTGCCCACACCATGGCATGGCCGAATCGCGTGAACTCTGGCGTGAAGCCGCTCGATTTT
TGCAAACTAAGTGCGTTGACATTTGCCGCACCGGATTATGATCGTTATCCATGCCTGAAACTGGC
GATGGAGGCGTTCGAACAAGGCCAGGCAGCGACGACAGCATTGAATGCCGCAAACGAAATCACC
GTTGCTGCTTTTCTTGCGCAACAAATCCGCTTTACGGATATCGCTGCGTTGAATTTATCCGTACTG
GAAAAAATGGATATGCGCGAACCACAATGTGTGGACGATGTGTTATCTGTTGATGCGAACGCGCG
TGAAGTCGCCAGAAAAGAGGTGATGCGTCTCGCAAGCTGACTCGAG

Figure S15. Bi-cistronic operon of 2-C-methyl-D-erythriol 4-phosphate cytidyltransferase synthase (*ispD*) 2-C-methyl-D-erythritol 2,4-cyclodiphosphate synthase (*ispF*).

<u>CCATGG</u>ATGGCAACCACTCATTTGGATGTTTGCGCCGTGGTTCCGGCGGCCGGATTTGGCCGTC
GAATGCAAACGGAATGTCCTAAGCAATATCTCTCAATCGGTAATCAAACCATTCTTGAACACTCGG
TGCATGCGCTGCTGGCGCATCCCCGGGTGAAACGTGTCGTCATTGCCATAAGTCCTGGCGATAG
CCGTTTTGCACAACTTCCTCTGGCGAATCATCCGCAAATCACCGTTGTAGATGGCGGTGATGAGC
GTGCCGATTCCGTGCTGGCAGGTTTGAAAGCCGCTGGCGACGCGCAGTGGGTATTGGTGCATG
ACGCCGCTCGTCCTTGTCTGCATCAGGATGACCTCGCGCGATTGTTGGCGTTGAGCGAAACCAG
CCGCACGGGAGGGATCCTAGCCGCACCAGTGCGCGATACGATGAAACGTGCCGAACCGGGCAA
AAATGCCATTGCTCATACCGTTGATCGCAACGGCTTATGGCACGCGCTGACGCCGCAATTTTTCC
CTCGTGAGCTGTTACATGACTGTCTGACGCGCGCTCTAAATGAAGGCGCGACTATTACCGACGAA
GCCTCGGCGCTGGAATATTGCGGATTCCATCCTCAGTTGGTCGAAGGCCGTGCGGATAACATTA
AAGTCACGCGCCCGGAAGATTTGGCACTGGCCGAGTTTTACCTCACCCGAACCATCCATCAGGA
GAATACATAAGCAGGAGCAGGAGCAGAAGGAGGAGCAGGAATGCGAATTGGACACGGTTTTGAC
GTACATGCCTTTGGCGGTGAAGGCCCAATTATCATTGGTGGCGTACGCATTCCTTACGAAAAAGG
ATTGCTGGCGCATTCTGATGGCGACGTGGCGCTCCATGCGTTGACCGATGCATTGCTTGGCGCG
GCGGCGCTGGGGGATATCGGCAAGCTGTTCCCGGATACCGATCCGGCATTTAAAGGTGCCGAC
AGCCGCGAGCTGCTACGCGAAGCCTGGCGTCGTATTCAGGCGAAGGGTTATACCCTGGGCAAC
GTCGATGTCACTATCATCGCTCAGGCACCGAAGATGTTGCCGCACATTCCACAAATGCGCGTATT
TATTGCCGAAGATCTCGGCTGCCATATGGATGATGTTAACGTGAAAGCCACTACTACGGAAAAAC
TTGGATTTACCGGACGTGGGGAAGGGATTGCCTGTGAAGCGGTGGCGCTACTCATTAAGGCAAC
AAAATGA<u>GAATTC</u>

Figure S16. Gene of Isopentenyl-diphosphate delta isomerase (*Idi*).

<u>CATATG</u>CAAACGGAACACGTCATTTTATTGAATGCACAGGGAGTTCCCACGGGTACGCTGGAAAA
GTATGCCGCACACACGGCAGACACCCGCTTACATCTCGCGTTCTCCAGTTGGCTGTTTAATGCCA
AAGGACAATTATTAGTTACCCGCCGCGCACTGAGCAAAAAAGCATGGCCTGGCGTGTGGACTAA
CTCGGTTTGTGGGCACCCACAACTGGGAGAAAGCAACGAAGACGCAGTGATCCGCCGTTGCCGT
TATGAGCTTGGCGTGGAAATTACGCCTCCTGAATCTATCTATCCTGACTTTCGCTACCGCGCCAC
CGATCCGAGTGGCATTGTGGAAAATGAAGTGTGTCCGGTATTTGCCGCACGCACCACTAGTGCG
TTACAGATCAATGATGATGAAGTGATGGATTATCAATGGTGTGATTTAGCAGATGTATTACACGGT
ATTGATGCCACGCCGTGGGCGTTCAGTCCGTGGATGGTGATGCAGGCGACAAATCGCGAAGCCA
GAAAACGATTATCTGCATTTACCCAGCTTAAATAA<u>CTCGAG</u>

Figure S17. Gene of *cotB2.*

CATATGACCACCGGTCTGAGCACCGCAGGCGCACAGGATATTGGTCGTAGCAGCGTTCGT
CCGTATCTGGAAGAATGTACCCGTCGTTTTCAAGAAATGTTTGATCGTCATGTTGTTACC
CGTCCGACCAAAGTTGAACTGACCGATGCAGAACTGCGTGAAGTTATTGATGATTGTAAT
GCAGCAGTTGCACCGCTGGGTAAAACCGTTAGTGATGAACGTTGGATTAGCTATGTTGGT
GTTGTTCTGTGGTCACAGAGTCCGCGTCATATTAAAGATATGGAAGCATTTAAAGCCGTG
TGCGTTCTGAATTGTGTTACCTTTGTTTGGGATGATATGGACCCTGCACTGCATGATTTT
GGTCTGTTTCTGCCTCAGCTGCGTAAAATTTGCGAAAAATACTATGGTCCGGAAGATGCC
GAAGTTGCCTATGAAGCAGCACGTGCATTTGTTACCAGCGATCACATGTTTCGTGATAGC
CCGATTAAAGCAGCACTGTGTACCACCAGTCCGGAACAGTATTTTCGTTTTCGTGTTACC
GATATTGGCGTGGATTTTTGGATGAAAATGAGCTATCCGATTTATCGCCATCCGGAATTT
ACCGAACATGCAAAAACCAGCCTGGCAGCACGTATGACCACCCGTGGTCTGACCATTGTT
AATGATTTCTATAGCTATGATCGCGAAGTTAGCCTGGGTCAGATTACCAATTGTTTTCGT
CTGTGTGATGTGAGTGATGAAACCGCCTTTAAAGAATTTTTTCAGGCACGTCTGGATGAC
ATGATCGAAGATATTGAATGCATCAAAGCGTTTGATCAGCTGACACAGGATGTTTTTCTG
GATCTGATTTATGGCAATTTTGTGTGGACCACCTCCAACAAACGTTATAAAACCGCAGTG
AATGATGTGAACAGCCGTATTCAATAACTCGAG

Figure S18. Bi-cistronic *cotB3*/*cotB4* operon.

CATATGCGTGAACGTGGTCCGGTTACACCGGCAAAAAGCAGCGCACCGCCTGAACGTCCG
TGGACCACCGGTACAGCACCGGGTAGCGTTCCGCTGCTGGGTCATACAATGGCACTGTGG
CGTCGTCCGCTGCAATTTCTGGCAAGCCTGCCTGCACATGGTGATCTGGTTGAAGTTCGT
CTGGGTCCGAGCCGTGCATATCTGGCATGTCATCCGGAACTGGTTCGTCAGGTTCTGCTG
AATCCGCGTGTTTTTGATAAAGGTGGCGTTTTCGATAAAGCACGTCAGCTGCTGGGCAAT
AGCCTGAGCGTTAGCCGTGGTGAAGATCATCGTTATCAGCGTCGTATGATTCAGCCTGCA
TTTCATACCCCGAAAATTGCCGCATATACCGCAGCAGTTGCAGATGATACCCGTGCAGCA
ATTGGTAGCTGGGAACCGGGTCGTACCCTGGATATTAGCGATACCATGCATGCACTGCTG
ATGCGTGTTGCAGCACGTACCCTGTTTAGCACCGGTATTGATGAAGCAACCATCGATGAA
GCCCGTCATTGTCTGCGTATTGTTAGTGATGGTATCTATAAACGTACGATGGCACCGCTG
GGTATTATGGAAAAACTGCCGACACCGGGTAATCGTCGTTATGATCGTGCAAATGCACGT
CTGCGTCAGATTGTTGATGAAATGATTCGTGAACGTCGTCGTAGCGGTGCAGATCATGGC
GATCTGCTGAGCACCCTGCTGCGTGCAGAACATCCGGAAACCGGTAAAGGTCTGGATGAT
GGTGAAGTTCTGGATCAGGTTGTTACCTTTCTGGTTGCAGGTAGCGAAACCACCGCAAGC
ACCCTGGCATTTGTTTTTCATCTGCTGGGTGCCCATCCGGAAGTTGAAAAACGTGTTCAT
GCAGAAATCGATGAAATTCTGGAAGGTCGTAGCCCGACCTTTGAAGATCTGCCGAGCCTG
GAATATACCCGTGGTGTTATTACCGAAAGCCTGCGTCTGTATCCGCCTAGCTGGATGGCA
ATGCGTGTTACCGCAGCCGAAACCGAACTGGGTGGTCGTACCGTTCCGGCAGGCACCATG
ATTCTGTATAGCGCACAGGCACTGCATCATAACCCGGAACTGTTTCCAGATCCGGAACGT
TTTGATCCTGAACGTTGGCTGGGTGATCGTGCCAAAGAAGTTGAACGCGGAGCACTGCTG
CCGTTTGGTGCCGGTAGCCATAAATGTATTGGTGATGTGCTGGCACTGACCGAAACAGCA
CTGATTGTTGCAACCATTGCAAGCCGTTGGCGTCTGCGTCCGGTTCCGGGTACAACCCTG
CGTCCTGAACCGAAAGCAACCCTGGAACCTGGTCCGCTGCCGATGGTTTGTGAACCGCGT
TAACTAGTAGGAGGAAAACATCATGAAAGATTTTTTTCGTATGCGCACCGCACAGCAGCC
TGCCACACGTCATTGGCGTCATACCGTTGCACCGGGTGGTCTGCCGCTGGCAGGTCACGC
TCTGCTGATGGCACGTAAACCTCTGCAATTCCTGGCCTCACTGCCAGCCCACGGCGATCT
GGTGGAACTGCGCCTGGGACCGCGTCCGGTGTATCTGCCGTGCCACCCTGAACTGGTGCA
GCAGGTACTGGTTAATGCACGTGTTTATGATACAGGCGGTCCGGTGAAAGAAAAAGCAAA
ACCGATTCTGGGTAATGGTCTGATTACCAGCGATTGGGCTGATCATCGTCGCCAGCGTCG
CCTGGTTCAGCCAGCCTTTCACACCGCACGTATTGCAAAGTATGCCGAAGTTATGGAACG
TGAATGTGAAGCAGAAAGCACCGCATGGACCGCACGTCGTCCGATTGATGTTAGCCATGA
AATGCTGGCCCTGACAGCACGCGTTACCGCACGTGCACTGTTTTCAACCGATATGGCTCC
GCATGCCGTTGCAGAAATTCAGCATTGCCTGCCGATTGTTGTTGAAGGTGCATATCGTCA
GGCAATTGATCCGACCGGTCTGCTGGCCAAACTGCCTCTGGCAGCAAATCGTCGCTTTGA
TGATGCACTGGCACGTCTGAACCAGCTGATTGATCGCATGATTGATGATTACAAAGCCAG
TGATGATGGCGATCGTGGTGATGTTCTGAGCGCACTGTTTGCAGCACAGGATGATGAAAC
CGGTGGTACAATGAGCGATCAAGAAATTCATGATCAGGTTATGACACTGCTGCTGGCTGG
TATTGAAACCACAGCCAGCGCACTGACCTGGGCATGGTTTCTGCTGGGACGTAATCCGGG
TGCGGAAGCAGCACTGCATGCGGAAGTGGATGAAGTGCTGGGTGGCCGTGCACCGCGTTA
TGCAGATGTTCCGCGTCTGGCATATACACAGCGTGTGTTTAGCGAAGCCCTGCGCCTGTT
TCCTCCGGCATGGCTGTTTACCCGTACCACCACCGAAACCACGGAACTGGGAGGCCGTCG
TCTGCCTCCGGCTTCAGATGTGCTGATTAGCCCGTATGTGCTGCATCGTGATCCAGCACT
GTTTCCGCGTCCTGATAGCTTTGACCCGGATCGTTGGCTGCCAGAACGCGCAAAAGAAGT
AACACGCGGTAGCTATCTGCCTTTTGGTGGTGGTTCACGTAAATGCATTGGCGACGTTTT
TGGTATGACCGAAGCAACACTGGCACTGGCAGCGATTGCCGGTCGTTGGCGGATGCGTCC
TATTCCTGGCACCAAAATTCGTCCGCGTCCGCAGATGAGCCTGACCGCAGGTCCTCTGCG
CATGATTCCGGAACCTCGTTAACTCGAG

Figure S19. Bi-cistronic *afR*/*afx* operon.

CCATGGGCCCTGCAAATGATGCCTGTGTTATTGTTGGTGCAGGTCTGGCAGGCGCAAAAG
CAGCACAGGCACTGCGTGAAGAGGGTTTTGATGGTCCGCTGGTTCTGATTGGTGATGAAC
GTGAACGTCCGTATGAACGTCCTCCGCTGAGCAAAGGTTATCTGACCGGTAAAGATGCAC
GTGAGCAGATTTATGTTCATCCGCCTCAGTGGTATGCAGAACATAATGTTGATATGCGTC
TGGGTATGGCAGTTACCGCAGTTGATCCGGCAGCACGTGAAATGACCCTGGATGATGGTA
GCCGTGTTGGTTATGGTAAACTGCTGCTGACCACCGGTAGCGCACCGCGTCGTCTGCCGG
TTCCTGGTGCTGGCCTGGAACGTGTTCTGTATCTGCGTCGTGTTGAAGATAGCGATCAGA
TTAAAGAAGCATTTCAGAGCGCAAGCCGTGCAGTTGTTATTGGTGCCGGTTGGATTGGTC
TGGAAACCACCGCAGCAGCACGTACCGCAGGCGTTGAAGTTACCGTTCTGGAAATGGCAG
AACTGCCGCTGCTGCGTGTTCTGGGTCGTGAAGTTGCACAGCTGTTTGCAAATCTGCATC
GTGATCATGGTGTTGATCTGCGTTTTGGTGCACAGGTTGCAGAAATTACCGGTAGTGGTG
GTGCAGTTGATGGTGTTCGTCTGAGTGATGGCACCCGTATTGATGCAGATGTTGTGATTG
TGGGTGTTGGCATTAAACCGAATATTGGCCTGGCACAAGAAGCCGGTCTGGAAGTTGATA
ATGGTATTCGTGTGGATGAACGTCTGCGTACCAGCTATCCTGATATTTATGCAGCCGGTG
ATGTTGCACATGCATTTCATCCGCTGCTGGGTAAACATATTCGCGTTGAACATTGGGCAA
ATGCACTGAATCAGCCGCAGATTGCAGCAAAAGCAATGCTGGGTCGCGAAGATGCCGTTT
ATGATCGTATTCCGTATTTTTTCACCGATCAGTATGATCTGGGCATGGAATATGCAGGTT
ATGTTGAACCGGGTGGTTATGATCAGGTTGTTTTTCGTGGTGATGTGGCAGGTCGTGAAT
TTATTGCATTTTGGCTGGCAGGCAATCGTGTGCTGGCAGGTATGAATGTGAATATTTGGG
ATGTTAATGATCAGCTGCAGACCCTGGTTCGTACCGCACAGACCGTTGATATTCCGATGC
TGACCGATCCGCAGGTTCCGCTGGGTAGCCTGCTGCCTGATCCTCAGCATCGTTAATATA
TTAGTTAAGTATAAGAAGGAGATATAATGCCGAAAGTTACCTATGTTAGTGATGCCGGTG
AAGTTCGTGTTGTTGATGGTCTGGTTGGTGATAGCGTTATGCAGACCGCAGTTCGTAATG
GTGTTCCGGGTATTACCGGTGAATGTGGTGGTGTTCTGAGCTGTGCAACCTGTCATGTTT
TTGTTGATGAAGCAGATCTGGATCGTCTGGAACCGGTTAGCGGTCTGGAAGATGAAATGC
TGGATGGCACCGTTGTTGATCGTTGTCCGAATAGCCGTCTGAGCTGCCAGATTAAACTGA
GCGAAGAACTGGGTGATCTGCGTGTTACCACACCGGAAGCACAAGAATAAGCGGCCGC

Figure S20. Bi-cistronic *pdR*/*pdx* operon.

CCATGGGCAACGCCAATGATAATGTTGTTATTGTTGGCACCGGTCTGGCAGGCGTTGAAGTTGCA
TTTGGTCTGCGTGCAAGCGGTTGGGAAGGTAATATTCGTCTGGTTGGTGATGCAACCGTTATTCC
GCATCATCTGCCTCCGCTGAGCAAAGCATATCTGGCAGGTAAAGCAACCGCAGAAAGCCTGTAT
CTGCGTACACCGGATGCCTATGCAGCACAGAATATTCAGCTGCTGGGTGGTACACAGGTTACCG
CAATTAATCGTGATCGTCAGCAGGTTATTCTGAGTGATGGTCGTGCACTGGATTATGATCGTCTG
GTGCTGGCAACCGGTGGTCGTCCGCGTCCGCTGCCGGTTGCAAGTGGTGCAGTTGGTAAAGCC
AATAACTTTCGTTATCTGCGCACCCTGGAAGATGCAGAATGTATTCGTCGTCAGCTGATTGCAGAT
AATCGCCTGGTTGTGATTGGTGGTGGTTATATTGGTCTGGAAGTTGCAGCAACCGCCATTAAAGC
AAATATGCATGTTACCCTGCTGGATACCGCAGCACGTGTTCTGGAACGTGTTACCGCACCGCCTG
TTAGCGCCTTTTATGAACATCTGCATCGTGAAGCCGGTGTTGATATTCGTACCGGCACCCAGGTT
TGTGGTTTTGAAATGAGCACCGATCAGCAGAAAGTTACCGCAGTTCTGTGTGAAGATGGCACCCG
TCTGCCTGCAGATCTGGTTATTGCAGGTATTGGCCTGATTCCGAATTGTGAACTGGCAAGCGCAG
CAGGTCTGCAGGTTGATAATGGTATTGTTATTAACGAACACATGCAGACCAGCGATCCGCTGATT
ATGGCAGTTGGTGATTGTGCACGTTTTCATAGCCAGCTGTATGATCGTTGGGTTCGTATTGAAAG
CGTTCCGAATGCACTGGAACAGGCACGTAAAATTGCAGCAATTCTGTGTGGTAAAGTTCCGCGTG
ATGAAGCAGCACCGTGGTTTTGGAGCGATCAGTATGAAATTGGTCTGAAAATGGTTGGTCTGAGC
GAAGGTTATGATCGCATTATTGTTCGTGGTAGCCTGGCACAGCCGGATTTTTCAGTTTTTTATCTG
CAGGGTGATCGTGTGCTGGCAGTTGATACCGTTAATCGTCCGGTTGAATTTAACCAGAGCAAACA
AATTATCACCGATCGTCTGCCGGTGGAACCGAATCTGCTGGGAGATGAAAGCGTGCCGCTGAAA
GAAATTATTGCAGCAGCAAAAGCAGAACTGAGCAGCGCATAATATATTAGTTAAGTATAAGAAGGA
GATATAATGAGCAAAGTTGTGTATGTTAGCCATGATGGCACCCGTCGTGAACTGGATGTTGCAGA
TGGTGTGAGCCTGATGCAGGCAGCAGTTAGCAATGGTATTTATGATATTGTTGGTGATTGTGGTG
GTAGCGCAAGCTGTGCAACCTGTCATGTTTATGTTAATGAAGCCTTCACCGATAAAGTTCCGGCA
GCAAATGAACGTGAAATTGGTATGCTGGAATGTGTTACCGCAGAACTGAAACCGAATAGCCGTCT
GTGTTGTCAGATTATTATGACACCGGAACTGGACGGTATTGTTGTTGATGTTCCGGATCGTCAGT
GGTAAGCGGCCGC

Figure S21.(-)-casbene synthase (*cs*) from *Jatropha curcas.*

<u>CATATG</u>GCAAGCACCAAAAGCGAAACCGAAGCACGTCCGCTGGCATATTTTCCGCCTACCGTTTG
GGGTGATCGTCTGGCAAGCCTGACCTTTAATCAGCCTGCATTTGAACTGCTGAGTAAACAGGTTG
AGCTGCTGAACGAGAAAATCAAAAAAGAAATGCTGAATGTGAGCACCAGCGATCTGGCAGAAAAA
ATCATTCTGATTGATAGCCTGTGTCGTCTGGGTGTTAGCTATCATTTTGAAGAAGAAATCCAAGAA
AACCTGACCCGCATCTTTAATACCCAGCCGAATTTTCTGAACGAAAAAGATTATGATTTATTCACC
GTGGCCGTGATCTTTCGTGTTTTTCGTCAGCATGGCTTTAAAATCAGCAGCGACGTTTTTAACAAA
TTCAAAGATAGCGACGGGAAATTCAAAGAAAGCCTGCTGAATGATATTAAAGGCATCCTGAGCCT
GTTTGAAGCAACCCATGTTAGCATGCCGAATGAACCGATTCTGGATGAAGCACTGGCATTTACCA
AAGCATTTCTGGAAAGCAGCGCAGTTAAATCCTTTCCGAATTTTGCCAAACATATTAGCAGCGCAC
TGGAACAGCCGGTTCATAAAGGTATTCCGCGTCTGGAAGCACGCAAATATATCGATCTGTATGAA
GTTGATGAAAGCCGCAATGAAACCGTGCTGGAACTGGCGAAACTGGATTTTAATCGTGTTCAGCT
GCTGCATCAAGAAGAACTGAGCCAGTTTAGCAAATGGTGGAAAAGCCTGAATATTAGTGCCGAAG
TTCCGTATGCACGTAATCGTATGGCAGAAATCTTTTTTTGGGCAGTGAGCATGTATTTTGAACCGC
AGTATGCAAAAGCCCGTATGATTGTTAGCAAAGTGGTTCTGCTGATTAGCCTGATTGATGATACCA
TTGATGCCTATGCCACCATCGATGAAATCCATCGTGTTGCAGATGCAATTGAACGTTGGGATATG
CGTCTGGTTGATCAGCTGCCGAATTATATGAAAGTGATTTATCGCCTGATCATCAACACCTTTGAT
GAGTTTGAAAAAGATCTGGAAGCCGAGGGTAAAAGCTATAGCGTTAAATATGGTCGTGAAGCCTA
TCAAGAACTGGTGCGTGGTTATTACCTGGAAGCAATTTGGAAAGCGGATGGTAAAGTTCCGAGCT
TCGATGAGTATATCTATAATGGTGGTGTTACCACCGGTCTGCCGCTGGTTGCCACCGTTAGCTTT
ATGGGTGTTAAAGAAATCAAAGGCACCAAAGCCTTTCAGTGGCTGAAAACCTATCCGAAACTGAA
TCAGGCAGGCGGTGAATTTATTCGTCTGGTTAATGATGTTATGAGCCATGAAACCGAACAGGATC
GTGGTCATGTTGCAAGCTGTATTGATTGCTATATGAAACAGTATGGCGTGAGCAAAGAAGAAGCC
GTTGAAGAGATCCAGAAAATGGCAACCAATGAGTGGAAAAAACTGAACGAACAGCTGATTGTTCG
TAGCACCGAAGTTGTTCCGGTTAATCTGCTGATGCGTATTGTTAATCTGGTTCGTCTGACCGATGT
GAGCTATAAATACGGTGATGGTTATACCGATAGCTCCCAGCTGAAAGAATATGTGAAAGGCCTGT
TTATTGAACCGATCGCAACCGGTACCTCTTAA<u>CTCGAG</u>

Figure S22. Taxadiene synthase (*txs*) from *Taxus brevifolia.*

CATATGGCAATGAGCAGCAGCACCGGCACCAGCAAAGTTGTTAGCGAAACCAGCAGTACCATTG
TTGATGATATTCCGCGTCTGAGCGCAAATTATCATGGTGATCTGTGGCATCATAATGTGATTCAGA
CCCTGGAAACCCCGTTTCGTGAAAGCAGCACCTATCAAGAACGTGCAGATGAACTGGTTGTGAAA
ATCAAAGATATGTTTAACGCACTGGGTGATGGTGATATTAGCCCGAGCGCCTATGATACCGCATG
GGTTGCACGTCTGGCAACCATTAGCAGTGATGGTAGCGAAAAACCGCGTTTTCCGCAGGCACTG
AATTGGGTTTTTAACAATCAGCTGCAGGATGGTAGTTGGGGTATTGAAAGCCATTTTAGCCTGTGT
GATCGTCTGCTGAATACCACCAATAGCGTTATTGCACTGAGCGTTTGGAAAACCGGTCATAGCCA
GGTTCAGCAGGGTGCAGAATTTATTGCAGAAAATCTGCGCCTGCTGAATGAAGAAGATGAGCTGA
GTCCGGATTTTCAGATTATCTTTCCGGCACTGCTGCAGAAAGCAAAAGCACTGGGTATTAATCTG
CCGTATGATCTGCCGTTTATCAAATATCTGAGCACCACCCGTGAAGCACGTCTGACCGATGTTAG
CGCAGCAGCAGATAATATTCCGGCAAATATGCTGAATGCACTGGAAGGTCTGGAAGAAGTTATTG
ACTGGAACAAAATTATGCGCTTCCAGAGCAAAGATGGTAGCTTTCTGAGTAGTCCGGCAAGCACC
GCATGTGTTCTGATGAATACCGGTGATGAAAAATGCTTTACCTTCCTGAATAACCTGCTGGATAAA
TTTGGTGGTTGTGTTCCGTGTATGTATAGCATTGATCTGCTGGAACGTCTGAGCCTGGTTGATAAT
ATTGAACATCTGGGTATTGGTCGCCACTTCAAACAAGAAATTAAAGGTGCACTGGATTACGTGTAT
CGTCATTGGAGCGAACGTGGTATTGGTTGGGGTCGTGATAGCCTGGTTCCGGATCTGAATACAA
CCGCACTGGGCCTGCGTACCCTGCGTATGCATGGTTATAATGTTAGCTCAGATGTGCTGAACAAC
TTTAAAGATGAAAACGGTCGCTTTTTTAGCAGCGCAGGTCAGACCCATGTTGAACTGCGTAGCGT
TGTTAACCTGTTTCGTGCAAGCGATCTGGCATTTCCGGATGAACGTGCAATGGATGATGCACGTA
AATTTGCAGAACCGTATCTGCGTGAAGCCCTGGCCACCAAAATTAGCACCAATACAAAACTGTTTA
AAGAAATCGAATATGTGGTCGAGTATCCGTGGCACATGAGCATTCCTCGTCTGGAAGCACGTAGC
TATATTGATAGCTATGATGATAACTATGTGTGGCAGCGTAAAACCCTGTATCGTATGCCGAGCCTG
AGCAATAGCAAATGTCTGGAACTGGCAAAACTGGATTTTAACATTGTTCAGAGCCTGCACCAAGA
AGAACTGAAACTGCTGACCCGTTGGTGGAAAGAAAGCGGTATGGCAGATATTAACTTTACCCGTC
ATCGTGTTGCCGAAGTGTATTTTAGCAGTGCAACCTTTGAACCGGAATATAGCGCAACCCGTATT
GCCTTTACCAAAATTGGTTGTCTGCAGGTCCTGTTCGATGATATGGCCGATATTTTTGCAACCCTG
GATGAACTGAAAAGTTTTACCGAAGGTGTTAAACGTTGGGATACCAGTCTGCTGCATGAAATCCC
GGAATGTATGCAGACCTGTTTTAAAGTGTGGTTTAAACTGATGGAAGAGGTGAATAACGATGTGG
TTAAAGTTCAGGGTCGCGATATGCTGGCCCATATTCGTAAACCGTGGGAACTGTATTTCAACTGC
TATGTTCAAGAACGCGAATGGCTGGAAGCCGGTTATATTCCGACCTTTGAAGAATATCTGAAAAC
CTATGCAATTAGCGTTGGTCTGGGTCCGTGTACCCTGCAGCCGATTCTGCTGATGGGTGAACTG
GTGAAAGATGATGTTGTTGAGAAAGTTCATTACCCGAGCAACATGTTTGAACTGGTAAGCCTGAG
CTGGCGTCTGACCAATGATACCAAAACCTATCAGGCAGAAAAAGCACGTGGTCAGCAGGCAAGC
GGTATTGCATGTTATATGAAAGACAATCCGGGTGCAACCGAAGAGGATGCAATCAAACATATTTG
TCGTGTTGTTGATCGTGCACTGAAAGAAGCCAGCTTTGAATATTTCAAACCGAGCAACGATATTCC
GATGGGCTGTAAATCCTTTATCTTTAATCTGCGTCTGTGCGTGCAGATCTTCTATAAATTCATTGAT
GGTTACGGCATTGCCAACGAAGAGATCAAAGATTATATCCGCAAAGTGTATATCGATCCGATTCA
GGTTTAACTCGAG
